# Chemical Characteristics of Ciprofloxacin and Its Degradation Products: On the Road to Understanding Its Potential Hazards

**DOI:** 10.1021/acsomega.5c06831

**Published:** 2025-11-27

**Authors:** Alexis Caballero, Emiliano Perez-Sanchez, Amauri Serrano-Lázaro, Monserrat Bizarro, Ana Martínez

**Affiliations:** Departamento de Materiales de Baja Dimensionalidad, Instituto de Investigaciones en Materiales, Universidad Nacional Autónoma de México, México CDMX 04510, México

## Abstract

Antibiotics such as ciprofloxacin (CIP) are considered emerging contaminants because of their widespread use in human and veterinary medicine, their persistence in the environment, and their incomplete removal during wastewater treatment. To mitigate pollution, the photocatalytic degradation of antibiotics is an available option. However, some of the degradation products could be more harmful than the original compound. The main idea of this investigation is to determine the chemical properties of the main photodegradation products of CIP to understand the positive or negative consequences of degrading CIP. Density functional theory calculations were performed to investigate the electron transfer capacity of CIP and 20 selected degradation products. Fukui functions were also calculated to determine the reactive atoms. To identify the degradation products, this investigation presents theoretical and experimental ultraviolet (UV)/visible spectra of the photocatalytic degradation of CIP using zinc oxide nanowires (ZnO NW). The 3 h photocatalytic reaction showed an 89% of CIP degradation, indicating the effectiveness of ZnO NW photocatalyst. The agreement between the experiment and theory indicates that two degradation products (B5 and B8) are formed during the photocatalytic experiment. Concerning the electron transfer capacity, B8 is a better electron donor and could help to prevent oxidative stress by donating electrons to free radicals. B5 is a better electron acceptor, but it is not as good as ^•^OOH, and therefore, it is not expected that this molecule will oxidize other molecules. Fukui functions reveal that B5 and B8 have more reactive atoms than CIP, and this could have consequences for health and the environment. With this information on the chemical characteristics of the degradation products, it is possible to determine whether or not it is advisable to degrade the CIP using this photocatalytic degradation procedure, and the risks of not achieving its complete mineralization. Understanding the chemical reactivity from these theoretical results facilitates the analysis of the potential hazards in subsequent studies.

## Graphical abstract

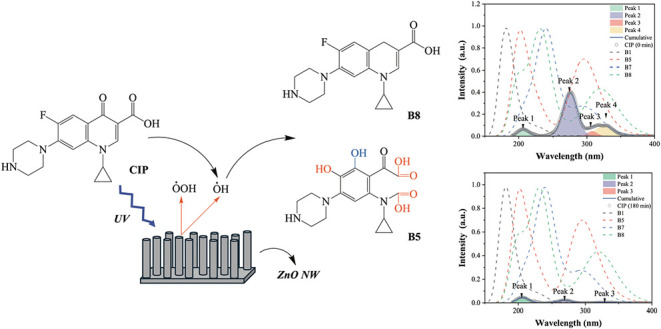

## Introduction

The enormous amount of antibiotics currently consumed to prevent and treat infections in humans and animals is beginning to represent a serious contamination problem. This is because antibiotics are not fully metabolized and consumers excrete both intact and metabolized pharmaceuticals, many of which are water-soluble.^1–8^ These compounds can reach aquatic environments, increasing the number of contaminants in the surface and groundwater, as well as in sewage treatment plant effluents.^3,7,8^ These residues are not easily eliminated in most wastewater treatment processes and, in fact, these compounds are beginning to be considered as emerging pollutants.^5^ An example of a widely used antibiotic is ciprofloxacin (CIP), presented in Figure 1, which belongs to the fluoroquinolone family. Specifically, CIP is a broad-spectrum antibiotic used to treat various bacterial infections. Its mechanism of action is to inhibit bacterial DNA replication.^9,10^ There are several methods to remove CIP from water, such as adsorption, electrochemical, membrane separation, phytoremediation, and advanced oxidation processes.^11–21^ Previous studies on the microbial transformation and degradation of fluoroquinolones present an overview of the main biotransformation products of ciprofloxacin.^15^ Among all the methods to remove CIP, advanced oxidation processes appear to be a promising alternative, since CIP is destroyed until its complete mineralization.^13^ One of these advanced oxidation processes to remove CIP from water is photocatalysis, which requires a semiconductor material activated with light to produce reactive oxygen species that then participate in redox reactions to degrade CIP. Photocatalytic degradation of CIP occurs gradually and generates several degradation products.^14,16–21^ Previous studies^19^ indicate that the first photodegradation of CIP occurs at different sections of the molecule, as shown in Figure 1.

**Figure 1:**
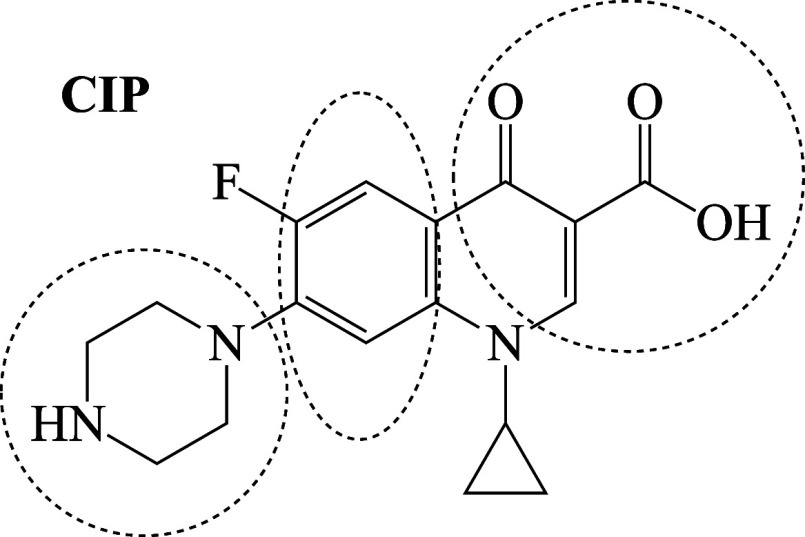
Photodegradation sites of ciprofloxacin (CIP) with free radicals.

There are previous molecular dynamics simulations and quantum chemical calculations about detailed insights into the adsorption mechanisms, reaction pathways, and the energies involved in the oxidation processes.^13,22–26^ These results help us to design more effective and efficient strategies to mitigate pollution. Computational studies are also important to understand, at a molecular level, the interactions between CIP and remediation agents.^22,23^ Likewise, there are investigations concerning the interaction of CIP with the active site of DNA gyrase.^9^ Despite all these reports, there are still few works focused on theoretical and experimental studies of the chemical properties or toxicity of ciprofloxacin’s degradation products. This is crucial because, although contaminant degradation is important for pollution prevention, it is necessary to know whether the degradation products react differently from the original products. Degradation could produce more reactive and therefore potentially more toxic compounds. For instance, there are previous studies analyzing the potential toxicity of nanoplastics, where the authors report that degradation products are at least as toxic as nanoplastics.^27,28^

For CIP, several degradation products have been characterized^19,29–33^ and the toxicity of some biotransformation products has also been previously described,^33^ but these products differ from those investigated in this work. Despite all this previous research, there are no studies that analyze the reactivity of degradation products as we present in this study. The principal objective of these investigations is to analyze the chemical properties of the main ciprofloxacin degradation products. To understand their reactivity, we analyzed the electron transfer capacity and Fukui functions of ciprofloxacin and 20 select degradation products. The condensed Fukui function measures the ability of a specific point in a molecule to act as a nucleophilic, electrophilic, or free radical center during a chemical reaction. These parameters determine the chemical properties at a local level of CIP and its degradation products. During photocatalytic degradation, it is important to determine the products formed. Product identification is usually done by using liquid chromatography techniques. Nevertheless, these techniques require expensive inputs, such as high-purity solvents, columns, and detectors, as well as trained personnel to operate the instruments. One easy and common method used to evaluate the photocatalytic activity is UV/Visible spectroscopy. In this work, we report theoretical UV/Visible spectra of the main degradation products formed through ^•^OH radicals, as well as the experimental UV/Visible spectra of the photocatalytic degradation of CIP using zinc oxide nanowires (ZnO NW) films. This nanostructured photocatalyst was chosen as it has demonstrated very high photocatalytic activity for the degradation of other toxic compounds such as the temephos pesticide.^34^ The experimental and theoretical spectra were compared to identify the degradation products. The agreement between them allowed us to determine the degradation products formed during this photocatalytic process. With information on the chemical characteristics of the degradation products formed, we can determine whether it is convenient to degrade CIP using this photocatalytic degradation procedure. If the degradation products are more reactive and potentially more dangerous, then the degradation protocols should ensure the complete mineralization of CIP, using quantitative analytic techniques. Understanding the chemical reactivity from these theoretical results can aid in the analysis of potential toxicity in subsequent studies.

## Methods

### Computational Details

Gaussian16 was used for all electronic calculations.^35^ Geometry optimizations were obtained at the M06–2*X*/6–311+g (2d, p) level of theory without symmetry constraints.^36–38^ This exchange correlation functional has been used before with success. This is a global hybrid functional with 54% HF exchange, and it is the best within the M06 suite of functionals for main group thermochemistry, kinetics, and noncovalent interactions.^39,40^ TD-DFT calculations were used to simulate UV/Visible spectra. Unrestricted calculations were used, and local minima were identified by the number of imaginary frequencies (NIMAG = 0). The stationary points were modeled using the polarizable continuum model, specifically the integral-equation formalism (IEF-PCM) with water.^41^

The full electron donor–acceptor map (FEDAM) is a useful previously defined tool.^42,43^ In this map, vertical ionization energy (I) and vertical electron affinity (A) are plotted and allow us to classify substances as either donors or acceptors of electrons (Figure 2). Electrons will be transferred from good donor systems (down to the left) to good electron acceptor systems (up to the right of the map). FEDAM has been successfully used for different chemical systems.^44–47^ I and A are obtained as follows

1 $$Y\to Y^{+1}+1e^{-}I=E\left(Y^{+1}\right)-E\left(Y\right)$$

2 $$Y^{-1}\to Y+1e^{-}A=E\left(Y\right)-E\left(Y^{-1}\right)$$

**Figure 2:**
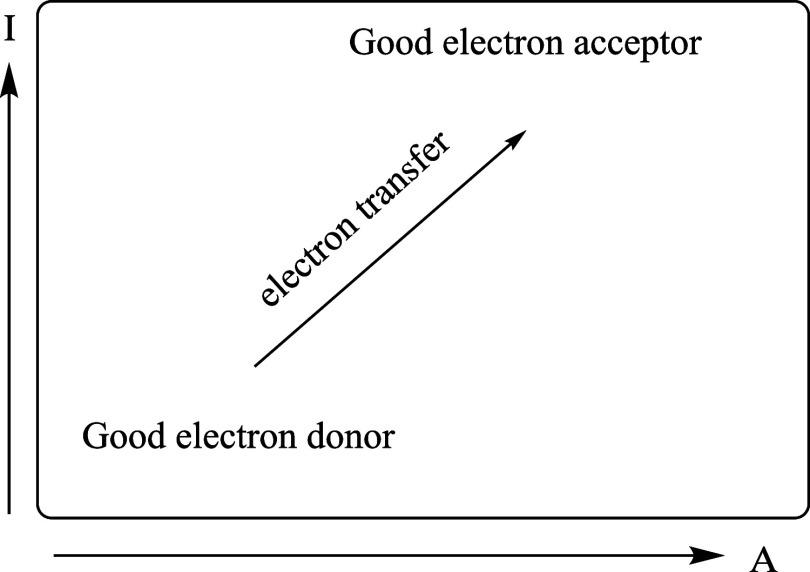
Full electron donor–acceptor map (FEDAM).

The condensed Fukui functions allow the identification of sections within a molecule that are accessible to nucleophilic, electrophilic, or free radical attacks. In a finite difference approximation,^48^ the Fukui functions are defined as follows

3 $$f\mathrm{or electrophilic attack}:f^{-}=q_{AK}\left(A\right)-q_{AK}\left(A^{+1}\right)$$

4 $$f\mathrm{or nucleophilic attack}:f^{+}=q_{AK}\left(A^{-1}\right)-q_{AK}\left(A\right)$$

5 for radical attack:f0=12[qAK(A+1)−qAK(A−1)]

In these equations, *q*_AK_ is the atomic charge of atom k in molecule A, A^+1^ is the cationic molecule, and A^–1^ is the anionic molecule. In this investigation, atomic charges are from a Mulliken population analysis.

### Synthesis of Photocatalytic ZnO Nanowires

Zinc oxide nanowire (ZnO NW) films were obtained and used as photocatalysts for the degradation of a CIP solution. The ZnO NW films were grown using the vapor–liquid–solid (VLS) technique as previously reported and described in detail in the Supporting Information.^34,49^ The films were widely characterized by X-ray diffraction (XRD), field emission scanning electron microscopy (FESEM), transmission electron microscopy (TEM), photoluminescence (PL), UV–vis spectroscopy, and X-ray photoelectron spectroscopy, as described in the Supporting Information.

### Photocatalytic Degradation of Ciprofloxacin

A 5 mg/L aqueous solution of CIP was prepared using Ciprofloxacin hydrochloride monohydrate (C_17_H_18_FN_3_O_3_·HCl·H_2_O, Sigma-Aldrich) in deionized water. A 2.5 × 1.25 cm^2^ size ZnO NW film was immersed in the solution and kept under dark conditions for 60 min with vigorous magnetic stirring (1200 rpm) to reach the adsorption–desorption equilibrium. Then, it was exposed to a UV lamp (26 W/m^2^) with the emission centered at 380 nm. The photocatalytic degradation of CIP was evaluated by measuring the absorbance spectrum of the solution as a function of the irradiation time, using a Shimadzu 1800 UV/Visible spectrophotometer. The degradation of the molecule was determined by

6 $$deg\;\%=\left(1-\frac{A_{t}}{A_{0}}\right)\times 100$$

where *A*_0_ is the absorbance of CIP’s main absorption band (at 276 nm) and *A*_*t*_ is the absorbance at the irradiation time *t*. To quantify the photocatalytic degradation, the concentration of CIP along the photocatalytic reaction was monitored by measuring high-performance liquid chromatography in tandem with triple quadrupole mass spectrometry (HPLC-MS/MS). An Infinity 1200 Agilent Technologies liquid chromatograph, equipped with a Zorbax SB C18 column (150 × 4.6 mm^2^ and 5 μm of stationary phase thickness), was used for the separation. The mobile phase was composed of a 70:30 acetonitrile:0.1 % (v/v) formic acid mixture, flowing at 0.4 mL min^–1^ in isocratic mode, with a time of analysis of 6 min. The injection volume was 5 μL. Chromatographic separation was followed by electrospray ionization in positive mode (ESI+), with N_2_ as the drying gas (300 °C and a flow rate of 11 L min^–1^). The capillary voltage was set at 4000 V, and the nebulizer pressure was 15 psi. Mass spectrometry analysis was performed in an Agilent Technologies 6420 device, where identification was performed in the Multiple Reaction Monitoring (MRM) mode. In the case of CIP, the precursor ion was *m*/*z* = 332.1 [M–H]^+^, and the product ions were *m*/*z* = 314.0 and *m*/*z* = 288.2.

## Results and Discussion

### Theoretical Study

#### First Degradation

Several degradation products have been reported.^19^ The labels we use here to identify the degradation products are those reported previously in ref 19. In this investigation, the so-called *first degradation* refers to the reaction of CIP with the ^•^OH free radical (the main reactive species formed in the ZnO photocatalyst, as will be shown later). These are considered the first products formed. Figure 3 shows the molecular formula of the first degradation products and the optimized geometries of each compound. Calculated UV/Visible spectra are presented in Figure 4. As can be seen, the maxima in the spectra of A19, XI, B13, XIII, A17, and A1′ are slightly different from those in the CIP spectrum. For CIP, it is 262 nm, while the values for the degradation products are 250 nm for A19, A1′ and XI; 268 nm for B13; and 256 nm for XIII and A17. In all cases, there is also a second signal of lower intensity, which is different for XI (202 nm), B13 (208 nm), A17, and A1’ (202 nm).

**Figure 3:**
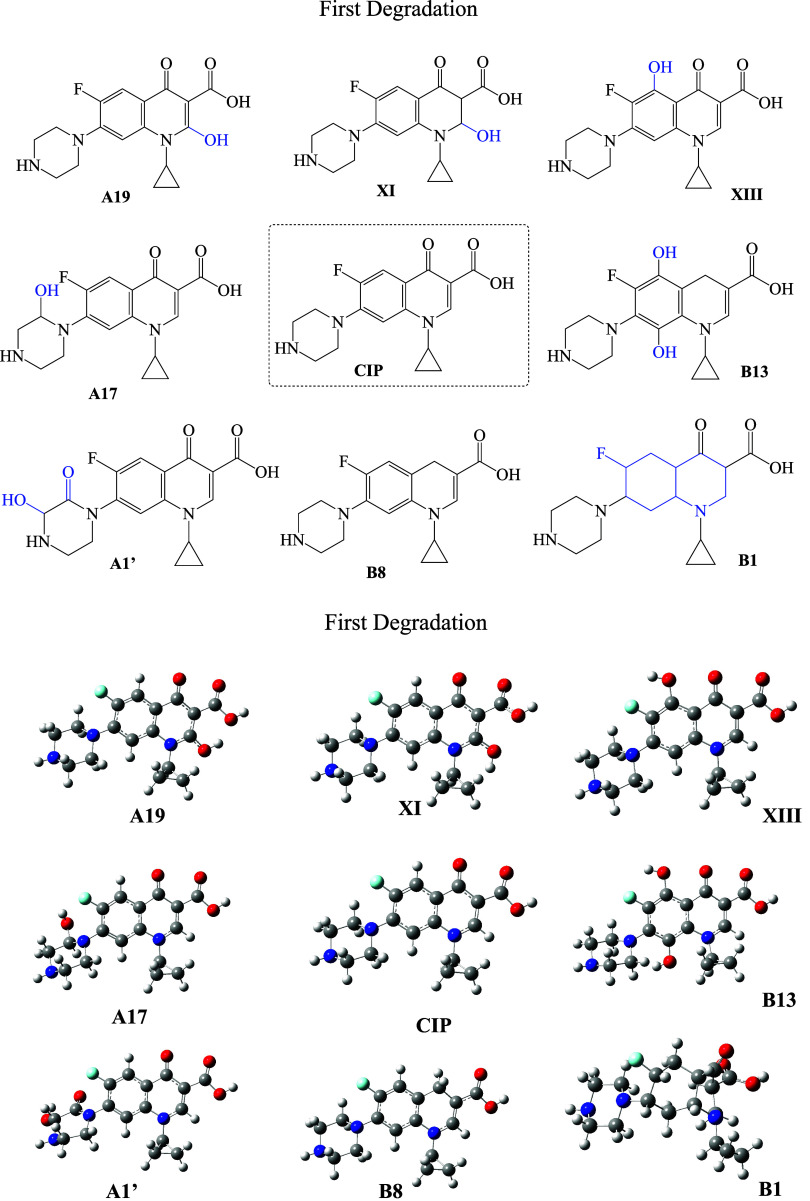
Schematic representation of first degradation products and CIP. Optimized geometries are also included. Carbon atoms are in gray, nitrogen atoms are in blue, oxygen atoms are in red, fluorine atoms are in light blue, and hydrogen atoms are in white.

**Figure 4:**
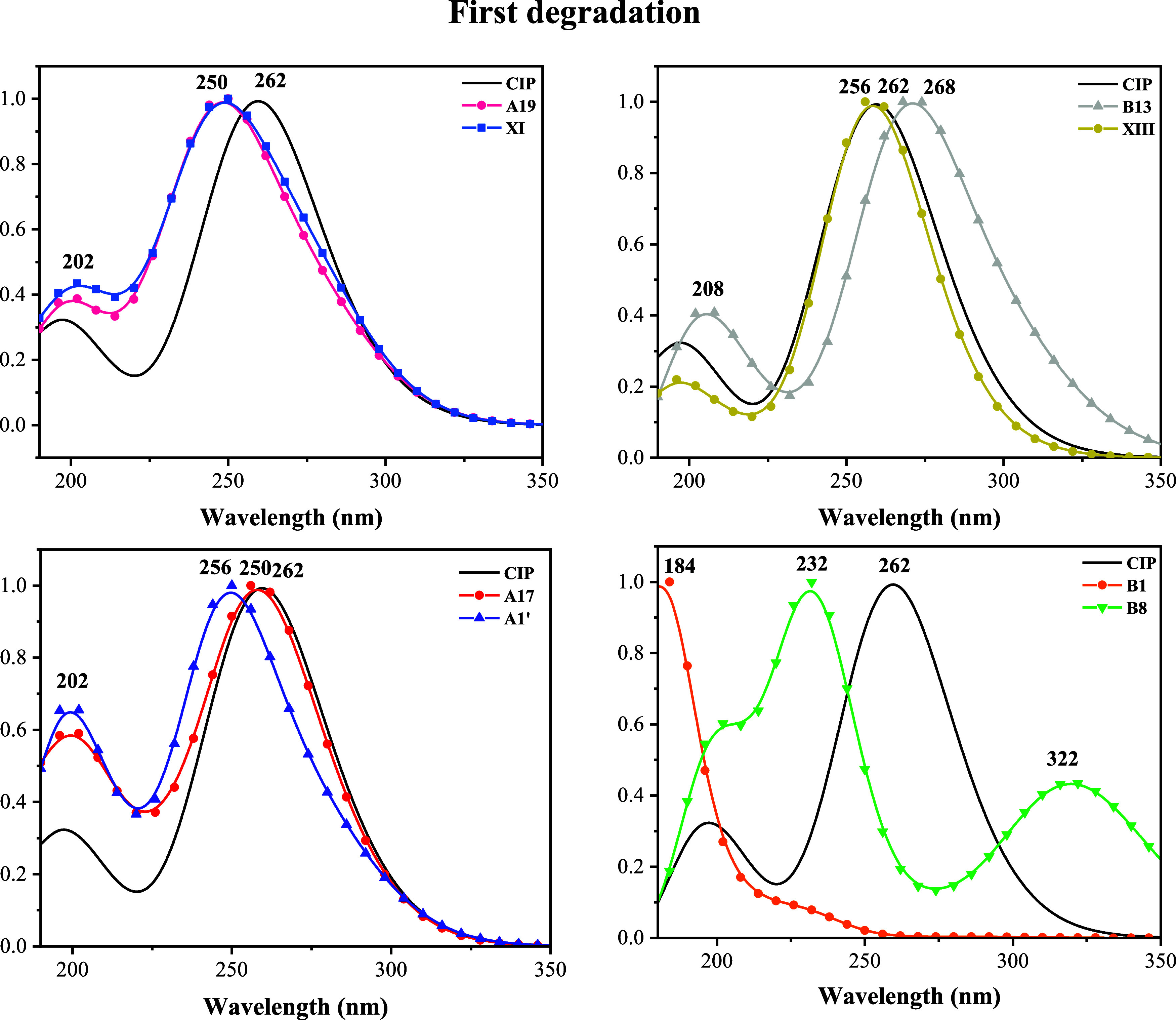
UV/visible spectra of CIP and the first degradation products. *Y*-axis notation is not included since *Y* axis values are the intensity and it is normalized.

Since the differences in maximum absorption signals are small, difficulties in identifying these products through experiments can be expected. Nevertheless, there are differences in the intensity of the second signal, which is useful for product identification. Figure 4 also shows two degradation products with very different spectra: B1 and B8, with maximum signals at 184 and 232 nm, respectively. B1 does not present a second signal, and there is a second signal at 322 nm for B8. The structural differences are the absence of an oxygen atom (B8) and the absence of double bonds in the six-membered ring (B1). These two products can be easily identified by UV/Visible spectroscopy.

To investigate the chemical reactivity of these compounds, we started by analyzing the electron transfer capacity. Figure 5 reports the FEDAM results for the first degradation products. We include Astaxanthin (ASTA) and ^•^OOH for comparison purposes. ASTA is a carotenoid that has been reported before as a good antioxidant compound.^37,38^ It is considered to be a good electron donor that prevents oxidative stress by donating electrons to free radicals. Figure 5 shows that most of the molecules are located at the same section of FEDAM, but there are some exceptions. B8 and B1 are worse electron acceptors than CIP (they have smaller values of A than CIP), but B8 is a better electron donor than CIP (lower value of I). Better electron acceptors than CIP are A17 and A1’. These last two compounds present additional hydroxyl or carboxyl groups in the pyrazine ring. When compared to ^•^OOH, both of them are better electron donors (all are down to the left), and their electron acceptor capacity is smaller than the electron acceptor capacity of ^•^OOH (lower value of A).

**Figure 5:**
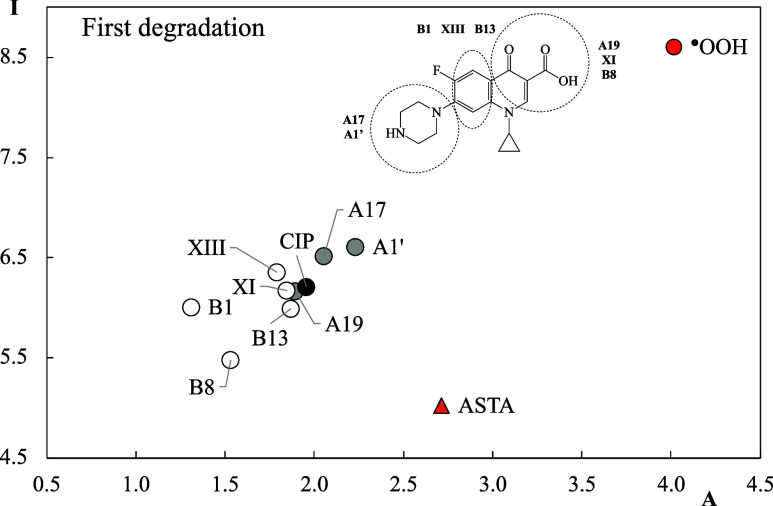
FEDAM shows the first degradation products. Values are reported in eV. ^•^OOH and ASTA are included for comparison.

The electron acceptor capacity is related to oxidative stress. ^•^OOH is a free radical that oxidizes molecules by accepting electrons from molecules that are oxidized. As the values in Figure 5 indicate, CIP is not as good an electron acceptor as ^•^OOH and cannot oxidize molecules easily; however, it is a good electron donor with respect to ^•^OOH. Since the donation of electrons reduces other molecules, degradation products and CIP may act as reducing rather than oxidizing agents, given electrons to other molecules such as ^•^OOH. Should this be the case, they can be considered antiradicals that could help to prevent oxidative stress, as is the case with ASTA, which is the best electron donor in Figure 5 (lowest I value). B8 is the best electron donor among compounds under study and could reduce other molecules more easily than CIP and the other first degradation products. In summary, the electron donor–acceptor properties of CIP differ slightly from those of the first degradation products, and all of these products could act as reducing agents when compared to ^•^OOH.

#### Second Degradation

In this investigation, the so-called *second degradation* refers to the reaction of the first degradation products with the ^•^OH free radical. These reactions have been previously described, and the labels we used are from ref 19. After the first degradation, the compounds in Figure 6 are formed. We included in Figure 6 the precursors of the second degradation products that were formed during the first degradation. Two of the degradation products have OH groups bonded to the piperazine ring (A20 and A18), whereas one molecule has a carbonyl group as well as a broken piperazine ring (A3). There are also three degradation products with OH groups bonded to the six-member rings (A20, B14, XIV). In this second degradation, there is also a product without oxygen atoms (B9).

**Figure 6:**
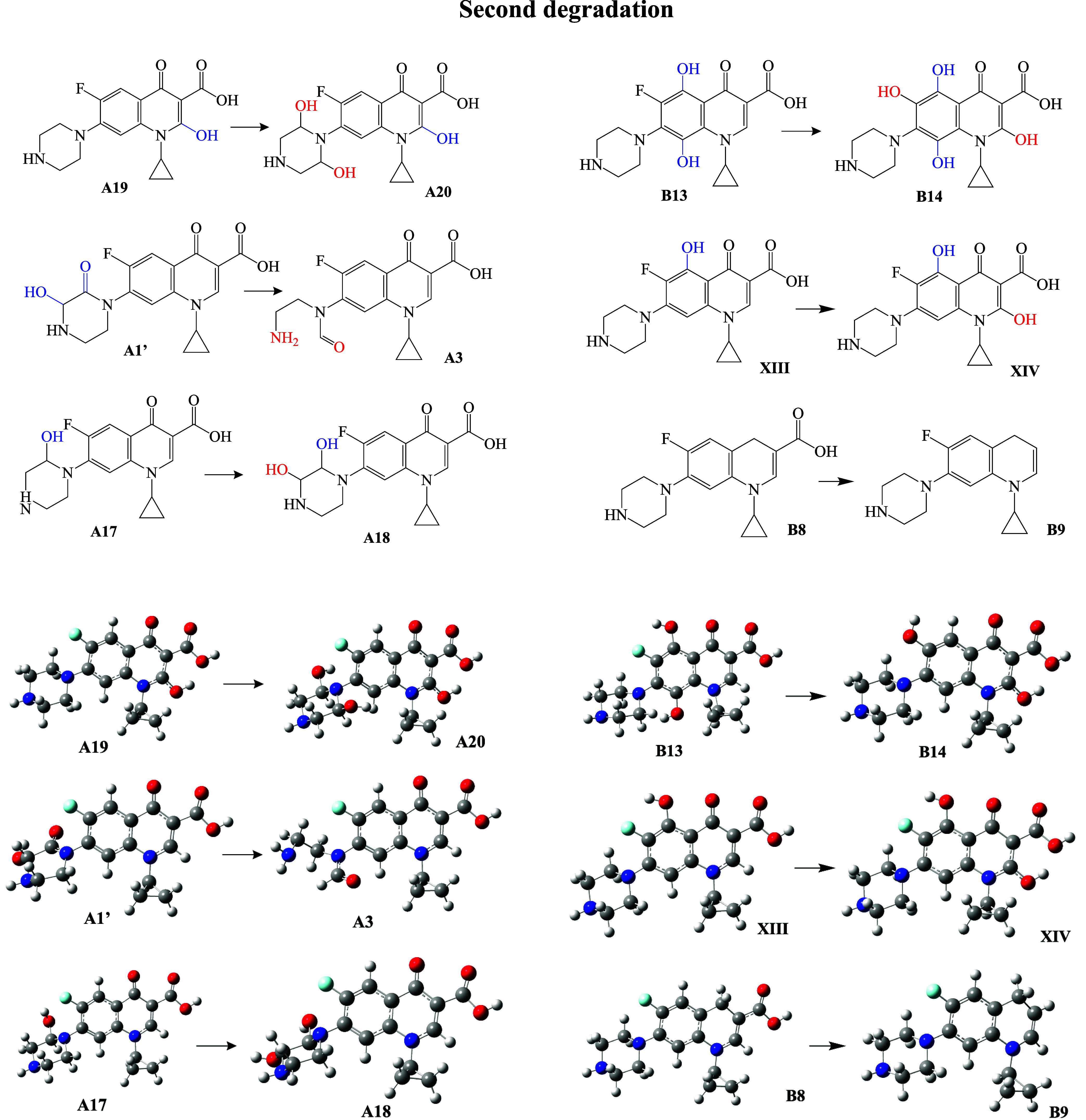
Schematic representation of the second degradation products. The reaction between first and second degradation products is shown. Optimized geometries are also included. Gray spheres represent carbon atoms, blue spheres are nitrogen atoms, oxygen atoms are in red, light blue is for fluorine atom, and white spheres are hydrogen atoms.

To provide more information that helps to identify the degradation products, we present the UV/Visible spectra in Figure 7. For A20, A3, A18, B14, and XIV, the maximum spectral values vary between 250 and 262 nm, and there is a less intense second signal. The second signal of A20 (214 nm) has a higher intensity than the other second signals. Under experimental conditions, these compounds are difficult to characterize using the maximum absorption peak of UV/Visible spectra, but the relative intensity of the second signal helps to identify the products. The greatest difference is found in the spectrum of B9, which has no oxygen atoms present. The maximum signal is at 214 nm, and there is also a new, less intense signal at 280 nm. Therefore, these degradation products are expected to be identifiable with UV/visible spectroscopy.

**Figure 7:**
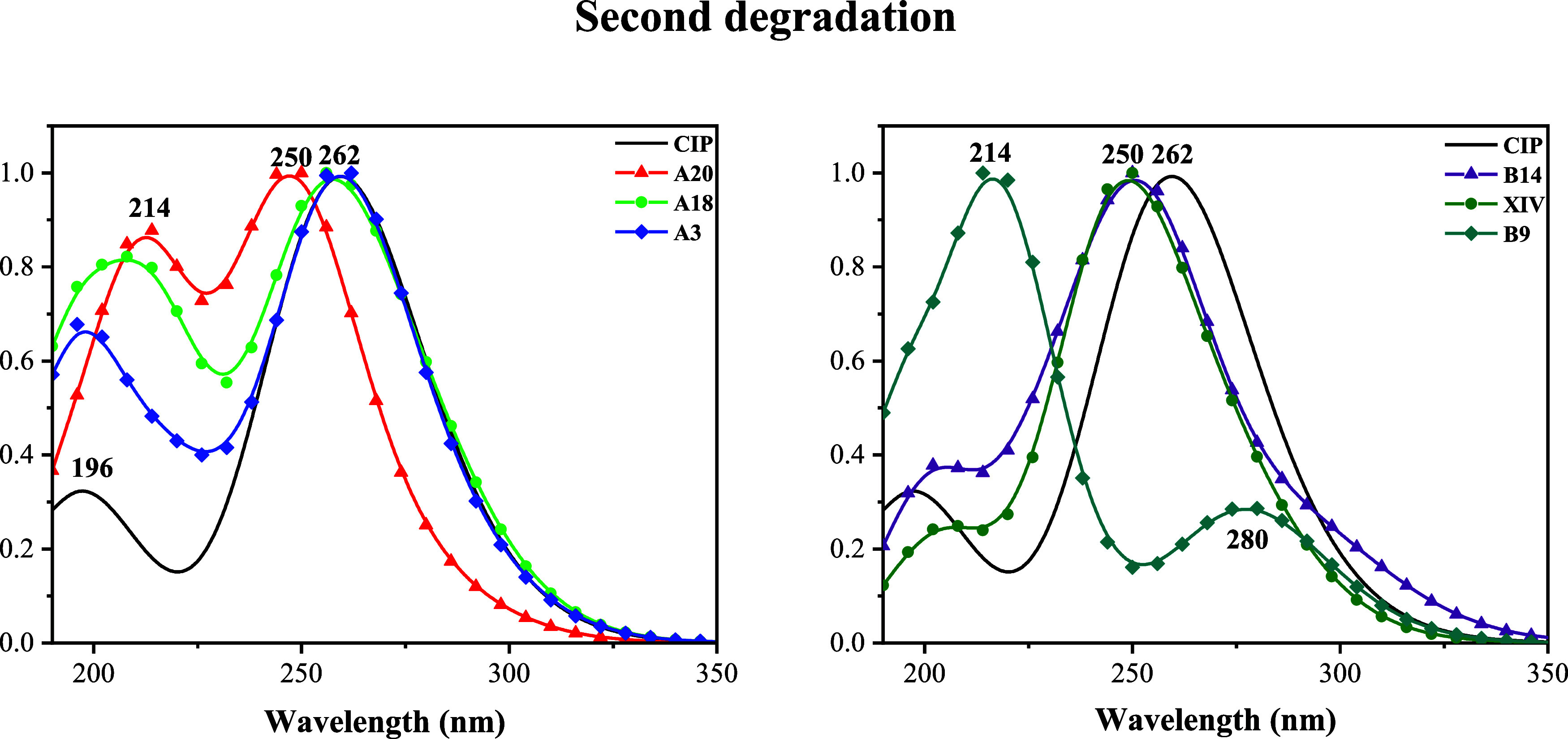
UV/visible spectra of CIP and the second degradation products. *Y*-axis notation is not included since *Y* axis values are the intensity, and it is normalized.

The electron donor–acceptor properties of the second degradation products are analyzed with FEDAM reported in Figure 8. B9 is the best electron donor, while A3, A18, and A20 are the best electron acceptors among the second degradation products that we investigate. B9 does not contain oxygen atoms. The differences in electron donor–acceptor properties with CIP are greater than the differences between CIP and the first degradation products. The degradation sites of A3, A18, and A20 are located in the pyrazine rings. This would suggest that modifications to the pyrazine ring make the molecules better electron acceptors. Comparing with ^•^OOH, all molecules are worse electron acceptors and cannot be expected to contribute to oxidative stress by oxidizing other molecules. B9 has a similar electron donor capacity to ASTA, a well-known antioxidant. It is possible to consider B9 as a good antioxidant that could help to prevent oxidative stress by donating electrons to free radicals.

**Figure 8:**
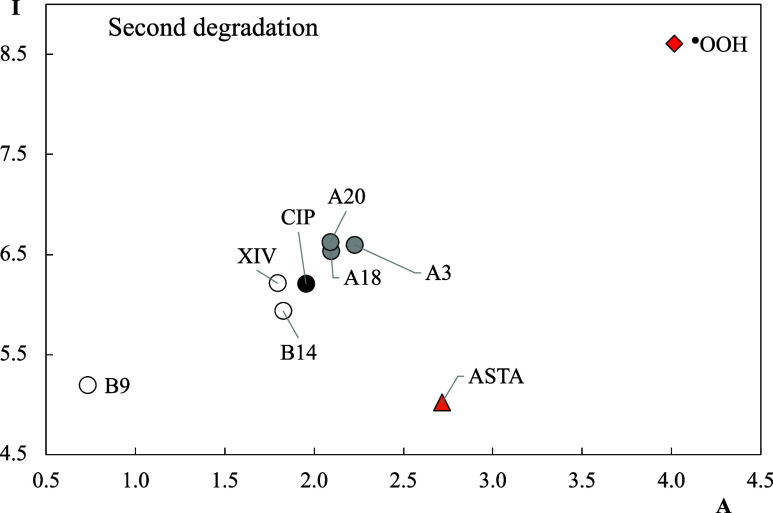
FEDAM of the second degradation products and CIP. Values are reported in eV. ^●^OOH and ASTA are included for comparison.

#### Further Degradations

From the second degradation product, further degradation may occur. During subsequent degradation reactions, many products may be formed, but in this investigation, we considered only those that were reported as products of the reaction with ^•^OH. In Figure 9, we report the degradation products coming from the second degradation products that we had considered. Some of them are modified in the pyrazine rings (A21 and A5). There is one compound without oxygen and fluorine atoms (B10), and there are others with an OH group added to different sites of CIP. B5 presents two carboxyl groups instead of the one that CIP has. UV/visible spectra are reported in Figure 10. All of the spectra present differences with respect to the spectrum of CIP. For A21, the maximum is at 244 nm, different from the 262 nm of CIP. The second signal of A5 (196 nm) shows higher intensity than the second signal of CIP. UV/Visible spectrum of B10 has a maximum at 226 nm, different from 262 nm for CIP. The spectrum of B6 is similar to the spectra of CIP. The presence of OH groups at different positions of B7 modifies the maximum signals and eliminates the second less intense signal. B5 presents the most different spectrum, with two signals at 202 and 292 nm. This degradation compound is the most different among all of the studied molecules. One of the six-membered rings has been removed, and two carboxyl groups are formed. These results are relevant because further degradation products can be individually identified through UV/Visible spectroscopy, which facilitates monitoring of the degradation reactions. After identification of the degradation products, it is important to know the electron transfer capacity of these degradation products.

**Figure 9:**
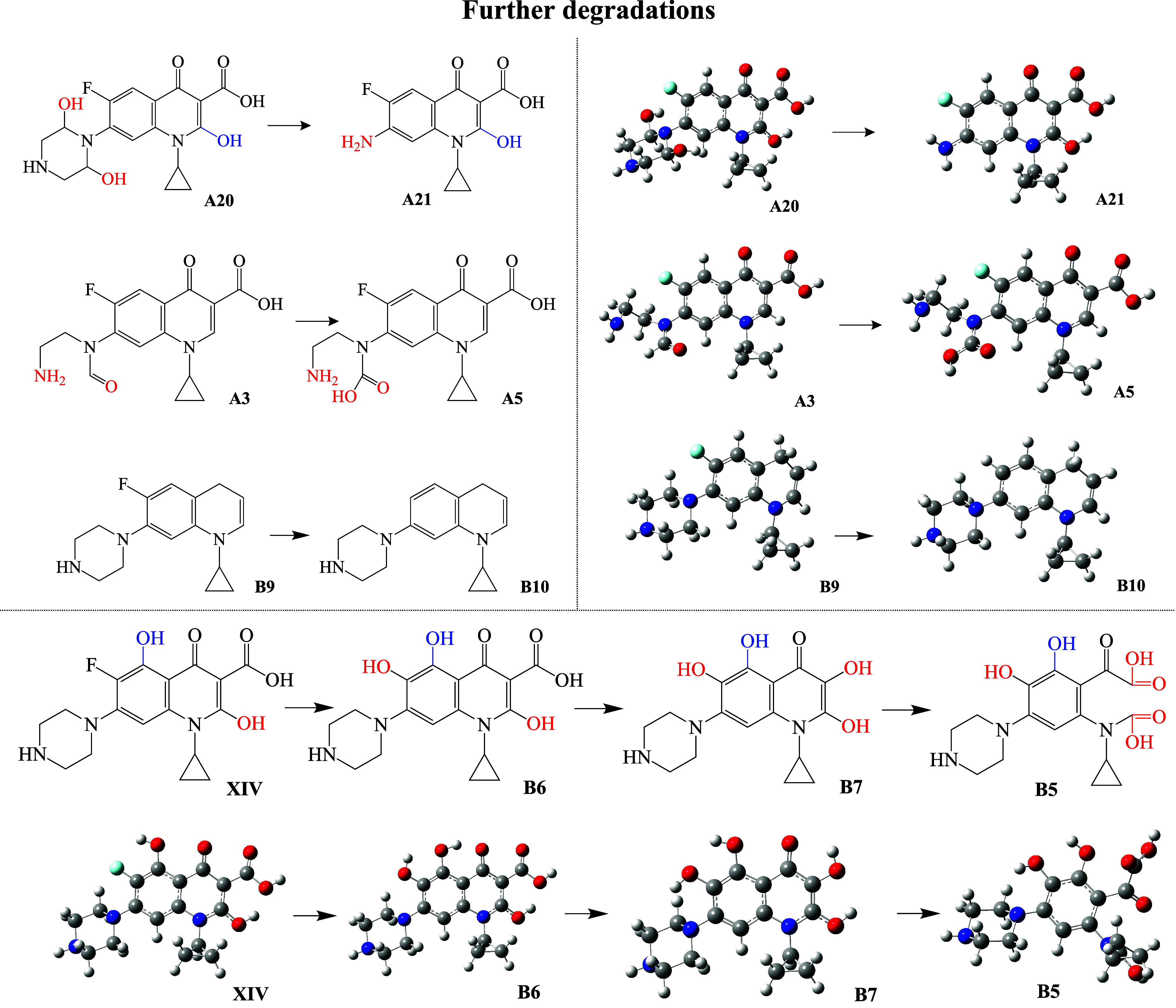
Schematic representation of further degradation products coming from second degradation products. Optimized geometries are also included. Gray spheres represent carbon atoms, blue spheres are nitrogen atoms, oxygen atoms are in red, light blue is for fluorine atom, and white spheres are hydrogen atoms.

**Figure 10:**
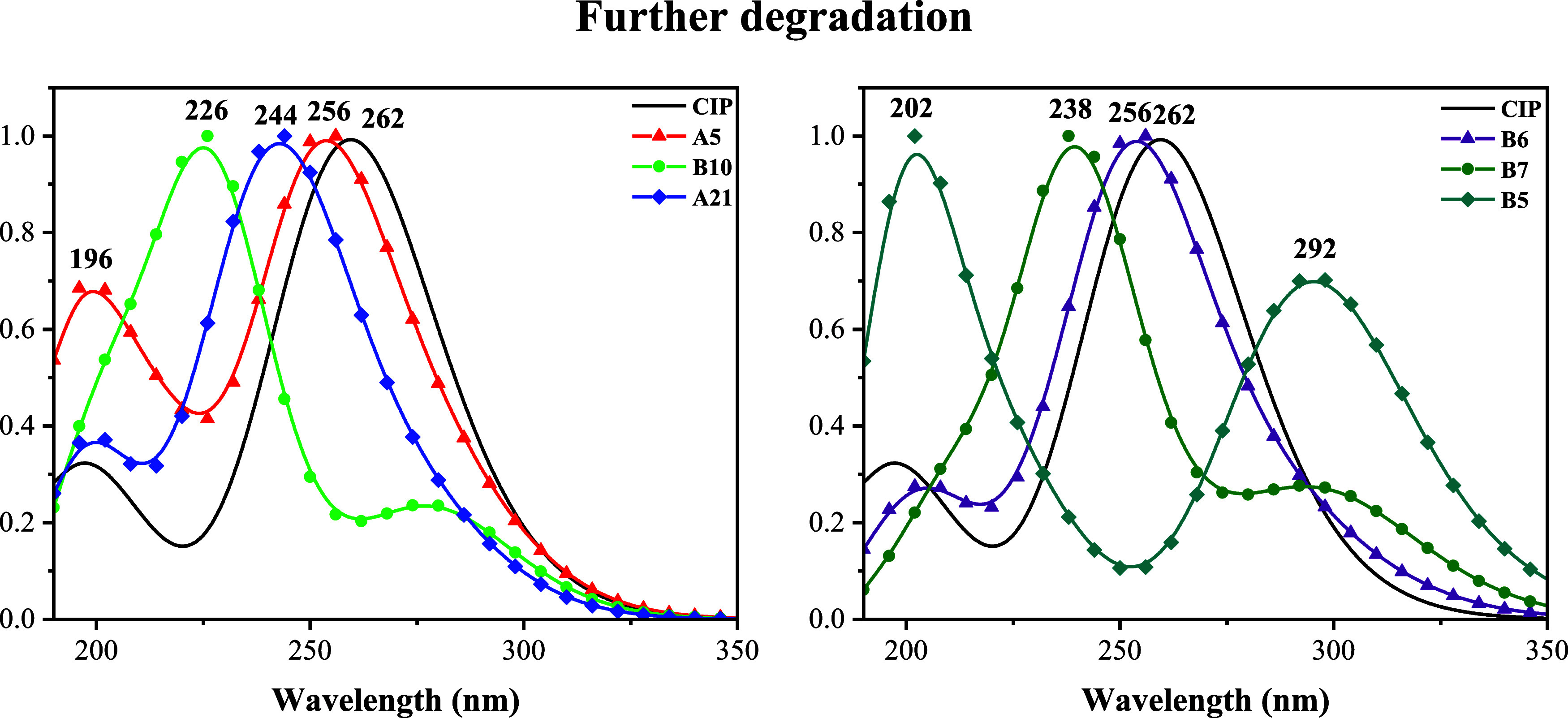
UV/visible spectra of CIP and the further degradation products. *Y*-axis notation is not included since *Y* axis values are the intensity and it is normalized.

The FEDAM in Figure 11 includes further degradation products. As can be seen, the differences with CIP are greater than those of the first and second degradation products. B10 is the best electron donor, with a value similar to that of ASTA. This indicates that B10 might donate electrons to free radicals such as ^•^OOH, preventing oxidative stress. B7, B6, and A21 are better electron donors than CIP, while A5 and B5 are better electron acceptors.

**Figure 11:**
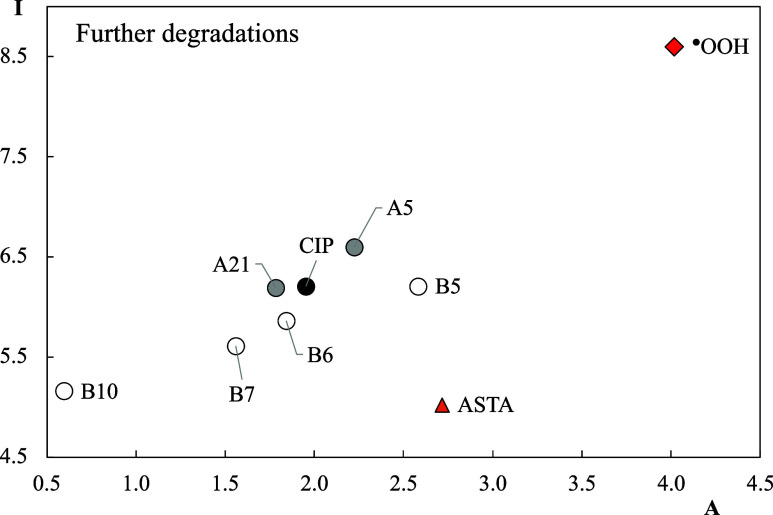
FEDAM of the further degradation products and CIP. Values are reported in eV. ^●^OOH and ASTA are included for comparison.

According to all of the results presented here, it is possible to say that as CIP degrades, the degradation products exhibit different electron donor–acceptor properties, which indicate different chemical reactivity. Degradation products such as B9 and B10 will donate electrons to free radicals, just as ASTA does; this would in turn help prevent oxidative stress and thus be beneficial. Further studies are necessary to corroborate this hypothesis.

#### Fukui Functions, Local Reactivity Analysis

To analyze the local reactivity and the nature of the interaction, the Fukui functions are presented in Tables 1 and 2. The values for all of the systems investigated here are reported in the Supporting Information (Table S1). In Tables 1 and 2, we include first degradation products and final degradation products (those that do not present further reactions with free radicals, according to ref 19). The maximum values of Fukui functions in each system are represented by gray spheres. These atoms can be considered to be reactive atoms. The number of reactive atoms can be related to the reactivity of the molecule. More reactive molecules will have more reactive atoms.

**Table 1:**
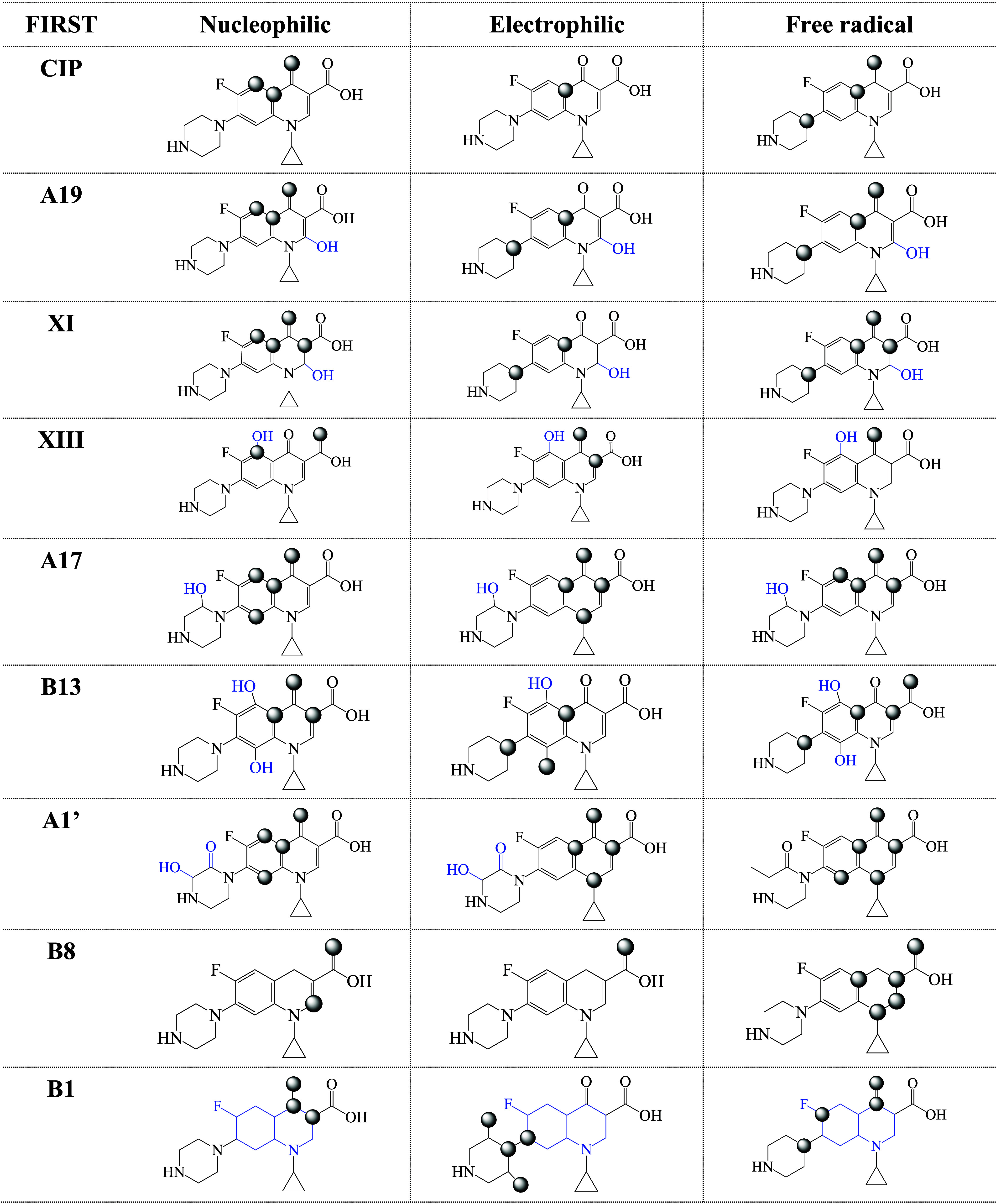
Fukui Function for CIP and the First Degradation Products. Gray Spheres Indicate the Atoms with Higher Values in Each Case. Nucleophilic, Electrophilic, and Free Radical Sites were Obtained with eqs 3 to 5 [Disp-formula eq5]

**Table 2:**
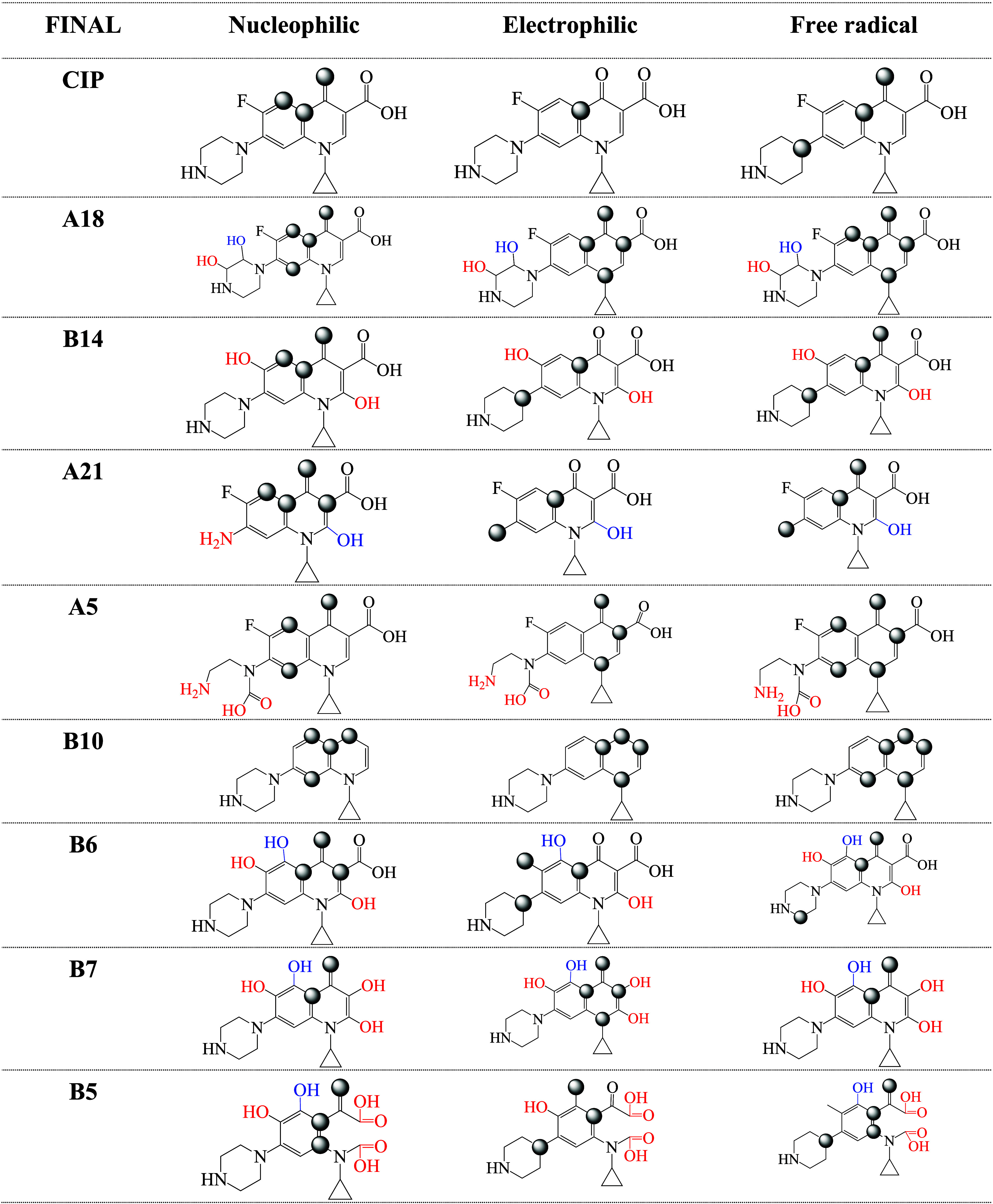
Fukui Function for CIP and the Final Degradation Products. Gray Spheres Indicate the Atoms with Higher Values in each Case. Nucleophilic, Electrophilic, and Free Radical Sites were Obtained with eqs 3 to 5

From the results reported in the tables, it is interesting to note that none of the maximum values of the Fukui functions are located on the F atom. Photocatalysis byproducts of ciprofloxacin reported before indicate that the F atom remains in the molecule during the degradation.^14^ This is in agreement with the results of the Fukui functions. For nucleophilic and electrophilic interactions, most Fukui functions are found on six-membered rings. A19, XI, B13, B1, B14, A21, B6, and B5 present electrophilic atoms on the pyrazine ring, whereas nucleophilic atoms on the pyrazine ring are not indicated by Fukui functions. The conclusion from the Fukui functions is that the degradation products and CIP have different reactive atoms; therefore, it is to be expected that the reactions with electrophiles, nucleophiles, and free radicals would be different in each case. For CIP, the Fukui functions indicate that the nucleophilic atoms are the oxygen of the carbonyl group and two of the carbons of the six-membered ring. There is only one electrophilic carbon atom in a six-membered ring, and free radical attack can occur at the carbonyl group, at the pyrazine ring, or at an atom of the double bonds in the six-membered ring. In summary, CIP has three reactive atoms for nucleophilic attack, one for electrophilic interactions, and three for free radical attack. Comparing the values for molecules in Tables 1 and 2, the degradation products present more reactive atoms than CIP for any interaction, meaning nucleophilic, electrophilic, or free radicals. The conclusion that emerges from these values is that the degradation products are more reactive than CIP, which could have consequences for health and the environment, in turn representing a potential hazard.

#### Experimental Photocatalytic Degradation of CIP

ZnO NW films were characterized prior to photocatalytic evaluation. Figure 12a shows FESEM images with the vertically aligned ZnO NW, which presented an average size of 4.5 μm length and 60–70 nm diameter. TEM micrograph (Figure 12b) reveals the presence of Au catalyst on the top of each wire as a product of the VLS growth. The XRD pattern of Figure 12c shows a prominent peak corresponding to the (002) plane, indicating the highly oriented growth of ZnO along the *c*-axis. The pattern was indexed with the characteristic hexagonal wurtzite structure (ICDD 01–070–02551). Additionally, PL spectra and XPS measurements were acquired (see Figures S1 and S2). The generation of ^•^OH radicals by ZnO films was confirmed by a terephthalic acid (TA) test. Figure 12d presents the fluorescent signal at 427 nm of 2-hydroterephtalic acid (HTA) that is formed when ^•^OH radicals react with TA under UV illumination. This is evidence of the importance of ^•^OH radicals as reactive species in the photocatalytic process.^34,49^ Furthermore, a superoxide radical (O_2_^•‑^) scavenger test was performed by adding benzoquinone (1 mM) to the CIP solution. The result, shown in Figure S3, revealed that the photocatalytic activity decreased by 27.3% (from 87.1 to 63.3%). This test confirms that the species that contributes most to photocatalytic degradation is the hydroxyl radical.

**Figure 12:**
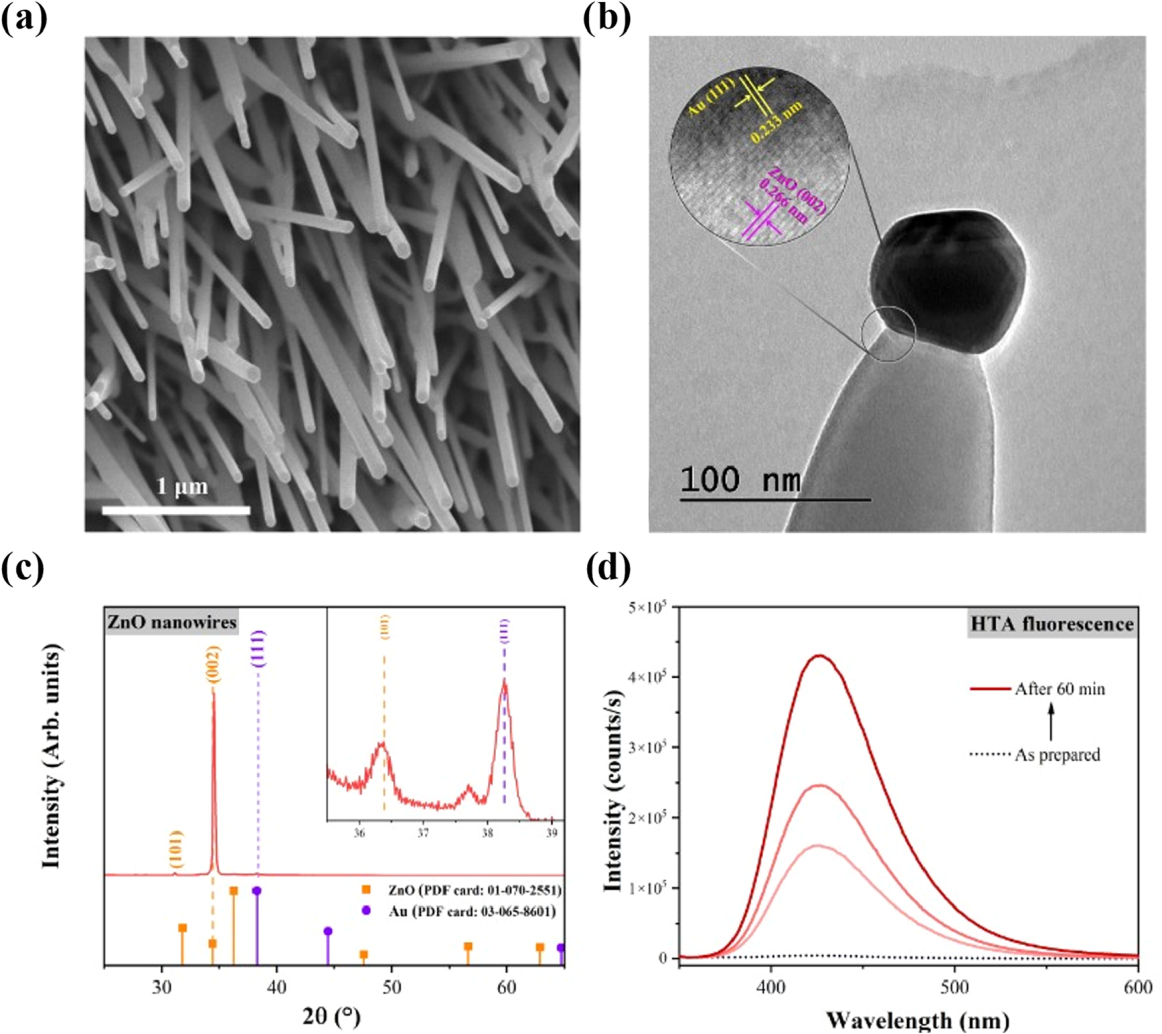
(a) FESEM image showing the vertically aligned ZnO NW films. (b) TEM image of a single nanowire showing the presence of Au on the top. (c) XRD pattern of ZnO NW film, the inset shows the presence of Au catalyst. (d) Fluorescence spectra of HTA as a reaction product of TA with ^•^OH radicals.

Photocatalytic degradation of CIP was monitored over 3 h under UV irradiation and vigorous stirring. Initial photolysis (control) experiments were conducted to determine the effect of UV irradiation. Figure 13a,b presents the evolution of CIP’s absorbance spectra over 3 h of UV irradiation for control and ZnO NW samples, respectively. A slight decrease in the main absorbance peak centered at 276 nm after 3 h of UV exposure was observed without a photocatalyst (Figure 13a). This diminution indicates that CIP suffers a slight instability at initial UV irradiation, and it gradually stabilizes. In contrast, in the presence of ZnO NW photocatalyst, the spectra changed significantly during the reaction. Peaks located at 276 and 335 nm decreased while the peak at 207 nm increased, indicating a transformation of the original CIP molecule. Figure 13c illustrates the change in the relative concentration (*C*/*C*_0_) of CIP’s main absorbance peak (at 276 nm) measured by UV–vis spectroscopy. The 3 h photocatalytic reaction showed 89% of CIP degradation, indicating the effectiveness of the ZnO NW photocatalyst, contrasting with the 24% reached by UV photolysis in the control sample. To confirm this result, HPLC measurements were performed, taking aliquots of the solution every 30 min of reaction. The results are reported in Figure 13d. The *C*/*C*_O_ ratio reached 96.3% of CIP degradation. As can be seen, both techniques showed the same behavior, validating the effectiveness of ZnO NWs as photocatalysts and validating the UV–vis method.

**Figure 13:**
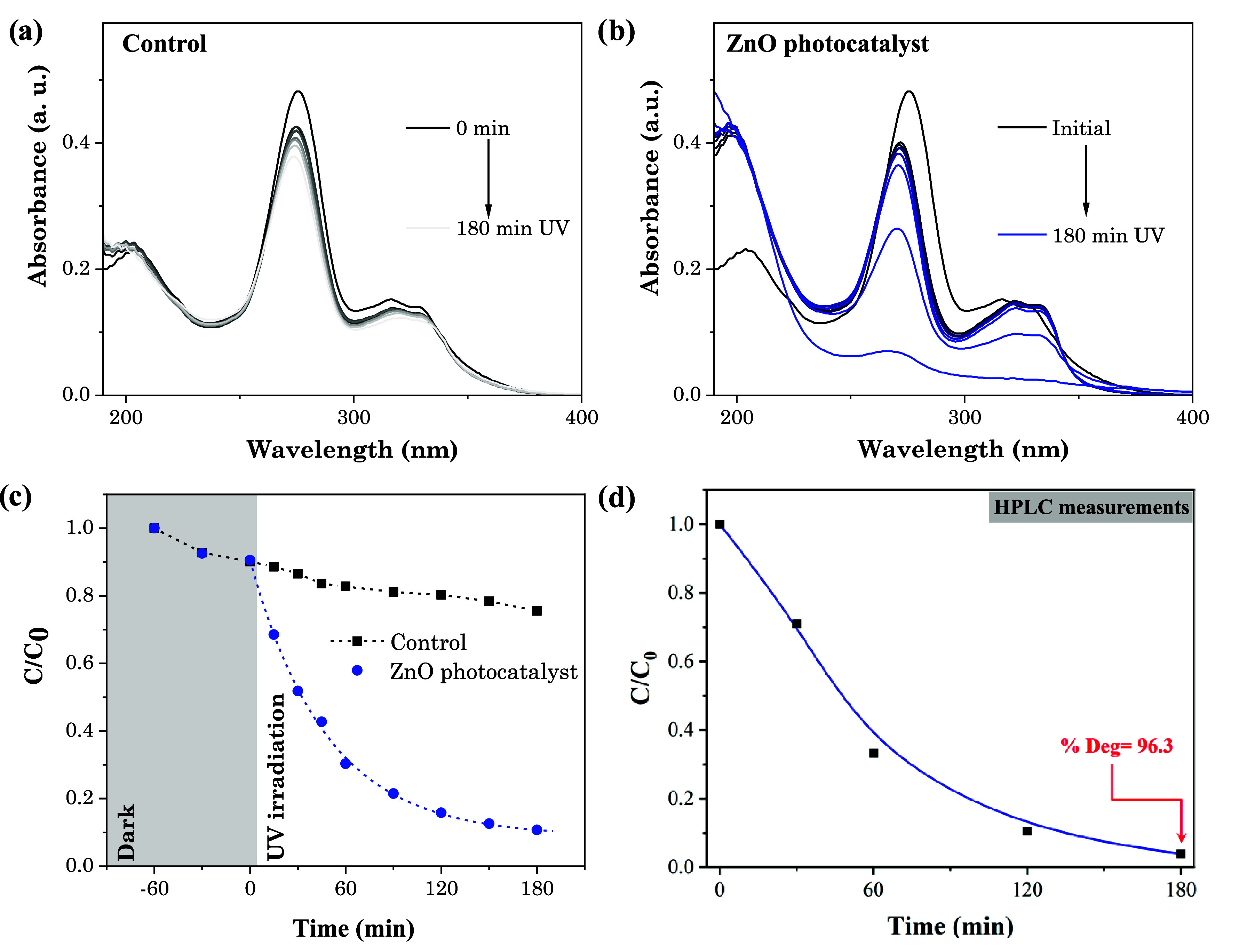
Evolution of UV absorbance spectra for (a) control tests and (b) ZnO NW photocatalytic material. (c) Change of CIP’s relative concentration as a function of time, determined by UV–vis spectroscopy. (d) Relative concentration of CIP measured by HPLC as a function of the reaction time.

#### Theory and Experiment

To elucidate the possible CIP degradation products from UV–vis results, spectral deconvolution analysis was performed on spectra acquired at 0 and 180 min (Figure 14). Gaussian peak fitting was applied to resolve overlapping spectral features, enabling identification of the main contributing species (degradation intermediates) through their distinct peak positions and relative intensities. This quantitative approach revealed specific signatures in the initial CIP spectrum (as prepared) and also the changes in spectrum intensity over the 180 min interval, providing insights into the temporal dynamics of chemical or structural transformations of CIP.

**Figure 14:**
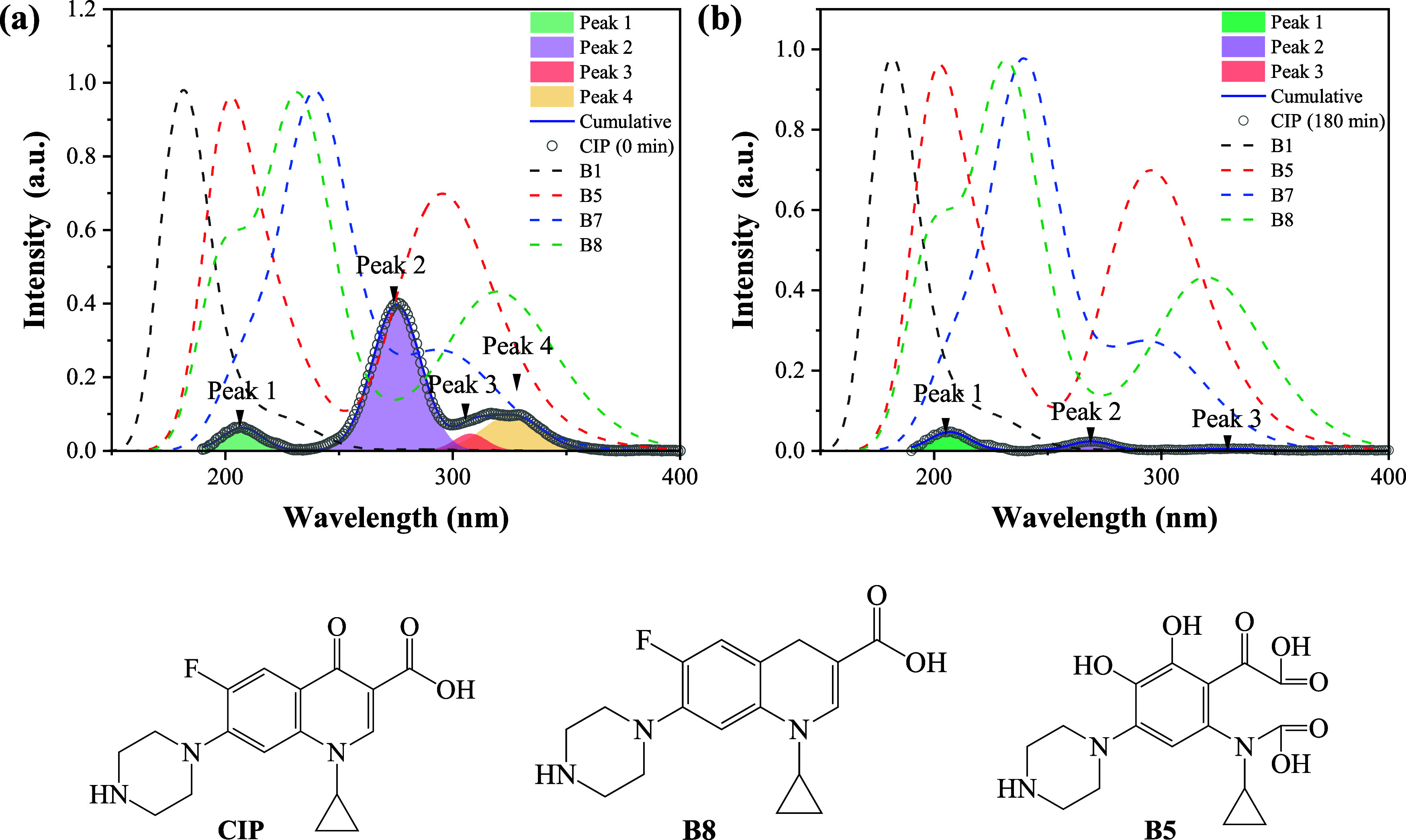
Deconvolution analysis of CIP spectra at initial conditions (0 min) (a) and after reaction for 180 min of reaction (b). Theoretical results (dashed lines) are also included for comparison. Molecular structures of CIP and degradation products are included for quick reference.

The deconvolution analysis for the initial CIP spectrum, Figure 14a, shows four main peaks centered at 203.1, 275.8, 321.1, and 335.7 nm, whereas for the spectrum after 180 min of UV light irradiation (Figure 14b), the spectrum exhibited only three peaks (206.8, 273.5, and 329.0 nm) with less intensity, because of the photocatalytic degradation process. Additionally, in both figures, the theoretically calculated absorbance spectra B1, B5, B7, and B8 proposed as intermediates (dashed lines) are included. It is possible to observe that the spectra of B5 and B8 are quite similar to that observed in the deconvoluted spectrum of CIP, which suggests their presence during the degradation reaction.

B5 and B8 are degradation products formed experimentally during photocatalytic degradation. Once the possible degradation products have been identified, it is important to understand their chemical reactivity based on theoretical data. B8 is formed during the first degradation process. The difference between CIP and B8 lies in a carbonyl group in one of the six rings. B5 is formed during subsequent degradations and is different from CIP, as it has two carboxyl groups instead of the one that CIP has. B8 is a better electron donor than CIP (see Figure 5). The electron donor capacity of B5 is similar to the electron donor capacity of CIP, but it is a better electron acceptor than CIP (see Figure 11). B5 is almost as good electron acceptor as ASTA. We are considering in this investigation that the electron donor capacity might be related to the capacity to prevent oxidative stress, since these molecules donate electrons to free radicals. Should this be the case, B8 is a better reducing agent and therefore, a better antioxidant than CIP. B5 and CIP have similar electron donor capacity, so both will have similar capacity as reducing agents. B5 is a good electron acceptor, but is not as good as ^•^OOH. We do not consider that this molecule will oxidize other molecules as •OOH does. Data from Tables 1 and 2 indicate that the number of atoms for a nucleophilic interaction is similar (three) for CIP and B5, while B8 has only two atoms for nucleophilic interactions. B5 has more atoms susceptible to electrophilic attack than CIP, while B8 and CIP only have one atomic site for the electrophilic interaction. CIP presents three atoms susceptible to free radical attack, while B8 has five and B5 has four. This can be interpreted as more reactive. All differences in the Fukui functions indicate different chemical characteristics. Molecules with more atoms susceptible to electrophilic, nucleophilic, or free radical attack are more reactive. B8 and B5 are more reactive than CIP, and this could have consequences to health. Further studies are needed to determine the potential hazards of these degradation products.

## Conclusions

According to all the results presented here, it is possible to say that ZnO NW films are effective photocatalysts to degrade CIP. The experimental UV–vis spectra of CIP’s degradation products were correlated with those theoretically calculated. The degradation products of CIP exhibit different electron donor–acceptor properties, which indicate different chemical reactivity. The possibility that degradation products, such as B8, B9, and B10, donate electrons to free radicals could help to prevent oxidative stress, as ASTA does. Further studies are necessary to corroborate this hypothesis.

Fukui functions indicate reactive atoms at the local level. Nucleophilic, electrophilic, and free radical attacks were carried out on different atoms. All differences in the Fukui functions indicate different chemical characteristics. Molecules with more atoms susceptible to electrophilic, nucleophilic, or free radical attacks are, in principle, more reactive. Most degradation products have more reactive atoms than CIP for any interaction, i.e., nucleophilic, electrophilic, or free radical. The conclusion drawn from these values is that the degradation products are more reactive than CIP itself, which could have consequences for health and the environment, in addition to presenting potential risks.

B5 and B8 are formed during the photocatalytic experiment. The chemical characteristics of these two molecules indicate the differences from those of CIP. B8 is a better electron donor and could help to prevent oxidative stress by donating electrons to free radicals. B5 is a better electron acceptor, but it is not as good as ASTA and ^•^OOH, and therefore, it is not expected that this molecule will oxidize other molecules. B5 and B8 both have more reactive atoms than CIP, and this could represent potential hazards. For a safe photocatalytic degradation process, it is advisable to achieve complete mineralization. In summary, the degradation products of CIP have different chemical characteristics. This could represent an increase in the potential hazards of the degradation products due to the greater number of reactive atoms. Further studies are necessary to corroborate these findings.

## Supplementary Material



## Notes

Details of experimental methods and results; characterization data of ZnO nanowires; Fukui functions; and Cartesian coordinates of optimized structures (PDF)
