# Supplementary material for: Chemical Characteristics of Ciprofloxacin and Its Degradation Products: On the Road to Understanding Its Potential Hazards
Source: ACS Omega. 2025 Nov 27;10(48):58656–70. doi: 10.1021/acsomega.5c06831 (PMC12771441; doi:10.1021/acsomega.5c06831)
Supplement: Supplementary file 1 [file ao5c06831_si_001.pdf]

Chemical characteristics of ciprofloxacin and its degradation products: on the road to understand its potential hazards

Alexis Caballero, Emiliano Perez-Sanchez, Amauri Serrano-Lázaro, Monserrat Bizarro, Ana Martínez*

Departamento de Materiales de Baja Dimensionalidad. Instituto de Investigaciones en Materiales, Universidad Nacional Autónoma de México. CDMX 04510. México.

Experimental results

ZnO NW films were grown by the vapor-liquid-solid technique. Briefly, a seed layer of aluminum doped ZnO (AZO) was deposited by sputtering on quartz substrates. Then, a 4 nm gold layer was sputtered using a Cressington Scientific DC sputter coater. These pre-treated substrates were introduced into a tubular oven next to a 1:1 mixture of ZnO powder (Sigma-Aldrich, 99%) and graphite powder (Sigma-Aldrich, 99%) that served as precursors. ZnO NWs were grown at 900 °C for one hour, under a constant argon (Infra 99.99%) flow rate of 3.3 L/min. ZnO NW films were characterized by X-ray diffraction using a Rigaku Ultima IV with a Cu K_{α} radiation of 1.5418 Å in grazing angle mode. The morphology of the films was analyzed with micrographs acquired with a JEOL 7600F field-emission scanning electron microscope (FESEM). Transmission electron micrographs were also acquired with a JEOL ARM 200F microscope. Room-temperature photoluminescence (PL) measurements were performed using a Kimmon He-Cd laser (IK Series) with excitation wavelength of 325 nm and 25 mW power. X-ray photoelectron spectroscopy was used to analyze the chemical oxidation states of the elements with a PHI 5000 VersaProbe II device.

Room temperature photoluminescence measurements were performed, indicating a strong emission centered at 505 nm. The deconvolution analysis revealed the presence of point defects in the ZnO lattice (presented in Figure S1). XPS analysis was performed to elucidate surface chemistry characteristics of ZnO nanowires. Figure S2a exhibits the survey spectra of sample where Zn, O, and Au elements are highlighted. The high-resolution of O 1s core

* Corresponding author: martina@unam.mx

level, Figure S3b, exhibits a main peak at 529.25 eV associated to O_2^- state within the ZnO crystal. The strong M-O signal in the O 1s also indicates a high surface-to-volume ratio, a main characteristic of 1D nanostructures. The Zn 2p spectrum (Figure S2c) shows a doublet with binding energies of 1043.50 and 1020.45 eV, corresponding to Zn $2p_{1/2}$ and Zn $2p_{3/2}$, respectively. The difference of ~23 eV between peaks confirms the presence of Zn^{2+} oxidation state of ZnO nanowires. In the case of Au 4f spectrum, its presence is associated with Au nanoparticles observed at the tip of ZnO nanowires due to the VLS growth mechanism (as observed in TEM micrographs). Peaks at 82.2 and 85.95 eV (ΔBE= 3.7 eV) are linked to Au $4f_{7/2}$ and Au $4f_{5/2}$ core levels, whereas peaks found at 85.25 and 90.24 eV are attributed to Zn 3p3/2 and Zn 3p1/2 [Ref 1 and 2]. The overlap of the Au4f and Zn 3p signals occurs when there is intimate contact or formation of an interface between ZnO and Au, especially when nanowire growth is catalyzed by Au nanoparticles [Ref 2].

Figure S1. Room temperature photoluminescence spectrum of ZnO NW films and its deconvolution showing the band-to-band transition (NBE) and the contribution of the different point defects that are naturally formed in the structure: interstitial oxygen (O_i), oxygen vacancies (V_O) and zinc vacancies (V_{Zn}).

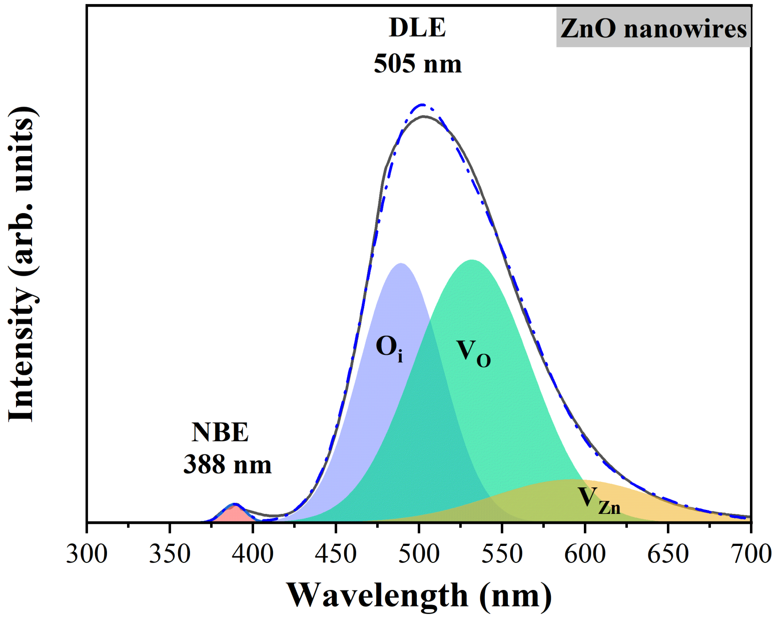

Figure S2. XPS analysis of ZnO NW film, a) survey spectrum. High resolution spectra: b) O 1s orbital, c) Zn 2p orbital, d) Au 4f orbital.

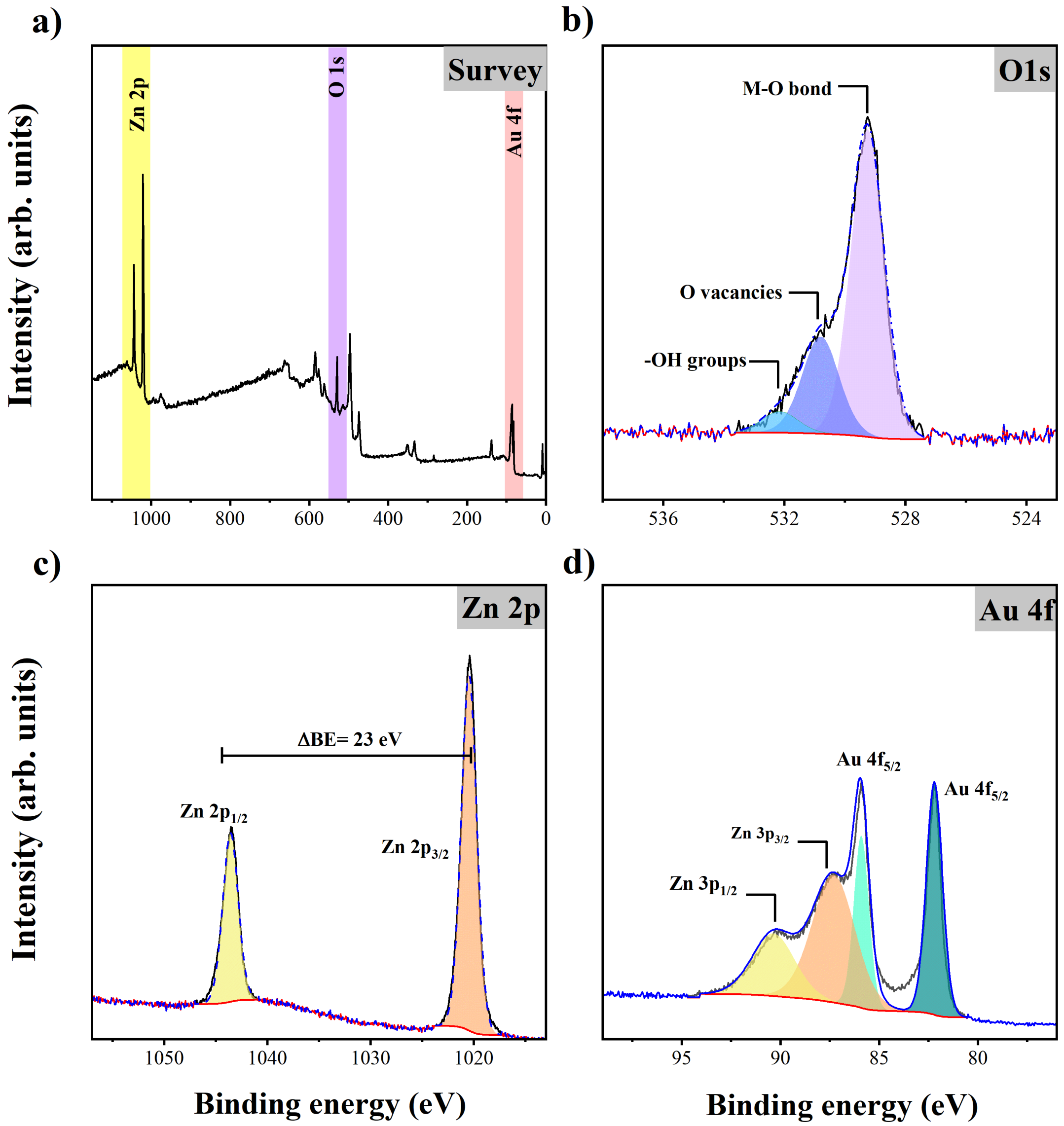

Figure S3. Superoxide radical scavenger test using benzoquinone. The presence of benzoquinone produced a 27% decrease in the total photocatalytic activity of ZnO NW. This indicates that $O_2^{\bullet-}$ are important species but not the predominant ones.

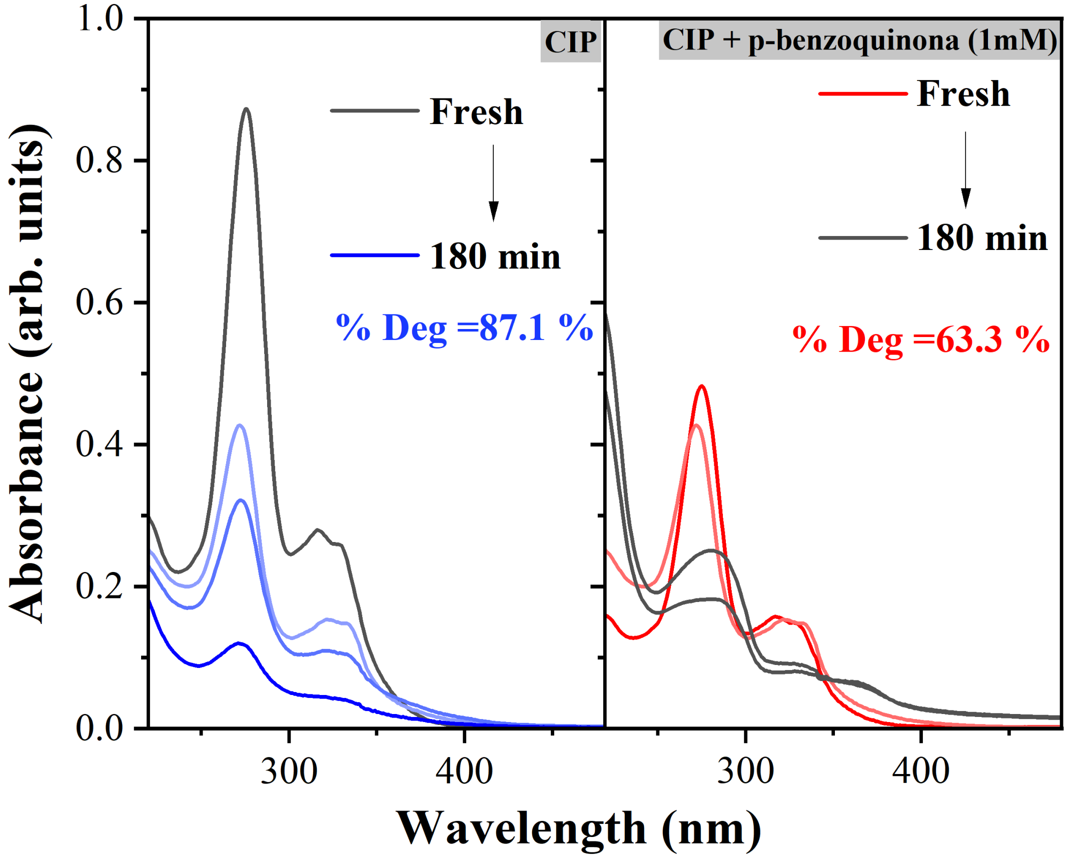

Ref. 1. Panda, S.A.; Choudhary, S.; Barala, S.; Hazra, A.; Jena, S.K.; Gangopadhyay, S. Surface energy and stress driven growth of extremely long and high-density ZnO nanowires using a thermal step-oxidation process. *RSC Advances*, **2024**, *14*, 28086-28097.

Ref. 2. Perumal, V.; Hashim, U.; Gopinath, S.C.B.; Prasad, H.; Wei-Wen, L.; Balakrishnan, S.R.; Vijayakumar, T.; Rahim, R.A. Characterization of Gold-Sputtered Zinc Oxide Nanorods-a Potential Hybrid Material. *Nanoscale Research Letters* **2016,** 11:31

Table S1. Cartesian coordinates, atomic charges and Fukui functions of the optimized geometries.

Ciprofloxacin

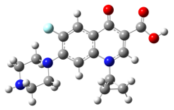

Standard orientation:

Center Number	Atomic Number	Atomic Type	Coordinates (Angstroms) X	Y	Z
1	9	0	-2.105083	-2.788196	-0.603909
2	8	0	2.851723	-2.644557	-0.284592
3	8	0	5.417842	-1.600480	0.140597
4	8	0	5.506325	0.610260	0.415482
5	7	0	1.567694	1.209968	0.204421
6	7	0	-3.070693	-0.251442	-0.187412
7	7	0	-5.697694	0.595128	0.583887
8	6	0	1.126668	2.581074	0.390000
9	6	0	1.893761	3.683040	-0.261513
10	6	0	0.533345	3.306021	-0.775367
11	6	0	0.623091	0.195757	0.024155
12	6	0	2.864937	0.911143	0.270379
13	6	0	1.065476	-1.119657	-0.138390
14	6	0	-0.745102	0.495871	0.032347
15	6	0	-1.696392	-0.498390	-0.145575
16	6	0	-3.844901	-0.966238	0.849067
17	6	0	-3.463348	1.159403	-0.224789
18	6	0	3.379716	-0.357721	0.124891
19	6	0	2.489182	-1.472450	-0.114699
20	6	0	-5.329141	-0.821601	0.591600
21	6	0	-4.952954	1.273522	-0.478601
22	6	0	0.108839	-2.126216	-0.333305
23	6	0	-1.214006	-1.811330	-0.345814
24	6	0	4.832182	-0.540387	0.220477
25	1	0	0.698949	2.758170	1.368250
26	1	0	2.742010	3.410559	-0.874544
27	1	0	2.001780	4.596965	0.304320
28	1	0	-0.305638	3.956401	-0.571191
29	1	0	0.496239	2.766839	-1.712619
30	1	0	3.519416	1.751696	0.452487
31	1	0	-1.063941	1.513001	0.192502
32	1	0	-3.598389	-0.544734	1.833196
33	1	0	-3.576315	-2.018957	0.850888
34	1	0	-2.921038	1.657081	-1.029390
35	1	0	-3.215058	1.659520	0.722020
36	1	0	-5.572411	-1.311480	-0.361246
37	1	0	-5.873489	-1.334161	1.384761
38	1	0	-5.225833	2.328578	-0.495198
39	1	0	-5.176212	0.847991	-1.466449
40	1	0	0.422267	-3.149409	-0.495339

41	1	0	-6.687280	0.663535	0.367962
42	1	0	6.450304	0.393766	0.477406

CIP		q(A)	q(A+1)	q(A-1)	fk+	fk-	fk0
1	F	-0.32737	-0.296068	-0.348087	0.02	0.03	0.03
2	O	-0.700363	-0.643158	-0.876149	0.18	0.06	0.12
3	O	-0.605596	-0.595074	-0.629591	0.02	0.01	0.02
4	O	-0.489145	-0.48554	-0.496985	0.01	0.00	0.01
5	N	0.38458	0.42078	0.314234	0.07	0.04	0.05
6	N	-0.083423	0.213285	-0.153525	0.07	0.30	0.18
7	N	-0.384655	-0.377366	-0.384959	0.00	0.01	0.00
8	C	-0.359125	-0.383372	-0.303762	0.06	0.02	0.04
9	C	-0.310247	-0.304456	-0.309982	0.00	0.01	0.00
10	C	-0.260912	-0.258148	-0.272559	0.01	0.00	0.01
11	C	0.055101	0.062161	0.047571	0.01	0.01	0.01
12	C	-0.626265	-0.630206	-0.698055	0.07	0.00	0.03
13	C	0.422564	0.546372	0.311644	0.11	0.12	0.12
14	C	-0.370906	-0.340831	-0.450087	0.08	0.03	0.05
15	C	0.342933	0.291351	0.291866	0.05	0.05	0.00
16	C	-0.27943	-0.337832	-0.215854	0.06	0.06	0.06
17	C	-0.071942	-0.110596	-0.054437	0.02	0.04	0.03
18	C	0.337278	0.377132	0.272939	0.06	0.04	0.05
19	C	0.157252	0.144324	0.117994	0.04	0.01	0.01
20	C	-0.293328	-0.287076	-0.309886	0.02	0.01	0.01
21	C	-0.523788	-0.477907	-0.536437	0.01	0.05	0.03
22	C	-0.345231	-0.307136	-0.483752	0.14	0.04	0.09
23	C	-0.241099	-0.187942	-0.18975	0.05	0.05	0.00
24	C	0.888011	0.88922	0.880344	0.01	0.00	0.00
25	H	0.208166	0.214522	0.186709	0.02	0.01	0.01
26	H	0.177158	0.180557	0.169216	0.01	0.00	0.01
27	H	0.172202	0.175311	0.166275	0.01	0.00	0.00
28	H	0.181266	0.184486	0.174197	0.01	0.00	0.01
29	H	0.189636	0.193132	0.182318	0.01	0.00	0.01
30	H	0.214766	0.224207	0.185423	0.03	0.01	0.02
31	H	0.215506	0.251153	0.185245	0.03	0.04	0.03
32	H	0.177712	0.251101	0.164503	0.01	0.07	0.04
33	H	0.189765	0.226058	0.181268	0.01	0.04	0.02
34	H	0.192338	0.231829	0.182982	0.01	0.04	0.02
35	H	0.193092	0.264814	0.180646	0.01	0.07	0.04
36	H	0.164715	0.185389	0.160039	0.00	0.02	0.01
37	H	0.182247	0.198919	0.17779	0.00	0.02	0.01
38	H	0.185582	0.202675	0.181102	0.00	0.02	0.01
39	H	0.167422	0.188299	0.162772	0.00	0.02	0.01
40	H	0.200557	0.219423	0.172751	0.03	0.02	0.02
41	H	0.283924	0.294968	0.281043	0.00	0.01	0.01
42	H	0.389053	0.391242	0.382987	0.01	0.00	0.00

A19

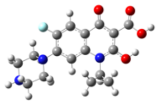

Standard orientation:

Center Number	Atomic Number	Atomic Type	Coordinates (Angstroms) X	Y	Z
1	9	0	-2.318995	-2.778874	-0.738580
2	8	0	2.641454	-2.706149	-0.527804
3	8	0	5.087961	-1.950213	0.430919
4	8	0	5.448084	0.241794	0.461312
5	7	0	1.441170	1.139510	0.122343
6	7	0	-3.231298	-0.243050	-0.159517
7	7	0	-5.792860	0.626644	0.793489
8	6	0	1.035225	2.533674	0.186644
9	6	0	1.681969	3.516126	-0.735732
10	6	0	0.264714	3.096564	-0.968710
11	6	0	0.475375	0.137703	-0.057611
12	6	0	2.738602	0.811945	0.293345
13	6	0	0.884918	-1.174437	-0.273870
14	6	0	-0.885906	0.452846	0.011154
15	6	0	-1.860151	-0.518336	-0.182657
16	6	0	-3.979941	-1.002631	0.863893
17	6	0	-3.587121	1.176946	-0.093671
18	6	0	3.221513	-0.495681	0.121898
19	6	0	2.302423	-1.546764	-0.254547
20	6	0	-5.469623	-0.797374	0.691892
21	6	0	-5.082731	1.347919	-0.264085
22	6	0	-0.088189	-2.158321	-0.490010
23	6	0	-1.407469	-1.825083	-0.459534
24	6	0	4.626233	-0.834143	0.344754
25	1	0	0.772266	2.864975	1.182591
26	1	0	2.427499	3.128324	-1.415853
27	1	0	1.881823	4.498419	-0.331538
28	1	0	-0.538422	3.779640	-0.729038
29	1	0	0.078861	2.429960	-1.800213
30	1	0	-1.183530	1.461899	0.241380
31	1	0	-3.671984	-0.659407	1.860969
32	1	0	-3.748130	-2.060657	0.780044
33	1	0	-3.068929	1.708580	-0.892728
34	1	0	-3.279732	1.611813	0.867859
35	1	0	-5.776164	-1.215557	-0.276702
36	1	0	-5.990912	-1.344719	1.477289
37	1	0	-5.322510	2.409364	-0.203006
38	1	0	-5.370316	0.990956	-1.262393
39	1	0	0.207140	-3.178791	-0.696875
40	1	0	-6.789729	0.736932	0.636293
41	1	0	6.340130	-0.066020	0.689910
42	8	0	3.490094	1.836337	0.649726
43	1	0	4.424720	1.552566	0.712886

A19		q(A)	q(A+1)	q(A-1)	fk+	fk-	fk0
1	F	-0.329847	-0.295815	-0.34767	0.02	0.03	0.03
2	O	-0.703839	-0.650003	-0.87274	0.17	0.05	0.11
3	O	-0.592945	-0.581178	-0.650755	0.06	0.01	0.03
4	O	-0.530123	-0.526271	-0.552526	0.02	0.00	0.01
5	N	0.166516	0.202601	0.099625	0.07	0.04	0.05
6	N	-0.088946	0.196697	-0.167589	0.08	0.29	0.18
7	N	-0.387234	-0.379964	-0.386878	0.00	0.01	0.00
8	C	-0.293287	-0.313511	-0.256984	0.04	0.02	0.03
9	C	-0.346109	-0.338017	-0.357291	0.01	0.01	0.01
10	C	-0.215084	-0.219047	-0.2187	0.00	0.00	0.00
11	C	0.078482	0.097818	0.049012	0.03	0.02	0.02
12	C	0.250992	0.256583	0.258696	0.01	0.01	0.00
13	C	0.309411	0.434251	0.196963	0.11	0.12	0.12
14	C	-0.453777	-0.417098	-0.472615	0.02	0.04	0.03
15	C	0.346477	0.311125	0.27182	0.07	0.04	0.02
16	C	-0.280292	-0.336748	-0.205249	0.08	0.06	0.07
17	C	-0.083343	-0.121844	-0.067941	0.02	0.04	0.03
18	C	-0.360909	-0.337695	-0.427778	0.07	0.02	0.05
19	C	0.27944	0.272711	0.232851	0.05	0.01	0.02
20	C	-0.298235	-0.288529	-0.32342	0.03	0.01	0.02
21	C	-0.501661	-0.469144	-0.512059	0.01	0.03	0.02
22	C	-0.34257	-0.324086	-0.471953	0.13	0.02	0.07
23	C	-0.234527	-0.185425	-0.192123	0.04	0.05	0.00
24	C	1.078358	1.083747	1.0395	0.04	0.01	0.02
25	H	0.210318	0.216625	0.198944	0.01	0.01	0.01
26	H	0.186809	0.190629	0.18153	0.01	0.00	0.00
27	H	0.177338	0.180884	0.172091	0.01	0.00	0.00
28	H	0.187158	0.191113	0.181754	0.01	0.00	0.00
29	H	0.194868	0.199332	0.188379	0.01	0.00	0.01
30	H	0.212735	0.247286	0.186063	0.03	0.03	0.03
31	H	0.178241	0.248224	0.163665	0.01	0.07	0.04
32	H	0.189479	0.224434	0.180728	0.01	0.03	0.02
33	H	0.193241	0.231956	0.183249	0.01	0.04	0.02
34	H	0.192504	0.261517	0.179425	0.01	0.07	0.04
35	H	0.165088	0.184693	0.160109	0.00	0.02	0.01
36	H	0.182509	0.198492	0.177811	0.00	0.02	0.01
37	H	0.185341	0.2018	0.180594	0.00	0.02	0.01
38	H	0.167315	0.187346	0.162395	0.00	0.02	0.01
39	H	0.199565	0.219197	0.171294	0.03	0.02	0.02
40	H	0.284819	0.295454	0.281788	0.00	0.01	0.01
41	H	0.413192	0.415808	0.403152	0.01	0.00	0.01
42	O	-0.460947	-0.443644	-0.485134	0.02	0.02	0.02
43	H	0.473478	0.477695	0.467972	0.01	0.00	0.00

XI

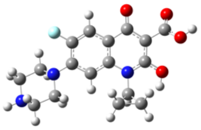

Standard orientation:

Center Number	Atomic Number	Atomic Type	Coordinates (Angstroms) X	Y	Z
1	9	0	-2.308546	-2.783587	-0.737434
2	8	0	2.662012	-2.717433	-0.436218
3	8	0	5.052385	-1.841452	0.796452
4	8	0	5.488435	0.103503	-0.196114
5	7	0	1.439409	1.137270	0.176120
6	7	0	-3.227286	-0.247492	-0.182484
7	7	0	-5.809942	0.620302	0.710862
8	6	0	1.025302	2.523565	0.289397
9	6	0	1.687516	3.557650	-0.589664
10	6	0	0.280721	3.134743	-0.855008
11	6	0	0.477916	0.134181	-0.021946
12	6	0	2.743489	0.801672	0.320113
13	6	0	0.889997	-1.180802	-0.229738
14	6	0	-0.885242	0.449032	0.025753
15	6	0	-1.856103	-0.522176	-0.181210
16	6	0	-3.993096	-1.001170	0.832233
17	6	0	-3.587567	1.171879	-0.133941
18	6	0	3.215494	-0.489843	0.132476
19	6	0	2.305775	-1.552428	-0.195737
20	6	0	-5.479724	-0.803424	0.628178
21	6	0	-5.079997	1.335966	-0.337024
22	6	0	-0.081401	-2.164620	-0.456231
23	6	0	-1.400219	-1.830544	-0.446936
24	6	0	4.644631	-0.815256	0.295409
25	1	0	0.739664	2.816903	1.291568
26	1	0	2.441774	3.208328	-1.282729
27	1	0	1.869635	4.524261	-0.140853
28	1	0	-0.527231	3.805565	-0.599160
29	1	0	0.113544	2.500793	-1.715291
30	1	0	-1.188948	1.457343	0.251473
31	1	0	-3.706874	-0.647515	1.832166
32	1	0	-3.755084	-2.058918	0.763068
33	1	0	-3.054779	1.699322	-0.926073
34	1	0	-3.301555	1.614683	0.830607
35	1	0	-5.764487	-1.231226	-0.342886
36	1	0	-6.014862	-1.346002	1.407538
37	1	0	-5.325289	2.396844	-0.289846
38	1	0	-5.344157	0.969502	-1.338348
39	1	0	0.216835	-3.185567	-0.656717
40	1	0	-6.803569	0.725784	0.531557
41	1	0	6.395347	-0.200329	-0.025507
42	8	0	3.594133	1.747436	0.693750
43	1	0	3.151013	2.604959	0.795136

XI		q(A)	q(A+1)	q(A-1)	fk+	fk-	fk0
1	F	-0.330089	-0.296798	-0.349303	0.02	0.03	0.03
2	O	-0.726567	-0.674827	-0.894482	0.17	0.05	0.11
3	O	-0.563135	-0.55216	-0.602097	0.04	0.01	0.02
4	O	-0.427934	-0.423995	-0.444949	0.02	0.00	0.01
5	N	0.261805	0.29832	0.192358	0.07	0.04	0.05
6	N	-0.084697	0.205284	-0.163852	0.08	0.29	0.18
7	N	-0.386389	-0.379274	-0.386148	0.00	0.01	0.00
8	C	-0.482586	-0.503441	-0.433782	0.05	0.02	0.03
9	C	-0.454883	-0.44785	-0.461695	0.01	0.01	0.01
10	C	-0.196829	-0.202281	-0.198216	0.00	0.01	0.00
11	C	0.099159	0.115758	0.067437	0.03	0.02	0.02
12	C	-0.298543	-0.320821	-0.205181	0.09	0.02	0.06
13	C	0.38143	0.506204	0.266207	0.12	0.12	0.12
14	C	-0.422527	-0.401354	-0.458766	0.04	0.02	0.03
15	C	0.252137	0.218704	0.191102	0.06	0.03	0.01
16	C	-0.276156	-0.336352	-0.201131	0.08	0.06	0.07
17	C	-0.081885	-0.122488	-0.066159	0.02	0.04	0.03
18	C	0.124589	0.173595	-0.025234	0.15	0.05	0.10
19	C	0.53406	0.533515	0.468862	0.07	0.00	0.03
20	C	-0.29872	-0.289039	-0.323106	0.02	0.01	0.02
21	C	-0.505543	-0.467225	-0.521361	0.02	0.04	0.03
22	C	-0.276458	-0.244265	-0.42993	0.15	0.03	0.09
23	C	-0.297951	-0.251857	-0.23476	0.06	0.05	0.01
24	C	0.88915	0.89293	0.866648	0.02	0.00	0.01
25	H	0.231977	0.23851	0.216605	0.02	0.01	0.01
26	H	0.215726	0.219726	0.208889	0.01	0.00	0.01
27	H	0.198624	0.202371	0.192009	0.01	0.00	0.01
28	H	0.194863	0.198927	0.188394	0.01	0.00	0.01
29	H	0.203184	0.207795	0.195735	0.01	0.00	0.01
30	H	0.213868	0.249251	0.184098	0.03	0.04	0.03
31	H	0.178058	0.248967	0.162898	0.02	0.07	0.04
32	H	0.189416	0.224927	0.180188	0.01	0.04	0.02
33	H	0.193298	0.232385	0.183108	0.01	0.04	0.02
34	H	0.192915	0.262727	0.179435	0.01	0.07	0.04
35	H	0.165013	0.184973	0.159872	0.01	0.02	0.01
36	H	0.182414	0.198614	0.177576	0.00	0.02	0.01
37	H	0.185378	0.20206	0.180473	0.00	0.02	0.01
38	H	0.1674	0.187731	0.162338	0.01	0.02	0.01
39	H	0.200279	0.21975	0.170658	0.03	0.02	0.02
40	H	0.284659	0.295442	0.281523	0.00	0.01	0.01
41	H	0.378405	0.380807	0.36987	0.01	0.00	0.01
42	O	-0.443027	-0.426338	-0.471663	0.03	0.02	0.02
43	H	0.436113	0.441092	0.425529	0.01	0.00	0.01

XIII

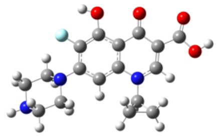

Standard orientation:

Center Number	Atomic Number	Atomic Type	Coordinates (Angstroms) X	Y	Z
1	9	0	-2.112382	-2.680972	-0.495219
2	8	0	2.943542	-2.451399	-0.252371
3	8	0	5.440735	-1.357932	0.266565
4	8	0	5.470439	0.862250	0.450489
5	7	0	1.529905	1.358296	0.174714
6	7	0	-3.088056	-0.116169	-0.152804
7	7	0	-5.715646	0.744075	0.598965
8	6	0	1.064617	2.726106	0.322980
9	6	0	1.827574	3.825104	-0.338480
10	6	0	0.480060	3.420188	-0.865041
11	6	0	0.602329	0.322135	0.018323
12	6	0	2.832538	1.093648	0.269071
13	6	0	1.072304	-0.999565	-0.113703
14	6	0	-0.757709	0.631998	0.026974
15	6	0	-1.715512	-0.365177	-0.114289
16	6	0	-3.865493	-0.815419	0.891622
17	6	0	-3.479300	1.294725	-0.214746
18	6	0	3.372060	-0.162756	0.158900
19	6	0	2.510409	-1.306161	-0.084801
20	6	0	-5.348813	-0.672679	0.627991
21	6	0	-4.968488	1.405620	-0.472316
22	6	0	0.092603	-2.007997	-0.269549
23	6	0	-1.241912	-1.671456	-0.272883
24	6	0	4.825687	-0.311714	0.288847
25	1	0	0.619890	2.918997	1.290753
26	1	0	2.688229	3.553391	-0.934343
27	1	0	1.915367	4.750880	0.211442
28	1	0	-0.370361	4.063381	-0.686723
29	1	0	0.464610	2.862376	-1.791912
30	1	0	3.462908	1.953675	0.444422
31	1	0	-1.070719	1.652821	0.163278
32	1	0	-3.621191	-0.380324	1.870213
33	1	0	-3.599386	-1.868511	0.909468
34	1	0	-2.937142	1.778173	-1.027839
35	1	0	-3.230798	1.810100	0.723516
36	1	0	-5.590069	-1.175822	-0.318430
37	1	0	-5.895689	-1.173456	1.426899
38	1	0	-5.240263	2.460571	-0.505410
39	1	0	-5.190397	0.965340	-1.454001
40	1	0	-6.704795	0.810453	0.380437
41	1	0	6.416372	0.667306	0.543889
42	8	0	0.440093	-3.305902	-0.436812
43	1	0	-0.364728	-3.839731	-0.511187

XIII		q(A)	q(A+1)	q(A-1)	fk+	fk-	fk0
1	F	-0.355827	-0.302688	-0.370666	0.01	0.05	0.03
2	O	-0.690021	-0.581507	-0.878101	0.19	0.11	0.15
3	O	-0.609358	-0.566385	-0.634883	0.03	0.04	0.03
4	O	-0.491159	-0.475456	-0.500592	0.01	0.02	0.01
5	N	0.382869	0.481532	0.311764	0.07	0.10	0.08
6	N	-0.081918	-0.031969	-0.156214	0.07	0.05	0.06
7	N	-0.383779	-0.382214	-0.383144	0.00	0.00	0.00
8	C	-0.343984	-0.385997	-0.292328	0.05	0.04	0.05
9	C	-0.30982	-0.300388	-0.309816	0.00	0.01	0.00
10	C	-0.254239	-0.241499	-0.268991	0.01	0.01	0.01
11	C	0.057938	0.10237	0.02632	0.03	0.04	0.04
12	C	-0.305345	-0.314933	-0.391275	0.09	0.01	0.04
13	C	0.298479	0.370934	0.220135	0.08	0.07	0.08
14	C	-0.468712	-0.389764	-0.516801	0.05	0.08	0.06
15	C	0.330518	0.305361	0.301378	0.03	0.03	0.00
16	C	-0.281242	-0.314783	-0.213549	0.07	0.03	0.05
17	C	-0.077118	-0.09566	-0.063761	0.01	0.02	0.02
18	C	-0.031639	0.10437	-0.060906	0.03	0.14	0.08
19	C	0.238507	0.196771	0.18293	0.06	0.04	0.01
20	C	-0.290697	-0.287139	-0.319891	0.03	0.00	0.02
21	C	-0.520936	-0.506482	-0.530287	0.01	0.01	0.01
22	C	0.072946	0.133063	-0.025473	0.10	0.06	0.08
23	C	-0.124126	-0.038926	-0.129052	0.00	0.09	0.05
24	C	0.877236	0.893333	0.867949	0.01	0.02	0.01
25	H	0.207447	0.231407	0.185229	0.02	0.02	0.02
26	H	0.176955	0.186166	0.168823	0.01	0.01	0.01
27	H	0.17141	0.17914	0.165319	0.01	0.01	0.01
28	H	0.181627	0.19113	0.174289	0.01	0.01	0.01
29	H	0.189634	0.199802	0.182231	0.01	0.01	0.01
30	H	0.214459	0.23435	0.184217	0.03	0.02	0.03
31	H	0.211578	0.247556	0.185509	0.03	0.04	0.03
32	H	0.178283	0.187946	0.165602	0.01	0.01	0.01
33	H	0.189931	0.195001	0.182379	0.01	0.01	0.01
34	H	0.192105	0.2008	0.183099	0.01	0.01	0.01
35	H	0.194548	0.205567	0.18236	0.01	0.01	0.01
36	H	0.164551	0.16818	0.160099	0.00	0.00	0.00
37	H	0.182531	0.186041	0.17823	0.00	0.00	0.00
38	H	0.185941	0.189567	0.181572	0.00	0.00	0.00
39	H	0.167825	0.171702	0.163285	0.00	0.00	0.00
40	H	0.284177	0.28668	0.281392	0.00	0.00	0.00
41	H	0.389277	0.39596	0.382735	0.01	0.01	0.01
42	O	-0.487	-0.409107	-0.52514	0.04	0.08	0.06
43	H	0.366148	0.380171	0.354022	0.01	0.01	0.01

A17

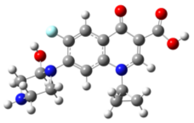

Standard orientation:

Center Number	Atomic Number	Atomic Type	Coordinates (Angstroms) X	Y	Z
1	9	0	-1.971836	-2.678574	-0.864809
2	8	0	2.972563	-2.611855	-0.409748
3	8	0	5.538836	-1.620513	0.112467
4	8	0	5.649973	0.579153	0.461429
5	7	0	1.719105	1.226798	0.251645
6	7	0	-2.938343	-0.162897	-0.298190
7	7	0	-5.461458	1.001976	0.401771
8	6	0	1.287896	2.594074	0.484267
9	6	0	2.069675	3.711854	-0.121830
10	6	0	0.711476	3.363948	-0.660923
11	6	0	0.768218	0.231822	0.008903
12	6	0	3.011805	0.912262	0.318126
13	6	0	1.197125	-1.077950	-0.220229
14	6	0	-0.598047	0.541569	0.010755
15	6	0	-1.548089	-0.430064	-0.247005
16	6	0	-3.652492	-0.560974	0.936972
17	6	0	-3.272746	1.236859	-0.611509
18	6	0	3.516007	-0.354193	0.118972
19	6	0	2.620680	-1.446588	-0.188141
20	6	0	-5.145495	-0.383969	0.733345
21	6	0	-4.763479	1.373750	-0.829789
22	6	0	0.239244	-2.060844	-0.497099
23	6	0	-1.082928	-1.729552	-0.521441
24	6	0	4.965435	-0.556655	0.222380
25	1	0	0.852891	2.739641	1.464401
26	1	0	2.921864	3.453744	-0.735673
27	1	0	2.178852	4.604638	0.476591
28	1	0	-0.124409	4.013606	-0.442209
29	1	0	0.678459	2.857002	-1.616244
30	1	0	3.672510	1.736483	0.546274
31	1	0	-0.914472	1.551097	0.221085
32	1	0	-3.305216	0.065331	1.768759
33	1	0	-2.729343	1.524430	-1.511789
34	1	0	-2.976263	1.910715	0.202840
35	1	0	-5.455998	-1.087936	-0.053226
36	1	0	-5.645127	-0.663230	1.660668
37	1	0	-4.991054	2.411366	-1.073442
38	1	0	-5.065024	0.746974	-1.679158
39	1	0	0.548367	-3.075326	-0.712481
40	1	0	-6.461574	1.068738	0.242441
41	1	0	6.591238	0.351268	0.523969
42	8	0	-3.352226	-1.881556	1.308148
43	1	0	-3.683185	-2.476434	0.621020

A17		q(A)	q(A+1)	q(A-1)	fk+	fk-	fk0
1	F	-0.336553	-0.296346	-0.357014	0.02	0.04	0.03
2	O	-0.692118	-0.507591	-0.863653	0.17	0.18	0.18
3	O	-0.605576	-0.553769	-0.632073	0.03	0.05	0.04
4	O	-0.48779	-0.46882	-0.497607	0.01	0.02	0.01
5	N	0.394012	0.50819	0.322254	0.07	0.11	0.09
6	N	-0.22652	-0.17869	-0.303811	0.08	0.05	0.06
7	N	-0.401524	-0.3997	-0.403472	0.00	0.00	0.00
8	C	-0.351383	-0.385449	-0.295166	0.06	0.03	0.05
9	C	-0.335485	-0.326729	-0.334768	0.00	0.01	0.00
10	C	-0.250644	-0.232925	-0.2594	0.01	0.02	0.01
11	C	-0.074156	-0.043586	-0.054571	0.02	0.03	0.01
12	C	-0.604384	-0.66832	-0.644457	0.04	0.06	0.01
13	C	0.498105	0.634073	0.368458	0.13	0.14	0.13
14	C	-0.267918	-0.235227	-0.379569	0.11	0.03	0.07
15	C	-0.095216	-0.118418	-0.132543	0.04	0.02	0.01
16	C	0.019575	0.005456	0.063801	0.04	0.01	0.03
17	C	-0.017318	-0.028331	-0.003742	0.01	0.01	0.01
18	C	0.332835	0.554908	0.25012	0.08	0.22	0.15
19	C	0.101137	0.03056	0.080954	0.02	0.07	0.03
20	C	-0.224213	-0.226254	-0.232993	0.01	0.00	0.00
21	C	-0.57232	-0.558014	-0.580644	0.01	0.01	0.01
22	C	-0.104452	-0.04433	-0.248914	0.14	0.06	0.10
23	C	-0.027135	0.001672	0.020896	0.05	0.03	0.01
24	C	0.89259	0.909528	0.883674	0.01	0.02	0.01
25	H	0.208603	0.236805	0.188604	0.02	0.03	0.02
26	H	0.177829	0.188545	0.170564	0.01	0.01	0.01
27	H	0.173038	0.18209	0.167103	0.01	0.01	0.01
28	H	0.183007	0.194347	0.176106	0.01	0.01	0.01
29	H	0.191823	0.203891	0.184936	0.01	0.01	0.01
30	H	0.215454	0.23868	0.189774	0.03	0.02	0.02
31	H	0.213306	0.237392	0.180563	0.03	0.02	0.03
32	H	0.184233	0.193274	0.171751	0.01	0.01	0.01
33	H	0.200392	0.206961	0.191069	0.01	0.01	0.01
34	H	0.195222	0.20314	0.185058	0.01	0.01	0.01
35	H	0.177686	0.181037	0.172878	0.00	0.00	0.00
36	H	0.190309	0.193477	0.185537	0.00	0.00	0.00
37	H	0.186682	0.189882	0.181887	0.00	0.00	0.00
38	H	0.167586	0.170952	0.162796	0.00	0.00	0.00
39	H	0.199742	0.220917	0.170798	0.03	0.02	0.03
40	H	0.289907	0.292003	0.286974	0.00	0.00	0.00
41	H	0.389958	0.397999	0.383773	0.01	0.01	0.01
42	O	-0.426741	-0.423738	-0.431148	0.00	0.00	0.00
43	H	0.318411	0.320461	0.315219	0.00	0.00	0.00

B13

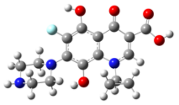

Standard orientation:

Center Number	Atomic Number	Atomic Type	Coordinates (Angstroms) X	Y	Z
1	9	0	-2.133668	-2.561630	-0.537447
2	8	0	2.931689	-2.438238	-0.561271
3	8	0	5.416656	-1.571837	0.325976
4	8	0	5.561594	0.635856	0.603565
5	7	0	1.643597	1.332643	0.283509
6	7	0	-3.034493	-0.083321	0.074849
7	7	0	-5.798562	0.457933	0.303810
8	6	0	1.320066	2.748909	0.128338
9	6	0	2.093312	3.530590	-0.885643
10	6	0	0.653147	3.175668	-1.133902
11	6	0	0.674336	0.328476	0.141143
12	6	0	2.936163	1.007611	0.360274
13	6	0	1.094675	-0.987019	-0.158716
14	6	0	-0.682582	0.625686	0.346598
15	6	0	-1.662624	-0.326399	0.059283
16	6	0	-3.841111	-0.766373	1.091891
17	6	0	-3.562303	1.235319	-0.272232
18	6	0	3.423184	-0.263375	0.210123
19	6	0	2.529160	-1.328602	-0.200269
20	6	0	-5.274274	-0.875479	0.612035
21	6	0	-4.992115	1.072422	-0.754824
22	6	0	0.103694	-1.972233	-0.350129
23	6	0	-1.218555	-1.607598	-0.270646
24	6	0	4.862348	-0.493334	0.371843
25	1	0	1.036387	3.241489	1.045288
26	1	0	2.838881	3.005490	-1.467522
27	1	0	2.339909	4.549868	-0.624190
28	1	0	-0.101177	3.945049	-1.050640
29	1	0	0.459404	2.404436	-1.868397
30	1	0	3.604774	1.841025	0.524589
31	1	0	-3.806028	-0.202955	2.034639
32	1	0	-3.431893	-1.758796	1.272867
33	1	0	-2.944179	1.668758	-1.058766
34	1	0	-3.571034	1.921249	0.583959
35	1	0	-5.297656	-1.534448	-0.266903
36	1	0	-5.882071	-1.329111	1.395040
37	1	0	-5.400374	2.055359	-0.990668
38	1	0	-4.994848	0.469056	-1.672427
39	1	0	-6.746374	0.351510	-0.043237
40	1	0	6.493384	0.389682	0.716736
41	8	0	-1.032875	1.831815	0.899038
42	1	0	-1.770931	1.685496	1.506084
43	8	0	0.418416	-3.263361	-0.633240
44	1	0	-0.399915	-3.778767	-0.678149

B13		q(A)	q(A+1)	q(A-1)	fk+	fk-	fk0
1	F	-0.349844	-0.327343	-0.363466	0.01	0.02	0.02
2	O	-0.672189	-0.634485	-0.860104	0.19	0.04	0.11
3	O	-0.598696	-0.571898	-0.630815	0.03	0.03	0.03
4	O	-0.487832	-0.478731	-0.500344	0.01	0.01	0.01
5	N	0.374657	0.450422	0.287812	0.09	0.08	0.08
6	N	-0.004772	0.113827	-0.089754	0.08	0.12	0.10
7	N	-0.380391	-0.375839	-0.380847	0.00	0.00	0.00
8	C	-0.401614	-0.454442	-0.352858	0.05	0.05	0.05
9	C	-0.260351	-0.254387	-0.268299	0.01	0.01	0.01
10	C	-0.291775	-0.293646	-0.297421	0.01	0.00	0.00
11	C	-0.501016	-0.49644	-0.444411	0.06	0.00	0.03
12	C	-0.732129	-0.756008	-0.677109	0.06	0.02	0.04
13	C	0.960416	1.135319	0.693551	0.27	0.17	0.22
14	C	0.10014	0.189604	0.065283	0.03	0.09	0.06
15	C	-0.020682	-0.03574	-0.041149	0.02	0.02	0.00
16	C	-0.317385	-0.339766	-0.250669	0.07	0.02	0.04
17	C	-0.062277	-0.114459	-0.05518	0.01	0.05	0.03
18	C	0.351796	0.443603	0.236732	0.12	0.09	0.10
19	C	0.351548	0.357554	0.263735	0.09	0.01	0.05
20	C	-0.242519	-0.247898	-0.276936	0.03	0.01	0.01
21	C	-0.518856	-0.500924	-0.508672	0.01	0.02	0.00
22	C	0.036965	0.082206	-0.008466	0.05	0.05	0.05
23	C	-0.190818	-0.207692	-0.153879	0.04	0.02	0.03
24	C	0.849666	0.854805	0.836408	0.01	0.01	0.01
25	H	0.197885	0.21312	0.182243	0.02	0.02	0.02
26	H	0.179131	0.186372	0.171682	0.01	0.01	0.01
27	H	0.166867	0.173501	0.160241	0.01	0.01	0.01
28	H	0.178797	0.186147	0.171717	0.01	0.01	0.01
29	H	0.198143	0.2074	0.190217	0.01	0.01	0.01
30	H	0.221095	0.235176	0.193918	0.03	0.01	0.02
31	H	0.178983	0.207802	0.165075	0.01	0.03	0.02
32	H	0.189333	0.205012	0.18169	0.01	0.02	0.01
33	H	0.200302	0.220173	0.191159	0.01	0.02	0.01
34	H	0.179333	0.206866	0.166039	0.01	0.03	0.02
35	H	0.161152	0.170238	0.156745	0.00	0.01	0.01
36	H	0.180724	0.188532	0.176411	0.00	0.01	0.01
37	H	0.183659	0.192441	0.178376	0.01	0.01	0.01
38	H	0.166345	0.176232	0.16113	0.01	0.01	0.01
39	H	0.283258	0.28837	0.280508	0.00	0.01	0.00
40	H	0.390193	0.394409	0.383107	0.01	0.00	0.01
41	O	-0.499017	-0.366086	-0.532234	0.03	0.13	0.08
42	H	0.381926	0.405706	0.376789	0.01	0.02	0.01
43	O	-0.495938	-0.410545	-0.53267	0.04	0.09	0.06
44	H	0.365788	0.381492	0.354714	0.01	0.02	0.01

A1’

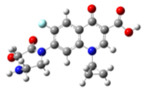

Standard orientation:

Center Number	Atomic Number	Atomic Type	Coordinates (Angstroms) X	Y	Z
1	9	0	-1.847923	-2.532202	-0.885750
2	8	0	3.108756	-2.645129	-0.476946
3	8	0	5.706441	-1.758976	0.056198
4	8	0	5.889098	0.408818	0.546424
5	7	0	1.987444	1.212029	0.303493
6	7	0	-2.686107	0.020087	-0.280859
7	7	0	-5.263446	1.049628	0.025788
8	6	0	1.601271	2.582485	0.590821
9	6	0	2.426845	3.697381	0.040837
10	6	0	1.062083	3.422113	-0.523041
11	6	0	1.004349	0.260452	0.024530
12	6	0	3.267767	0.848309	0.367530
13	6	0	1.385305	-1.059235	-0.240247
14	6	0	-0.349341	0.617337	0.030645
15	6	0	-1.313207	-0.327510	-0.247542
16	6	0	-3.551054	-0.657570	0.512205
17	6	0	-3.059603	1.284281	-0.945120
18	6	0	3.728714	-0.426969	0.126994
19	6	0	2.798834	-1.477291	-0.218895
20	6	0	-5.039118	-0.329067	0.396948
21	6	0	-4.542188	1.312366	-1.220990
22	6	0	0.400922	-2.007451	-0.535903
23	6	0	-0.911845	-1.635187	-0.549122
24	6	0	5.169900	-0.684724	0.228967
25	1	0	1.163544	2.699824	1.573517
26	1	0	3.274622	3.437937	-0.578423
27	1	0	2.562616	4.556901	0.681006
28	1	0	0.247461	4.090077	-0.280981
29	1	0	1.020574	2.961331	-1.501170
30	1	0	3.955788	1.640099	0.627539
31	1	0	-0.650714	1.627605	0.265598
32	1	0	-2.497430	1.345890	-1.875880
33	1	0	-2.780340	2.124231	-0.306331
34	1	0	-5.431829	-1.025928	-0.360926
35	1	0	-4.809375	2.300949	-1.588181
36	1	0	-4.787387	0.578728	-1.999049
37	1	0	0.678947	-3.027186	-0.767997
38	1	0	-6.259916	1.149723	-0.152122
39	1	0	6.821693	0.145570	0.600823
40	8	0	-3.205016	-1.550750	1.275688
41	8	0	-5.667899	-0.570739	1.631260
42	1	0	-5.271024	-1.372271	2.002672

A1'		q(A)	q(A+1)	q(A-1)	fk+	fk-	fk0
1	F	-0.307283	-0.272981	-0.328953	0.02	0.03	0.03
2	O	-0.681564	-0.492403	-0.837985	0.16	0.19	0.17
3	O	-0.604283	-0.543762	-0.630203	0.03	0.06	0.04
4	O	-0.486884	-0.464908	-0.49674	0.01	0.02	0.02
5	N	0.407354	0.53378	0.334958	0.07	0.13	0.10
6	N	0.189043	0.223515	0.104834	0.08	0.03	0.06
7	N	-0.418602	-0.417397	-0.421703	0.00	0.00	0.00
8	C	-0.361268	-0.397829	-0.303398	0.06	0.04	0.05
9	C	-0.340194	-0.329716	-0.336633	0.00	0.01	0.00
10	C	-0.242476	-0.223556	-0.253235	0.01	0.02	0.01
11	C	-0.15403	-0.141172	-0.115201	0.04	0.01	0.01
12	C	-0.544957	-0.605222	-0.579111	0.03	0.06	0.01
13	C	0.438142	0.555849	0.314255	0.12	0.12	0.12
14	C	-0.226735	-0.176074	-0.369862	0.14	0.05	0.10
15	C	-0.428897	-0.464405	-0.426972	0.00	0.04	0.02
16	C	0.330428	0.322697	0.325764	0.00	0.01	0.00
17	C	-0.032791	-0.03733	-0.015523	0.02	0.00	0.01
18	C	0.342514	0.596683	0.256636	0.09	0.25	0.17
19	C	0.082946	0.009435	0.07406	0.01	0.07	0.03
20	C	0.090248	0.088483	0.100478	0.01	0.00	0.01
21	C	-0.542174	-0.539433	-0.557959	0.02	0.00	0.01
22	C	-0.049089	0.001758	-0.20082	0.15	0.05	0.10
23	C	0.113137	0.139582	0.162364	0.05	0.03	0.01
24	C	0.905783	0.925587	0.893968	0.01	0.02	0.02
25	H	0.209989	0.241684	0.191656	0.02	0.03	0.03
26	H	0.178388	0.189992	0.17179	0.01	0.01	0.01
27	H	0.173878	0.18373	0.168168	0.01	0.01	0.01
28	H	0.18401	0.196612	0.177595	0.01	0.01	0.01
29	H	0.190441	0.203849	0.184086	0.01	0.01	0.01
30	H	0.216535	0.24184	0.193778	0.02	0.03	0.02
31	H	0.222971	0.246496	0.188614	0.03	0.02	0.03
32	H	0.209131	0.21272	0.201307	0.01	0.00	0.01
33	H	0.205022	0.208756	0.197472	0.01	0.00	0.01
34	H	0.196451	0.199358	0.188663	0.01	0.00	0.01
35	H	0.201921	0.204041	0.197213	0.00	0.00	0.00
36	H	0.188901	0.191164	0.183928	0.00	0.00	0.00
37	H	0.203372	0.221785	0.173541	0.03	0.02	0.02
38	H	0.304905	0.306567	0.301109	0.00	0.00	0.00
39	H	0.390876	0.400027	0.385061	0.01	0.01	0.01
40	O	-0.582073	-0.569644	-0.617688	0.04	0.01	0.02
41	O	-0.515203	-0.512836	-0.520219	0.01	0.00	0.00
42	H	0.34212	0.342678	0.340909	0.00	0.00	0.00

B8

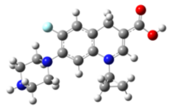

Standard orientation:

Center Number	Atomic Number	Atomic Type	Coordinates (Angstroms) X	Y	Z
1	9	0	-2.085602	-2.847874	-0.664805
2	8	0	5.370926	-1.908756	0.168805
3	8	0	5.619824	0.231740	0.773345
4	7	0	1.703479	1.088847	0.059723
5	7	0	-2.998520	-0.282506	-0.214968
6	7	0	-5.516821	0.681244	0.764409
7	6	0	1.284950	2.445103	0.308541
8	6	0	2.084908	3.554426	-0.293015
9	6	0	0.717449	3.236557	-0.828802
10	6	0	0.731696	0.075689	-0.121896
11	6	0	2.997312	0.738325	0.267213
12	6	0	1.141130	-1.244262	-0.316167
13	6	0	-0.625638	0.400002	-0.065518
14	6	0	-1.616097	-0.567399	-0.228913
15	6	0	-3.709366	-0.951204	0.892763
16	6	0	-3.336797	1.142266	-0.229155
17	6	0	3.473765	-0.516799	0.104012
18	6	0	2.598375	-1.631777	-0.396589
19	6	0	-5.203778	-0.749118	0.760664
20	6	0	-4.835606	1.319207	-0.364219
21	6	0	0.156917	-2.213606	-0.472657
22	6	0	-1.173735	-1.873414	-0.444011
23	6	0	4.868637	-0.806125	0.340640
24	1	0	0.852188	2.608959	1.289349
25	1	0	2.932914	3.277565	-0.904825
26	1	0	2.211839	4.446493	0.303269
27	1	0	-0.099279	3.908574	-0.604582
28	1	0	0.666273	2.736286	-1.786706
29	1	0	3.644503	1.549122	0.573849
30	1	0	-0.913678	1.423556	0.114964
31	1	0	-3.363047	-0.537815	1.850895
32	1	0	-3.485566	-2.015177	0.879940
33	1	0	-2.836373	1.618176	-1.073481
34	1	0	-3.000443	1.636573	0.693742
35	1	0	-5.546010	-1.237280	-0.162275
36	1	0	-5.701160	-1.231518	1.602251
37	1	0	-5.065728	2.384650	-0.369488
38	1	0	-5.159244	0.896462	-1.325151
39	1	0	0.429093	-3.248944	-0.644853
40	1	0	-6.517523	0.786714	0.629778
41	1	0	6.527588	-0.090479	0.883585
42	1	0	2.767510	-2.540842	0.185716
43	1	0	2.858861	-1.884403	-1.431427

B8		q(A)	q(A+1)	q(A-1)	fk+	fk-	fk0
1	F	-0.334026	-0.302462	-0.347509	0.01	0.03	0.02
2	O	-0.643634	-0.571094	-0.822492	0.18	0.07	0.13
3	O	-0.501961	-0.474671	-0.567765	0.07	0.03	0.05
4	N	0.289688	0.440954	0.185012	0.10	0.15	0.13
5	N	-0.144119	-0.088748	-0.170835	0.03	0.06	0.04
6	N	-0.386899	-0.385653	-0.386769	0.00	0.00	0.00
7	C	-0.293952	-0.338836	-0.219011	0.07	0.04	0.06
8	C	-0.315185	-0.305831	-0.300392	0.01	0.01	0.00
9	C	-0.275766	-0.253413	-0.296734	0.02	0.02	0.02
10	C	0.113072	0.141275	0.172575	0.06	0.03	0.02
11	C	-0.487519	-0.513892	-0.764495	0.28	0.03	0.13
12	C	0.685575	0.794788	0.583146	0.10	0.11	0.11
13	C	-0.405197	-0.385672	-0.464826	0.06	0.02	0.04
14	C	0.37524	0.377044	0.383382	0.01	0.00	0.00
15	C	-0.257215	-0.293747	-0.237611	0.02	0.04	0.03
16	C	-0.030662	-0.042734	-0.013142	0.02	0.01	0.01
17	C	0.506497	0.718516	0.406044	0.10	0.21	0.16
18	C	-1.06415	-1.160707	-0.975745	0.09	0.10	0.09
19	C	-0.300479	-0.293914	-0.303612	0.00	0.01	0.00
20	C	-0.56933	-0.554936	-0.577422	0.01	0.01	0.01
21	C	-0.304743	-0.258909	-0.323367	0.02	0.05	0.03
22	C	-0.241814	-0.1799	-0.271174	0.03	0.06	0.05
23	C	0.602858	0.617976	0.55171	0.05	0.02	0.03
24	H	0.192616	0.228854	0.170915	0.02	0.04	0.03
25	H	0.1714	0.183479	0.159878	0.01	0.01	0.01
26	H	0.167104	0.177261	0.161476	0.01	0.01	0.01
27	H	0.176098	0.188512	0.167339	0.01	0.01	0.01
28	H	0.182518	0.195914	0.174403	0.01	0.01	0.01
29	H	0.201088	0.229292	0.145705	0.06	0.03	0.04
30	H	0.204702	0.227517	0.188149	0.02	0.02	0.02
31	H	0.172986	0.183624	0.170143	0.00	0.01	0.01
32	H	0.185336	0.191576	0.183398	0.00	0.01	0.00
33	H	0.190657	0.198516	0.186907	0.00	0.01	0.01
34	H	0.187136	0.197404	0.183791	0.00	0.01	0.01
35	H	0.162921	0.166641	0.161461	0.00	0.00	0.00
36	H	0.180548	0.184032	0.179197	0.00	0.00	0.00
37	H	0.183624	0.187095	0.182273	0.00	0.00	0.00
38	H	0.164975	0.168759	0.163413	0.00	0.00	0.00
39	H	0.184587	0.202476	0.175101	0.01	0.02	0.01
40	H	0.282439	0.284798	0.281344	0.00	0.00	0.00
41	H	0.378396	0.388391	0.359082	0.02	0.01	0.01
42	H	0.204207	0.256274	0.180426	0.02	0.05	0.04
43	H	0.210383	0.274148	0.186631	0.02	0.06	0.04

B1

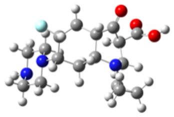

Standard orientation:

Center Number	Atomic Number	Atomic Type	X	Y	Z
1	9	0	-2.495811	-0.711626	-2.648021
2	8	0	2.649396	-2.047066	-1.480246
3	8	0	2.890617	-3.162062	1.619472
4	8	0	4.335963	-1.571649	1.010811
5	7	0	1.985706	1.261820	0.018116
6	7	0	-2.989440	0.328103	-0.217978
7	7	0	-4.625227	-0.523383	1.980268
8	6	0	2.012047	2.609787	0.526432
9	6	0	3.342930	3.272372	0.697590
10	6	0	2.409442	3.710872	-0.402189
11	6	0	0.768142	0.977119	-0.757296
12	6	0	2.168858	0.321752	1.124449
13	6	0	1.011192	-0.319584	-1.560021
14	6	0	-0.532043	0.906627	0.050318
15	6	0	-1.760204	0.783802	-0.881029
16	6	0	-2.955037	-1.052219	0.273155
17	6	0	-3.343878	1.229129	0.881234
18	6	0	2.034320	-1.140275	0.647125
19	6	0	1.988341	-1.233917	-0.870361
20	6	0	-4.297642	-1.425923	0.873924
21	6	0	-4.686677	0.849248	1.471235
22	6	0	-0.297960	-1.096836	-1.806315
23	6	0	-1.376629	-0.073778	-2.106081
24	6	0	3.107155	-2.066902	1.157043
25	1	0	1.260459	2.851215	1.275917
26	1	0	4.214409	2.701077	0.407016
27	1	0	3.475024	3.927418	1.547304
28	1	0	1.908390	4.663210	-0.298936
29	1	0	2.670157	3.428106	-1.413347
30	1	0	3.163577	0.486819	1.535803
31	1	0	-0.506079	0.054418	0.734232
32	1	0	-2.175496	-1.199132	1.035881
33	1	0	-2.748250	-1.725362	-0.557050
34	1	0	-3.383488	2.250605	0.495794
35	1	0	-2.591647	1.200036	1.683565
36	1	0	-5.060914	-1.388293	0.084111
37	1	0	-4.249105	-2.449386	1.246771
38	1	0	-4.924950	1.527245	2.291310
39	1	0	-5.461998	0.961299	0.700926
40	1	0	-0.176855	-1.770110	-2.654895
41	1	0	-5.544707	-0.773224	2.330470
42	1	0	4.981571	-2.231471	1.315011
43	1	0	1.445182	0.496830	1.929413
44	1	0	1.077334	-1.553631	0.980379
45	1	0	0.672212	1.795734	-1.474832
46	1	0	1.456330	-0.066023	-2.524003
47	1	0	-0.633821	1.805942	0.658677
48	1	0	-0.562954	-1.693641	-0.930232
49	1	0	-1.989383	1.785867	-1.253478
50	1	0	-1.011923	0.591879	-2.892044

B1		q(A)	q(A+1)	q(A-1)	fk+	fk-	fk0
1	F	-0.245465	-0.225099	-0.257531	0.01	0.02	0.02
2	O	-0.491839	-0.483194	-0.855881	0.36	0.01	0.19
3	O	-0.499441	-0.497374	-0.533467	0.03	0.00	0.02
4	O	-0.40609	-0.404804	-0.431567	0.03	0.00	0.01
5	N	-0.105211	-0.074146	-0.162381	0.06	0.03	0.04
6	N	-0.01484	0.443463	-0.023559	0.01	0.46	0.23
7	N	-0.400883	-0.391361	-0.401534	0.00	0.01	0.01
8	C	0.161654	0.145882	0.242501	0.08	0.02	0.05
9	C	-0.362242	-0.359213	-0.381466	0.02	0.00	0.01
10	C	-0.396032	-0.39148	-0.413873	0.02	0.00	0.01
11	C	-0.177293	-0.178399	-0.249487	0.07	0.00	0.04
12	C	-0.308068	-0.314973	-0.333206	0.03	0.01	0.01
13	C	-0.250898	-0.240214	-0.263995	0.01	0.01	0.01
14	C	0.132684	0.168934	0.133515	0.00	0.04	0.02
15	C	-0.533872	-0.698832	-0.557131	0.02	0.16	0.07
16	C	-0.096745	-0.183873	-0.078782	0.02	0.09	0.05
17	C	0.015959	-0.060183	0.016255	0.00	0.08	0.04
18	C	-0.208817	-0.215166	-0.089975	0.12	0.01	0.06
19	C	0.421732	0.411632	0.167264	0.25	0.01	0.12
20	C	-0.477453	-0.449823	-0.487252	0.01	0.03	0.02
21	C	-0.542873	-0.51021	-0.543145	0.00	0.03	0.02
22	C	-0.085622	-0.10864	-0.095324	0.01	0.02	0.01
23	C	-0.573549	-0.541773	-0.474996	0.10	0.03	0.03
24	C	0.242522	0.244259	0.268965	0.03	0.00	0.01
25	H	0.155815	0.160296	0.145703	0.01	0.00	0.01
26	H	0.159672	0.162088	0.156936	0.00	0.00	0.00
27	H	0.157805	0.159868	0.154326	0.00	0.00	0.00
28	H	0.162432	0.163953	0.158709	0.00	0.00	0.00
29	H	0.167897	0.169857	0.164463	0.00	0.00	0.00
30	H	0.201808	0.206889	0.18427	0.02	0.01	0.01
31	H	0.211597	0.254336	0.188962	0.02	0.04	0.03
32	H	0.174906	0.282768	0.17089	0.00	0.11	0.06
33	H	0.188322	0.242284	0.186004	0.00	0.05	0.03
34	H	0.187143	0.242908	0.18535	0.00	0.06	0.03
35	H	0.171421	0.272598	0.169478	0.00	0.10	0.05
36	H	0.159725	0.191455	0.158377	0.00	0.03	0.02
37	H	0.177928	0.20204	0.17715	0.00	0.02	0.01
38	H	0.178812	0.202699	0.177937	0.00	0.02	0.01
39	H	0.160861	0.192097	0.159823	0.00	0.03	0.02
40	H	0.204129	0.216274	0.179414	0.02	0.01	0.02
41	H	0.287439	0.303955	0.286566	0.00	0.02	0.01
42	H	0.383642	0.384267	0.37628	0.01	0.00	0.00
43	H	0.209321	0.217478	0.182634	0.03	0.01	0.02
44	H	0.24888	0.254075	0.148816	0.10	0.01	0.05
45	H	0.196192	0.213971	0.170873	0.03	0.02	0.02
46	H	0.219906	0.228965	0.155043	0.06	0.01	0.04
47	H	0.190062	0.226326	0.177646	0.01	0.04	0.02
48	H	0.22901	0.247397	0.197909	0.03	0.02	0.02
49	H	0.196208	0.267737	0.187952	0.01	0.07	0.04
50	H	0.221746	0.248007	0.204542	0.02	0.03	0.02

A20

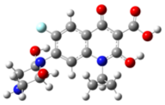

Standard orientation:

Center Number	Atomic Number	Atomic Type	Coordinates (Angstroms) X	Y	Z
1	9	0	-2.010135	-2.763708	-1.019594
2	8	0	2.944482	-2.592495	-0.798536
3	8	0	5.386943	-1.879902	0.173902
4	8	0	5.669980	0.281778	0.594946
5	7	0	1.653997	1.127663	0.262570
6	7	0	-2.999632	-0.343266	-0.149267
7	7	0	-5.371039	0.851704	0.862743
8	6	0	1.214781	2.502971	0.432290
9	6	0	1.815937	3.560055	-0.437535
10	6	0	0.403234	3.121078	-0.665710
11	6	0	0.713748	0.125586	-0.012997
12	6	0	2.959579	0.818903	0.394755
13	6	0	1.148999	-1.143535	-0.383483
14	6	0	-0.653970	0.389620	0.098939
15	6	0	-1.593232	-0.572009	-0.230370
16	6	0	-3.515148	-0.675467	1.202747
17	6	0	-3.394973	1.025953	-0.531351
18	6	0	3.474027	-0.454964	0.093699
19	6	0	2.582751	-1.479899	-0.400752
20	6	0	-5.018138	-0.517160	1.222823
21	6	0	-4.903586	1.135358	-0.490739
22	6	0	0.208127	-2.120434	-0.714809
23	6	0	-1.125306	-1.822341	-0.655096
24	6	0	4.887930	-0.781314	0.276598
25	1	0	0.969273	2.760347	1.454126
26	1	0	2.553726	3.235852	-1.158590
27	1	0	2.002016	4.519052	0.024945
28	1	0	-0.408896	3.767114	-0.361833
29	1	0	0.210810	2.509316	-1.537923
30	1	0	-0.985843	1.352725	0.456452
31	1	0	-3.053333	-0.005626	1.940959
32	1	0	-2.969203	1.770395	0.152196
33	1	0	-5.444732	-1.260557	0.535859
34	1	0	-5.368716	-0.733465	2.231248
35	1	0	-5.176872	2.152779	-0.767659
36	1	0	-5.317809	0.444385	-1.237216
37	1	0	0.533488	-3.105036	-1.023966
38	1	0	-6.381496	0.938444	0.878740
39	1	0	6.575851	-0.028390	0.755976
40	8	0	3.685647	1.826149	0.836455
41	1	0	4.626317	1.559336	0.884959
42	8	0	-3.222978	-2.011931	1.528515
43	1	0	-2.326682	-2.061718	1.882633
44	8	0	-2.976917	1.294621	-1.850365
45	1	0	-2.055795	1.585537	-1.840261

A20		q(A)	q(A+1)	q(A-1)	fk+	fk-	fk0
1	F	-0.298298	-0.249896	-0.318774	0.02	0.05	0.03
2	O	-0.688936	-0.535358	-0.848528	0.16	0.15	0.16
3	O	-0.598642	-0.554092	-0.646204	0.05	0.04	0.05
4	O	-0.551279	-0.535866	-0.5692	0.02	0.02	0.02
5	N	0.172389	0.285127	0.100121	0.07	0.11	0.09
6	N	-0.205355	-0.153301	-0.288362	0.08	0.05	0.07
7	N	-0.419708	-0.41815	-0.421515	0.00	0.00	0.00
8	C	-0.296825	-0.33495	-0.25801	0.04	0.04	0.04
9	C	-0.406177	-0.397041	-0.413282	0.01	0.01	0.01
10	C	-0.138801	-0.128935	-0.139486	0.00	0.01	0.01
11	C	-0.102545	-0.07581	-0.072011	0.03	0.03	0.00
12	C	0.207142	0.192125	0.207845	0.00	0.02	0.01
13	C	0.394704	0.517146	0.25999	0.13	0.12	0.13
14	C	-0.276412	-0.230894	-0.37093	0.09	0.05	0.07
15	C	-0.24914	-0.25821	-0.291256	0.04	0.01	0.02
16	C	-0.062571	-0.074372	-0.027391	0.04	0.01	0.02
17	C	0.174905	0.170607	0.185057	0.01	0.00	0.01
18	C	-0.292246	-0.154173	-0.358301	0.07	0.14	0.10
19	C	0.208449	0.15885	0.189	0.02	0.05	0.02
20	C	-0.216394	-0.218887	-0.223617	0.01	0.00	0.00
21	C	-0.465699	-0.463052	-0.459367	0.01	0.00	0.00
22	C	-0.11746	-0.066531	-0.2462	0.13	0.05	0.09
23	C	0.006387	0.043207	0.033524	0.03	0.04	0.00
24	C	1.142297	1.178191	1.100217	0.04	0.04	0.04
25	H	0.211986	0.241447	0.200117	0.01	0.03	0.02
26	H	0.188097	0.200945	0.182587	0.01	0.01	0.01
27	H	0.179051	0.189994	0.173376	0.01	0.01	0.01
28	H	0.190539	0.2017	0.184545	0.01	0.01	0.01
29	H	0.192326	0.205143	0.185699	0.01	0.01	0.01
30	H	0.235457	0.264347	0.202517	0.03	0.03	0.03
31	H	0.194723	0.203723	0.182634	0.01	0.01	0.01
32	H	0.202431	0.210642	0.190641	0.01	0.01	0.01
33	H	0.180838	0.184378	0.175715	0.01	0.00	0.00
34	H	0.193699	0.197006	0.18851	0.01	0.00	0.00
35	H	0.195248	0.198597	0.190191	0.01	0.00	0.00
36	H	0.181594	0.185132	0.176547	0.01	0.00	0.00
37	H	0.200476	0.224074	0.169616	0.03	0.02	0.03
38	H	0.303402	0.305594	0.300304	0.00	0.00	0.00
39	H	0.423473	0.431609	0.414711	0.01	0.01	0.01
40	O	-0.462981	-0.431452	-0.487809	0.02	0.03	0.03
41	H	0.485584	0.495122	0.479467	0.01	0.01	0.01
42	O	-0.471855	-0.468717	-0.477264	0.01	0.00	0.00
43	H	0.366333	0.368315	0.36508	0.00	0.00	0.00
44	O	-0.46851	-0.462869	-0.477918	0.01	0.01	0.01
45	H	0.358304	0.359535	0.357415	0.00	0.00	0.00

A3

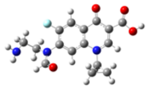

Standard orientation:

Center Number	Atomic Number	Atomic Type	Coordinates (Angstroms) X	Y	Z
1	9	0	-2.491122	-2.369769	-0.326726
2	8	0	2.467829	-2.777007	-0.427297
3	8	0	5.149499	-2.055526	-0.070716
4	8	0	5.511364	0.133010	0.155885
5	7	0	1.658346	1.163660	0.325804
6	7	0	-3.112598	0.277832	0.254244
7	7	0	-6.015720	-0.285726	-0.401807
8	6	0	1.386564	2.567443	0.581675
9	6	0	2.197123	3.598528	-0.130831
10	6	0	0.758285	3.380737	-0.504557
11	6	0	0.597486	0.266916	0.178462
12	6	0	2.914523	0.722967	0.277077
13	6	0	0.871684	-1.080711	-0.081287
14	6	0	-0.721577	0.708613	0.305299
15	6	0	-1.775064	-0.168540	0.131238
16	6	0	-4.072578	-0.446104	1.110199
17	6	0	-3.495907	1.466902	-0.280015
18	6	0	3.272785	-0.587489	0.048242
19	6	0	2.252872	-1.586585	-0.175483
20	6	0	-5.164342	-1.196035	0.366214
21	6	0	-0.194681	-1.967971	-0.241873
22	6	0	-1.477378	-1.508445	-0.147291
23	6	0	4.698376	-0.934028	0.033716
24	1	0	1.084033	2.761573	1.602370
25	1	0	2.943092	3.250579	-0.832422
26	1	0	2.462166	4.480733	0.433734
27	1	0	0.019415	4.109946	-0.203026
28	1	0	0.565369	2.873348	-1.440738
29	1	0	3.671361	1.478600	0.434286
30	1	0	-0.934694	1.737975	0.548025
31	1	0	-4.530791	0.289581	1.776273
32	1	0	-3.505629	-1.134571	1.734349
33	1	0	-4.494696	1.773689	0.045318
34	1	0	-4.726421	-1.914280	-0.323727
35	1	0	-5.716801	-1.762048	1.124814
36	1	0	-0.009814	-3.013417	-0.451320
37	1	0	-6.725020	-0.830323	-0.882202
38	1	0	6.426010	-0.191372	0.149850
39	8	0	-2.827489	2.148363	-1.044366
40	1	0	-6.516327	0.319640	0.244497

A3		q(A)	q(A+1)	q(A-1)	fk+	fk-	fk0
1	F	-0.319334	-0.286513	-0.339705	0.02	0.03	0.03
2	O	-0.683607	-0.496453	-0.840955	0.16	0.19	0.17
3	O	-0.603072	-0.542019	-0.629126	0.03	0.06	0.04
4	O	-0.485755	-0.463586	-0.495791	0.01	0.02	0.02
5	N	0.398914	0.525063	0.327113	0.07	0.13	0.10
6	N	0.276529	0.308339	0.192882	0.08	0.03	0.06
7	N	-0.63419	-0.633803	-0.635039	0.00	0.00	0.00
8	C	-0.355114	-0.38924	-0.300125	0.05	0.03	0.04
9	C	-0.338025	-0.326007	-0.335288	0.00	0.01	0.00
10	C	-0.254102	-0.239241	-0.260062	0.01	0.01	0.01
11	C	-0.076256	-0.059235	-0.057655	0.02	0.02	0.00
12	C	-0.570294	-0.630869	-0.591624	0.02	0.06	0.02
13	C	0.497592	0.637131	0.325711	0.17	0.14	0.16
14	C	-0.237632	-0.183263	-0.367622	0.13	0.05	0.09
15	C	-0.032099	-0.093426	0.007318	0.04	0.06	0.05
16	C	-0.472551	-0.484767	-0.454792	0.02	0.01	0.01
17	C	0.264794	0.268523	0.247257	0.02	0.00	0.01
18	C	0.375377	0.632746	0.282789	0.09	0.26	0.17
19	C	-0.019826	-0.102791	-0.019151	0.00	0.08	0.04
20	C	-0.178019	-0.176561	-0.180552	0.00	0.00	0.00
21	C	-0.288455	-0.243291	-0.433712	0.15	0.05	0.10
22	C	-0.060536	-0.029635	-0.004396	0.06	0.03	0.01
23	C	0.885036	0.904867	0.874849	0.01	0.02	0.02
24	H	0.209001	0.240653	0.191023	0.02	0.03	0.02
25	H	0.178664	0.190321	0.172167	0.01	0.01	0.01
26	H	0.173065	0.182912	0.167277	0.01	0.01	0.01
27	H	0.184535	0.197007	0.178092	0.01	0.01	0.01
28	H	0.192754	0.206406	0.185105	0.01	0.01	0.01
29	H	0.21711	0.242606	0.194114	0.02	0.03	0.02
30	H	0.220885	0.245291	0.184661	0.04	0.02	0.03
31	H	0.222943	0.228263	0.212175	0.01	0.01	0.01
32	H	0.219477	0.224729	0.209524	0.01	0.01	0.01
33	H	0.168252	0.174523	0.1526	0.02	0.01	0.01
34	H	0.170096	0.171174	0.1669	0.00	0.00	0.00
35	H	0.164802	0.167269	0.16059	0.00	0.00	0.00
36	H	0.202839	0.221445	0.172942	0.03	0.02	0.02
37	H	0.284262	0.285369	0.281965	0.00	0.00	0.00
38	H	0.390202	0.399495	0.38435	0.01	0.01	0.01
39	O	-0.563173	-0.549485	-0.598776	0.04	0.01	0.02
40	H	0.274909	0.276048	0.272966	0.00	0.00	0.00

XIV

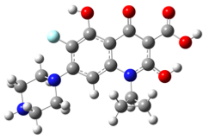

Standard orientation:

Center Number	Atomic Number	Atomic Type	Coordinates (Angstroms) X	Y	Z
1	9	0	-2.326196	-2.673890	-0.592899
2	8	0	2.749926	-2.486478	-0.618893
3	8	0	5.048800	-1.756821	0.688434
4	8	0	5.418668	0.433619	0.592300
5	7	0	1.421110	1.281580	0.073490
6	7	0	-3.236602	-0.100348	-0.104116
7	7	0	-5.785224	0.802939	0.848821
8	6	0	1.004585	2.673621	0.052257
9	6	0	1.679599	3.604929	-0.902357
10	6	0	0.270780	3.167409	-1.156317
11	6	0	0.466274	0.262366	-0.075527
12	6	0	2.714705	0.980653	0.313047
13	6	0	0.896857	-1.058471	-0.265925
14	6	0	-0.884741	0.594215	-0.000533
15	6	0	-1.867901	-0.380079	-0.141654
16	6	0	-3.973793	-0.826329	0.951515
17	6	0	-3.589105	1.322294	-0.080179
18	6	0	3.212487	-0.318023	0.183572
19	6	0	2.329972	-1.379342	-0.266508
20	6	0	-5.464977	-0.624158	0.789806
21	6	0	-5.086424	1.488904	-0.239408
22	6	0	-0.100745	-2.044733	-0.424380
23	6	0	-1.429377	-1.686626	-0.369902
24	6	0	4.600207	-0.647661	0.500859
25	1	0	0.708490	3.058666	1.019268
26	1	0	2.448102	3.181368	-1.534009
27	1	0	1.864479	4.609503	-0.549172
28	1	0	-0.539399	3.861849	-0.981859
29	1	0	0.111409	2.453648	-1.953511
30	1	0	-1.171086	1.611848	0.199677
31	1	0	-3.653574	-0.452029	1.933321
32	1	0	-3.744750	-1.887004	0.898960
33	1	0	-3.080087	1.827297	-0.901814
34	1	0	-3.270483	1.786731	0.863401
35	1	0	-5.782558	-1.073073	-0.161331
36	1	0	-5.978173	-1.145207	1.598059
37	1	0	-5.325044	2.551945	-0.210349
38	1	0	-5.385009	1.099730	-1.222336
39	1	0	-6.783707	0.909667	0.699572
40	1	0	6.288374	0.144128	0.912715
41	8	0	0.222092	-3.342982	-0.635731
42	1	0	-0.591521	-3.867024	-0.673941
43	8	0	3.433848	2.024447	0.682760
44	1	0	4.368227	1.755658	0.790922

XIV		q(A)	q(A+1)	q(A-1)	fk+	fk-	fk0
1	F	-0.357522	-0.324325	-0.372379	0.01	0.03	0.02
2	O	-0.680421	-0.629431	-0.850933	0.17	0.05	0.11
3	O	-0.583858	-0.572206	-0.653723	0.07	0.01	0.04
4	O	-0.519203	-0.515278	-0.546767	0.03	0.00	0.02
5	N	0.176445	0.213249	0.107547	0.07	0.04	0.05
6	N	-0.08642	0.207458	-0.173164	0.09	0.29	0.19
7	N	-0.385308	-0.3783	-0.384081	0.00	0.01	0.00
8	C	-0.291798	-0.31296	-0.254328	0.04	0.02	0.03
9	C	-0.327991	-0.320907	-0.339945	0.01	0.01	0.01
10	C	-0.22299	-0.225294	-0.22908	0.01	0.00	0.00
11	C	0.140784	0.163722	0.095332	0.05	0.02	0.03
12	C	0.156173	0.158565	0.186285	0.03	0.00	0.01
13	C	0.291617	0.418508	0.143917	0.15	0.13	0.14
14	C	-0.522457	-0.480882	-0.524495	0.00	0.04	0.02
15	C	0.362663	0.328494	0.318801	0.04	0.03	0.00
16	C	-0.278463	-0.334426	-0.198246	0.08	0.06	0.07
17	C	-0.082272	-0.120941	-0.066817	0.02	0.04	0.03
18	C	-0.306595	-0.289076	-0.391186	0.08	0.02	0.05
19	C	0.298307	0.286169	0.268407	0.03	0.01	0.01
20	C	-0.294898	-0.284797	-0.335761	0.04	0.01	0.03
21	C	-0.512012	-0.482082	-0.522328	0.01	0.03	0.02
22	C	0.059054	0.049129	-0.004846	0.06	0.01	0.03
23	C	-0.158283	-0.096393	-0.151658	0.01	0.06	0.03
24	C	0.924435	0.926348	0.886828	0.04	0.00	0.02
25	H	0.209972	0.216345	0.199034	0.01	0.01	0.01
26	H	0.186651	0.190384	0.181448	0.01	0.00	0.00
27	H	0.175999	0.179481	0.170856	0.01	0.00	0.00
28	H	0.186775	0.190546	0.181603	0.01	0.00	0.00
29	H	0.194171	0.198204	0.188139	0.01	0.00	0.01
30	H	0.210462	0.244489	0.187471	0.02	0.03	0.03
31	H	0.178649	0.251286	0.165057	0.01	0.07	0.04
32	H	0.189602	0.225796	0.182019	0.01	0.04	0.02
33	H	0.19384	0.233688	0.184027	0.01	0.04	0.02
34	H	0.193674	0.265039	0.181379	0.01	0.07	0.04
35	H	0.164939	0.185356	0.160253	0.00	0.02	0.01
36	H	0.182705	0.199236	0.178241	0.00	0.02	0.01
37	H	0.185673	0.202639	0.181099	0.00	0.02	0.01
38	H	0.167637	0.188374	0.162832	0.00	0.02	0.01
39	H	0.284864	0.29588	0.281937	0.00	0.01	0.01
40	H	0.407953	0.410519	0.396465	0.01	0.00	0.01
41	O	-0.491015	-0.465246	-0.528035	0.04	0.03	0.03
42	H	0.364491	0.370198	0.353855	0.01	0.01	0.01
43	O	-0.4496	-0.433933	-0.473508	0.02	0.02	0.02
44	H	0.463573	0.467377	0.45845	0.01	0.00	0.00

B9

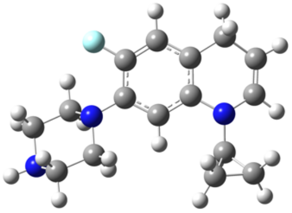

Standard orientation:

Center Number	Atomic Number	Atomic Type	Coordinates (Angstroms) X	Y	Z
1	9	0	-1.651051	-2.807752	-0.505192
2	7	0	2.637181	0.610701	0.105516
3	7	0	-2.208124	-0.114170	-0.259215
4	7	0	-4.600212	1.258528	0.530344
5	6	0	2.387170	1.987057	0.436609
6	6	0	3.359512	3.006929	-0.061983
7	6	0	1.981831	2.923697	-0.658828
8	6	0	1.536252	-0.256104	-0.041884
9	6	0	3.823216	0.048025	0.596021
10	6	0	1.759107	-1.633446	-0.156464
11	6	0	0.230878	0.246404	-0.048758
12	6	0	-0.877439	-0.587482	-0.196860
13	6	0	-3.042812	-0.591447	0.859267
14	6	0	-2.342809	1.337571	-0.390283
15	6	0	4.103867	-1.250405	0.511007
16	6	0	3.162272	-2.190803	-0.186283
17	6	0	-4.489221	-0.197033	0.645765
18	6	0	-3.795962	1.707371	-0.608100
19	6	0	0.656565	-2.469962	-0.281784
20	6	0	-0.616533	-1.952065	-0.315364
21	1	0	1.944879	2.157925	1.414706
22	1	0	4.183971	2.644846	-0.661432
23	1	0	3.587259	3.836786	0.591694
24	1	0	1.265300	3.695518	-0.413396
25	1	0	1.894185	2.500394	-1.650506
26	1	0	4.504307	0.761366	1.042308
27	1	0	0.076728	1.307643	0.065754
28	1	0	-2.676289	-0.158710	1.801540
29	1	0	-2.970419	-1.674224	0.931179
30	1	0	-1.749707	1.672805	-1.242218
31	1	0	-1.974801	1.851808	0.509499
32	1	0	-4.864326	-0.704791	-0.253562
33	1	0	-5.078373	-0.538050	1.497349
34	1	0	-3.874490	2.790742	-0.700791
35	1	0	-4.140521	1.259743	-1.550418
36	1	0	0.789014	-3.541406	-0.384421
37	1	0	-5.571139	1.487534	0.341787
38	1	0	3.176105	-3.179277	0.277042
39	1	0	3.480138	-2.338602	-1.227116
40	1	0	5.040379	-1.617543	0.908750

B9		q(A)	q(A+1)	q(A-1)	fk+	fk-	fk0
1	F	-0.332833	-0.302894	-0.365109	0.03	0.03	0.03
2	N	0.144995	0.347016	0.071156	0.07	0.20	0.14
3	N	-0.136188	-0.089566	-0.211874	0.08	0.05	0.06
4	N	-0.387253	-0.386247	-0.387804	0.00	0.00	0.00
5	C	-0.211187	-0.296593	-0.118713	0.09	0.09	0.09
6	C	-0.347447	-0.343596	-0.327492	0.02	0.00	0.01
7	C	-0.269184	-0.23607	-0.282913	0.01	0.03	0.02
8	C	0.023447	0.06338	0.083521	0.06	0.04	0.01
9	C	-0.153548	-0.074714	-0.171867	0.02	0.08	0.05
10	C	0.561657	0.735513	0.517232	0.04	0.17	0.11
11	C	-0.329443	-0.301205	-0.745892	0.42	0.03	0.22
12	C	0.598508	0.603895	0.508915	0.09	0.01	0.05
13	C	-0.249644	-0.282671	-0.171254	0.08	0.03	0.06
14	C	-0.022625	-0.034849	0.051203	0.07	0.01	0.04
15	C	-0.205066	-0.041973	-0.232555	0.03	0.16	0.10
16	C	-0.73181	-0.836355	-0.627241	0.10	0.10	0.10
17	C	-0.304945	-0.300666	-0.310137	0.01	0.00	0.00
18	C	-0.57392	-0.560049	-0.58714	0.01	0.01	0.01
19	C	-0.235414	-0.253473	-0.816	0.58	0.02	0.28
20	C	-0.501052	-0.441901	-0.260663	0.24	0.06	0.09
21	H	0.183715	0.230184	0.165609	0.02	0.05	0.03
22	H	0.166634	0.18032	0.160529	0.01	0.01	0.01
23	H	0.164821	0.176469	0.15993	0.00	0.01	0.01
24	H	0.17306	0.187018	0.168649	0.00	0.01	0.01
25	H	0.179182	0.194475	0.175636	0.00	0.02	0.01
26	H	0.176968	0.211663	0.166839	0.01	0.03	0.02
27	H	0.196622	0.221704	0.143859	0.05	0.03	0.04
28	H	0.171621	0.179741	0.157864	0.01	0.01	0.01
29	H	0.184091	0.188933	0.173106	0.01	0.00	0.01
30	H	0.189603	0.19621	0.17694	0.01	0.01	0.01
31	H	0.185395	0.19349	0.172376	0.01	0.01	0.01
32	H	0.161864	0.165001	0.1564	0.01	0.00	0.00
33	H	0.180185	0.183104	0.175234	0.00	0.00	0.00
34	H	0.182991	0.185879	0.177936	0.01	0.00	0.00
35	H	0.164067	0.167254	0.158734	0.01	0.00	0.00
36	H	0.181212	0.198918	0.147182	0.03	0.02	0.03
37	H	0.28179	0.283802	0.278069	0.00	0.00	0.00
38	H	0.185596	0.227927	0.157032	0.03	0.04	0.04
39	H	0.196795	0.26008	0.165678	0.03	0.06	0.05
40	H	0.156738	0.200843	0.147029	0.01	0.04	0.03

A18

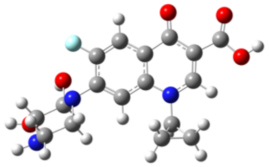

Standard orientation:

Center Number	Atomic Number	Atomic Type	X	Y	Z
1	9	0	-1.695623	-2.696766	-1.024502
2	8	0	3.232549	-2.607615	-0.412358
3	8	0	5.775380	-1.608720	0.181652
4	8	0	5.852761	0.566998	0.664270
5	7	0	1.937044	1.215834	0.257937
6	7	0	-2.687741	-0.178013	-0.532376
7	7	0	-5.175507	1.109699	0.025125
8	6	0	1.488290	2.573215	0.513987
9	6	0	2.292694	3.714819	-0.012497
10	6	0	0.964029	3.381702	-0.629485
11	6	0	1.001970	0.223568	-0.050738
12	6	0	3.225921	0.903012	0.377667
13	6	0	1.443469	-1.082913	-0.278530
14	6	0	-0.363127	0.529128	-0.114960
15	6	0	-1.292057	-0.444498	-0.427494
16	6	0	-3.383082	-0.390333	0.736671
17	6	0	-2.999652	1.167739	-1.042847
18	6	0	3.744008	-0.357115	0.172521
19	6	0	2.867726	-1.447004	-0.190685
20	6	0	-4.885133	-0.215696	0.544612
21	6	0	-4.490592	1.290253	-1.259600
22	6	0	0.503979	-2.068492	-0.599419
23	6	0	-0.819850	-1.743105	-0.675295
24	6	0	5.189103	-0.556542	0.328567
25	1	0	1.005927	2.683522	1.476437
26	1	0	3.174966	3.483199	-0.593522
27	1	0	2.367521	4.585114	0.623268
28	1	0	0.115170	4.019984	-0.427884
29	1	0	0.981179	2.911215	-1.603679
30	1	0	3.872082	1.723591	0.654992
31	1	0	-0.698674	1.534383	0.088929
32	1	0	-3.036544	0.335876	1.489664
33	1	0	-2.466352	1.305269	-1.982999
34	1	0	-2.682846	1.951347	-0.343533
35	1	0	-5.231353	-1.011920	-0.127578
36	1	0	-4.716078	2.284452	-1.642889
37	1	0	-4.813683	0.553904	-2.005837
38	1	0	0.827118	-3.081955	-0.798471
39	1	0	-6.179180	1.157832	-0.132453
40	1	0	6.792889	0.342518	0.751588
41	8	0	-3.102633	-1.698779	1.174370
42	1	0	-3.450467	-1.780004	2.071814
43	8	0	-5.468780	-0.387378	1.818099
44	1	0	-6.358021	-0.742540	1.704754

A18		q(A)	q(A+1)	q(A-1)	fk+	fk-	fk0
1	F	-0.310235	-0.270798	-0.331873	0.02	0.04	0.03
2	O	-0.688501	-0.505459	-0.857699	0.17	0.18	0.18
3	O	-0.606298	-0.550429	-0.632776	0.03	0.06	0.04
4	O	-0.487084	-0.466733	-0.496973	0.01	0.02	0.02
5	N	0.398717	0.518375	0.326406	0.07	0.12	0.10
6	N	-0.148024	-0.108732	-0.225921	0.08	0.04	0.06
7	N	-0.439763	-0.437996	-0.442726	0.00	0.00	0.00
8	C	-0.353054	-0.389851	-0.291003	0.06	0.04	0.05
9	C	-0.339908	-0.331133	-0.336662	0.00	0.01	0.00
10	C	-0.251794	-0.233886	-0.262208	0.01	0.02	0.01
11	C	-0.106648	-0.085361	-0.073179	0.03	0.02	0.01
12	C	-0.575753	-0.637399	-0.621216	0.05	0.06	0.01
13	C	0.446172	0.565124	0.322098	0.12	0.12	0.12
14	C	-0.209377	-0.157075	-0.346977	0.14	0.05	0.09
15	C	-0.287752	-0.314039	-0.316128	0.03	0.03	0.00
16	C	-0.041531	-0.055672	0.002268	0.04	0.01	0.03
17	C	-0.027156	-0.036614	-0.008524	0.02	0.01	0.01
18	C	0.309041	0.545079	0.229348	0.08	0.24	0.16
19	C	0.082178	0.006435	0.073323	0.01	0.08	0.03
20	C	0.153136	0.150767	0.146215	0.01	0.00	0.00
21	C	-0.547302	-0.539451	-0.556634	0.01	0.01	0.01
22	C	-0.001056	0.060797	-0.151402	0.15	0.06	0.11
23	C	0.084022	0.118637	0.115938	0.03	0.03	0.00
24	C	0.903723	0.92267	0.89258	0.01	0.02	0.02
25	H	0.209083	0.238852	0.189	0.02	0.03	0.02
26	H	0.177815	0.188917	0.170655	0.01	0.01	0.01
27	H	0.173402	0.182809	0.167478	0.01	0.01	0.01
28	H	0.183291	0.195233	0.176351	0.01	0.01	0.01
29	H	0.191828	0.204542	0.185049	0.01	0.01	0.01
30	H	0.214958	0.239013	0.189877	0.03	0.02	0.02
31	H	0.21122	0.23639	0.17757	0.03	0.03	0.03
32	H	0.189296	0.196525	0.177051	0.01	0.01	0.01
33	H	0.201028	0.206198	0.191969	0.01	0.01	0.01
34	H	0.192473	0.198437	0.18277	0.01	0.01	0.01
35	H	0.182891	0.185582	0.17831	0.00	0.00	0.00
36	H	0.191727	0.194342	0.18717	0.00	0.00	0.00
37	H	0.175938	0.178736	0.17124	0.00	0.00	0.00
38	H	0.199481	0.220031	0.170107	0.03	0.02	0.02
39	H	0.292038	0.29384	0.289175	0.00	0.00	0.00
40	H	0.39026	0.398776	0.384106	0.01	0.01	0.01
41	O	-0.488191	-0.487252	-0.489429	0.00	0.00	0.00
42	H	0.352519	0.355765	0.346964	0.01	0.00	0.00
43	O	-0.549722	-0.547848	-0.552658	0.00	0.00	0.00
44	H	0.352916	0.353852	0.350971	0.00	0.00	0.00

B14

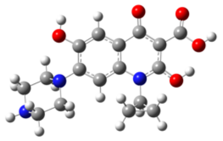

Standard orientation:

Center Number	Atomic Number	Atomic Type	Coordinates (Angstroms) X	Y	Z
1	8	0	2.646172	-2.701457	-0.524053
2	8	0	5.090125	-1.939239	0.433890
3	8	0	5.448343	0.252897	0.446397
4	7	0	1.440038	1.143020	0.124314
5	7	0	-3.229331	-0.249972	-0.151454
6	7	0	-5.785212	0.664334	0.771902
7	6	0	1.027784	2.534677	0.192427
8	6	0	1.668527	3.523437	-0.727544
9	6	0	0.253229	3.096868	-0.960445
10	6	0	0.475993	0.136599	-0.055657
11	6	0	2.736473	0.820013	0.291442
12	6	0	0.883551	-1.174256	-0.270981
13	6	0	-0.887685	0.440984	0.012007
14	6	0	-1.854200	-0.535297	-0.178720
15	6	0	-3.956439	-0.940607	0.932731
16	6	0	-3.585009	1.170504	-0.156832
17	6	0	3.220583	-0.488598	0.120602
18	6	0	2.302190	-1.541862	-0.252206
19	6	0	-5.449789	-0.761063	0.762743
20	6	0	-5.082682	1.326488	-0.329327
21	6	0	-0.087965	-2.160753	-0.483310
22	6	0	-1.424945	-1.858478	-0.459868
23	6	0	4.625706	-0.824396	0.340733
24	1	0	0.763524	2.862287	1.189321
25	1	0	2.415723	3.141813	-1.409317
26	1	0	1.863179	4.505796	-0.320899
27	1	0	-0.553262	3.775186	-0.718503
28	1	0	0.070080	2.431228	-1.793300
29	1	0	-1.192558	1.449049	0.238343
30	1	0	-3.641924	-0.521838	1.899198
31	1	0	-3.710340	-1.998572	0.921424
32	1	0	-3.069977	1.662928	-0.982859
33	1	0	-3.278632	1.657238	0.780246
34	1	0	-5.759553	-1.246196	-0.173225
35	1	0	-5.961351	-1.258845	1.586707
36	1	0	-5.328651	2.388408	-0.327974
37	1	0	-5.372632	0.911107	-1.304086
38	1	0	0.228992	-3.177031	-0.686378
39	1	0	-6.783150	0.756224	0.609899
40	1	0	6.340610	-0.053836	0.675325
41	8	0	-2.382772	-2.795386	-0.732228
42	1	0	-1.954347	-3.641500	-0.919405
43	8	0	3.489359	1.846469	0.645013
44	1	0	4.424068	1.563040	0.703233

B14		q(A)	q(A+1)	q(A-1)	fk+	fk-	fk0
1	O	-0.7109	-0.645897	-0.882248	0.17	0.07	0.12
2	O	-0.594852	-0.582994	-0.656689	0.06	0.01	0.04
3	O	-0.529968	-0.526006	-0.553943	0.02	0.00	0.01
4	N	0.159815	0.200092	0.09374	0.07	0.04	0.05
5	N	-0.081282	0.146646	-0.162885	0.08	0.23	0.15
6	N	-0.384669	-0.379712	-0.38487	0.00	0.00	0.00
7	C	-0.282264	-0.301884	-0.24716	0.04	0.02	0.03
8	C	-0.358352	-0.351655	-0.370057	0.01	0.01	0.01
9	C	-0.206389	-0.207283	-0.209595	0.00	0.00	0.00
10	C	0.040026	0.076673	0.01808	0.02	0.04	0.03
11	C	0.219663	0.223677	0.238254	0.02	0.00	0.01
12	C	0.381967	0.53102	0.237815	0.14	0.15	0.15
13	C	-0.471711	-0.469797	-0.486072	0.01	0.00	0.01
14	C	0.289605	0.277306	0.224	0.07	0.01	0.03
15	C	-0.310087	-0.371255	-0.236311	0.07	0.06	0.07
16	C	-0.026078	-0.057692	-0.014683	0.01	0.03	0.02
17	C	-0.354296	-0.326836	-0.431196	0.08	0.03	0.05
18	C	0.285391	0.28724	0.240127	0.05	0.00	0.02
19	C	-0.273852	-0.264689	-0.29852	0.02	0.01	0.02
20	C	-0.564534	-0.521489	-0.571415	0.01	0.04	0.02
21	C	-0.417674	-0.388522	-0.540487	0.12	0.03	0.08
22	C	-0.239908	-0.220016	-0.175766	0.06	0.02	0.02
23	C	1.073149	1.079566	1.032006	0.04	0.01	0.02
24	H	0.209237	0.21703	0.198106	0.01	0.01	0.01
25	H	0.186544	0.191063	0.181408	0.01	0.00	0.00
26	H	0.177043	0.181022	0.171928	0.01	0.00	0.00
27	H	0.186652	0.190924	0.181388	0.01	0.00	0.00
28	H	0.19438	0.198952	0.188143	0.01	0.00	0.01
29	H	0.208557	0.236685	0.182898	0.03	0.03	0.03
30	H	0.17412	0.225927	0.160789	0.01	0.05	0.03
31	H	0.197267	0.223336	0.188514	0.01	0.03	0.02
32	H	0.191681	0.220983	0.182306	0.01	0.03	0.02
33	H	0.192646	0.24275	0.180332	0.01	0.05	0.03
34	H	0.162758	0.177474	0.158085	0.00	0.01	0.01
35	H	0.180536	0.192985	0.17603	0.00	0.01	0.01
36	H	0.18437	0.197425	0.179754	0.00	0.01	0.01
37	H	0.167289	0.182658	0.162356	0.00	0.02	0.01
38	H	0.187913	0.211807	0.160492	0.03	0.02	0.03
39	H	0.283494	0.291774	0.280612	0.00	0.01	0.01
40	H	0.412299	0.415179	0.40177	0.01	0.00	0.01
41	O	-0.51341	-0.426356	-0.53635	0.02	0.09	0.05
42	H	0.365478	0.385177	0.359739	0.01	0.02	0.01
43	O	-0.463477	-0.440574	-0.486981	0.02	0.02	0.02
44	H	0.471822	0.477287	0.466557	0.01	0.01	0.01

A21

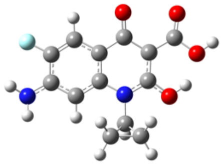

Standard orientation:

Center Number	Atomic Number	Atomic Type	Coordinates (Angstroms) X	Y	Z
1	9	0	4.054974	-2.080596	0.116600
2	8	0	-0.868981	-2.800611	0.562093
3	8	0	-3.495850	-2.456664	-0.078678
4	8	0	-4.191436	-0.350101	-0.189097
5	7	0	-0.368200	1.176835	-0.195876
6	7	0	4.424145	0.520292	-0.513409
7	6	0	-0.198974	2.616818	-0.289794
8	6	0	-0.909182	3.491674	0.692569
9	6	0	0.571963	3.307488	0.793331
10	6	0	0.760754	0.345426	-0.130515
11	6	0	-1.607773	0.644309	-0.237447
12	6	0	0.587410	-1.016612	0.125246
13	6	0	2.036572	0.874244	-0.329376
14	6	0	3.157810	0.057323	-0.255160
15	6	0	-1.857040	-0.720551	-0.018803
16	6	0	-0.744722	-1.605683	0.257343
17	6	0	1.719170	-1.838870	0.217391
18	6	0	2.954356	-1.301645	0.034951
19	6	0	-3.204511	-1.280742	-0.092348
20	1	0	-0.084056	2.977480	-1.303489
21	1	0	-1.517860	2.995950	1.435718
22	1	0	-1.301157	4.424938	0.313468
23	1	0	1.228519	4.109191	0.485174
24	1	0	0.937259	2.688454	1.601862
25	1	0	2.180071	1.920279	-0.557930
26	1	0	1.608888	-2.895600	0.423351
27	1	0	-5.042662	-0.801159	-0.309134
28	8	0	-2.543716	1.531850	-0.512975
29	1	0	-3.421505	1.098499	-0.491462
30	1	0	4.550003	1.518399	-0.411975
31	1	0	5.180986	-0.025837	-0.125058

A21		q(A)	q(A+1)	q(A-1)	fk+	fk-	fk0
1	F	-0.335326	-0.288919	-0.356965	0.02	0.05	0.03
2	O	-0.707018	-0.627748	-0.882106	0.18	0.08	0.13
3	O	-0.599953	-0.584292	-0.652984	0.05	0.02	0.03
4	O	-0.549384	-0.544382	-0.569487	0.02	0.01	0.01
5	N	0.178669	0.227461	0.113864	0.06	0.05	0.06
6	N	-0.58244	-0.368121	-0.646397	0.06	0.21	0.14
7	C	-0.340627	-0.362159	-0.2975	0.04	0.02	0.03
8	C	-0.304706	-0.301117	-0.308079	0.00	0.00	0.00
9	C	-0.221059	-0.221975	-0.23363	0.01	0.00	0.01
10	C	0.057933	0.142987	0.013776	0.04	0.09	0.06
11	C	0.210168	0.216239	0.219171	0.01	0.01	0.00
12	C	0.327484	0.512123	0.214923	0.11	0.18	0.15
13	C	0.02247	0.086408	-0.035422	0.06	0.06	0.06
14	C	0.213944	0.156008	0.195577	0.02	0.06	0.02
15	C	-0.178329	-0.139683	-0.275134	0.10	0.04	0.07
16	C	0.051918	0.022609	0.045178	0.01	0.03	0.01
17	C	-0.170263	-0.161668	-0.334536	0.16	0.01	0.09
18	C	-0.401443	-0.312173	-0.355109	0.05	0.09	0.02
19	C	0.958562	0.959478	0.93685	0.02	0.00	0.01
20	H	0.208713	0.217032	0.197221	0.01	0.01	0.01
21	H	0.185375	0.190183	0.180182	0.01	0.00	0.01
22	H	0.17496	0.179369	0.169764	0.01	0.00	0.00
23	H	0.181685	0.18641	0.176721	0.00	0.00	0.00
24	H	0.187542	0.19344	0.181277	0.01	0.01	0.01
25	H	0.19879	0.233484	0.17612	0.02	0.03	0.03
26	H	0.198608	0.22336	0.168904	0.03	0.02	0.03
27	H	0.417459	0.42096	0.408076	0.01	0.00	0.01
28	O	-0.477424	-0.453983	-0.502594	0.03	0.02	0.02
29	H	0.481914	0.487763	0.476014	0.01	0.01	0.01
30	H	0.306488	0.357388	0.287651	0.02	0.05	0.03
31	H	0.305289	0.35352	0.288672	0.02	0.05	0.03

A5

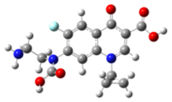

Standard orientation:

Center Number	Atomic Number	Atomic Type	Coordinates (Angstroms) X	Y	Z
1	9	0	-2.214552	-2.488816	-0.466940
2	8	0	2.757515	-2.735239	-0.474627
3	8	0	5.405080	-1.938191	-0.029823
4	8	0	5.691130	0.256124	0.246209
5	7	0	1.804276	1.160929	0.336155
6	7	0	-2.935021	0.097671	0.205109
7	7	0	-5.674755	-0.949901	-0.327276
8	6	0	1.482046	2.552656	0.598436
9	6	0	2.267466	3.613831	-0.097909
10	6	0	0.841848	3.350958	-0.491905
11	6	0	0.776527	0.232241	0.158665
12	6	0	3.075017	0.762405	0.305557
13	6	0	1.099514	-1.100103	-0.124404
14	6	0	-0.558975	0.623539	0.280320
15	6	0	-1.575881	-0.286684	0.072495
16	6	0	-3.774588	-0.555556	1.231227
17	6	0	-3.327918	1.248645	-0.411285
18	6	0	3.480479	-0.532068	0.064975
19	6	0	2.498942	-1.558463	-0.201705
20	6	0	-4.823948	-1.528307	0.713883
21	6	0	0.068176	-2.019374	-0.326947
22	6	0	-1.231197	-1.605209	-0.241247
23	6	0	4.916270	-0.833409	0.082148
24	1	0	1.159589	2.729787	1.616066
25	1	0	3.033731	3.293585	-0.790723
26	1	0	2.496645	4.501334	0.473930
27	1	0	0.075629	4.053887	-0.196691
28	1	0	0.678072	2.842769	-1.433214
29	1	0	3.803449	1.540131	0.487446
30	1	0	-0.815335	1.640525	0.538644
31	1	0	-4.253102	0.231368	1.813298
32	1	0	-3.100980	-1.087800	1.902901
33	1	0	-4.346534	-2.414812	0.302929
34	1	0	-5.385231	-1.846528	1.600318
35	1	0	0.293813	-3.051393	-0.561735
36	1	0	-6.386419	-1.631358	-0.571907
37	1	0	6.615763	-0.038412	0.261959
38	8	0	-2.700863	1.810596	-1.294510
39	1	0	-6.167122	-0.146247	0.052242
40	8	0	-4.490052	1.722371	0.053540
41	1	0	-4.717650	2.505709	-0.471073

A5		q(A)	q(A+1)	q(A-1)	fk+	fk-	fk0
1	F	-0.317504	-0.284854	-0.338172	0.02	0.03	0.03
2	O	-0.683056	-0.497502	-0.843521	0.16	0.19	0.17
3	O	-0.607269	-0.546307	-0.633052	0.03	0.06	0.04
4	O	-0.485248	-0.462546	-0.495528	0.01	0.02	0.02
5	N	0.397693	0.523494	0.324973	0.07	0.13	0.10
6	N	0.124295	0.158236	0.036937	0.09	0.03	0.06
7	N	-0.6381	-0.637896	-0.638293	0.00	0.00	0.00
8	C	-0.355669	-0.392851	-0.300652	0.06	0.04	0.05
9	C	-0.327743	-0.316304	-0.323202	0.00	0.01	0.00
10	C	-0.250115	-0.234764	-0.257682	0.01	0.02	0.01
11	C	-0.053934	-0.034358	-0.043821	0.01	0.02	0.00
12	C	-0.238512	-0.244047	-0.306055	0.07	0.01	0.03
13	C	0.24159	0.325528	0.158727	0.08	0.08	0.08
14	C	-0.252819	-0.193701	-0.387464	0.13	0.06	0.10
15	C	-0.176751	-0.254138	-0.115553	0.06	0.08	0.07
16	C	-0.382384	-0.3943	-0.358909	0.02	0.01	0.02
17	C	0.605116	0.608548	0.597923	0.01	0.00	0.01
18	C	0.063625	0.272087	0.003744	0.06	0.21	0.13
19	C	0.087203	0.019199	0.065701	0.02	0.07	0.02
20	C	-0.27154	-0.270331	-0.272352	0.00	0.00	0.00
21	C	-0.240719	-0.184002	-0.394478	0.15	0.06	0.11
22	C	0.211402	0.274244	0.198314	0.01	0.06	0.04
23	C	0.897556	0.921233	0.885158	0.01	0.02	0.02
24	H	0.20894	0.240827	0.190748	0.02	0.03	0.03
25	H	0.178819	0.190594	0.172084	0.01	0.01	0.01
26	H	0.172479	0.182388	0.166578	0.01	0.01	0.01
27	H	0.185108	0.197527	0.178637	0.01	0.01	0.01
28	H	0.192139	0.205666	0.184487	0.01	0.01	0.01
29	H	0.217847	0.243561	0.193843	0.02	0.03	0.02
30	H	0.229518	0.254655	0.192958	0.04	0.03	0.03
31	H	0.216465	0.221622	0.204692	0.01	0.01	0.01
32	H	0.221137	0.226141	0.211421	0.01	0.01	0.01
33	H	0.168576	0.169331	0.165543	0.00	0.00	0.00
34	H	0.164868	0.167354	0.160727	0.00	0.00	0.00
35	H	0.203103	0.221646	0.172752	0.03	0.02	0.02
36	H	0.292848	0.293962	0.29073	0.00	0.00	0.00
37	H	0.390686	0.400028	0.38477	0.01	0.01	0.01
38	O	-0.589127	-0.57801	-0.613238	0.02	0.01	0.02
39	H	0.269709	0.27071	0.268077	0.00	0.00	0.00
40	O	-0.467509	-0.462075	-0.480274	0.01	0.01	0.01
41	H	0.397275	0.399405	0.39272	0.00	0.00	0.00

B10

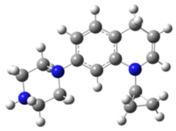

Standard orientation:

Center Number	Atomic Number	Atomic Type	X	Y	Z
1	7	0	2.524795	0.552318	0.107975
2	7	0	-2.273020	-0.323411	-0.315922
3	7	0	-4.773495	0.821479	0.529094
4	6	0	2.228544	1.914780	0.457216
5	6	0	3.173833	2.971689	-0.016351
6	6	0	1.804661	2.855728	-0.627183
7	6	0	1.459477	-0.349721	-0.070864
8	6	0	3.730354	0.024136	0.591088
9	6	0	1.736684	-1.714122	-0.217877
10	6	0	0.135675	0.106965	-0.072852
11	6	0	-0.932517	-0.775367	-0.245132
12	6	0	-3.185606	-1.029057	0.599513
13	6	0	-2.461293	1.122488	-0.199441
14	6	0	4.062925	-1.259216	0.471656
15	6	0	3.162000	-2.212342	-0.261288
16	6	0	-4.623618	-0.623699	0.354686
17	6	0	-3.909647	1.496725	-0.439918
18	6	0	0.660235	-2.582914	-0.354309
19	6	0	-0.651619	-2.141663	-0.379660
20	1	0	1.773334	2.057384	1.433741
21	1	0	4.014301	2.644310	-0.613627
22	1	0	3.370511	3.797198	0.652710
23	1	0	1.062257	3.601114	-0.376936
24	1	0	1.739175	2.445397	-1.625946
25	1	0	4.378368	0.750820	1.064244
26	1	0	-0.047223	1.159090	0.071608
27	1	0	-2.912363	-0.802940	1.641022
28	1	0	-3.095152	-2.102268	0.452271
29	1	0	-1.843908	1.623638	-0.945465
30	1	0	-2.153211	1.480146	0.794646
31	1	0	-4.914128	-0.940869	-0.656588
32	1	0	-5.262025	-1.144528	1.068572
33	1	0	-4.015412	2.576143	-0.329512
34	1	0	-4.177643	1.233407	-1.472571
35	1	0	0.862188	-3.643416	-0.465573
36	1	0	-5.736159	1.068005	0.320881
37	1	0	3.216487	-3.214947	0.167898
38	1	0	3.492951	-2.310964	-1.304154
39	1	0	5.010027	-1.600733	0.867437
40	1	0	-1.450870	-2.855155	-0.528385

B10		q(A)	q(A+1)	q(A-1)	fk+	fk-	fk0
1	N	0.143339	0.336965	0.058765	0.08	0.19	0.14
2	N	-0.122354	-0.072351	-0.207903	0.09	0.05	0.07
3	N	-0.390197	-0.389735	-0.39106	0.00	0.00	0.00
4	C	-0.227306	-0.302927	-0.148825	0.08	0.08	0.08
5	C	-0.342243	-0.332918	-0.308576	0.03	0.01	0.01
6	C	-0.26949	-0.241684	-0.295067	0.03	0.03	0.03
7	C	0.283056	0.324188	0.231583	0.05	0.04	0.05
8	C	-0.151055	-0.076365	-0.156969	0.01	0.07	0.04
9	C	0.570645	0.761955	0.275135	0.30	0.19	0.24
10	C	-0.494998	-0.479296	-0.781735	0.29	0.02	0.15
11	C	0.294539	0.333564	0.303757	0.01	0.04	0.01
12	C	-0.209879	-0.244616	-0.149021	0.06	0.03	0.05
13	C	-0.085988	-0.098588	-0.006316	0.08	0.01	0.05
14	C	-0.203671	-0.049355	-0.266197	0.06	0.15	0.11
15	C	-0.72202	-0.8264	-0.613136	0.11	0.10	0.11
16	C	-0.336393	-0.329831	-0.344278	0.01	0.01	0.01
17	C	-0.522456	-0.513539	-0.512618	0.01	0.01	0.00
18	C	-0.684635	-0.738746	-0.823535	0.14	0.05	0.04
19	C	-0.297823	-0.224968	-0.335615	0.04	0.07	0.06
20	H	0.183184	0.227383	0.168097	0.02	0.04	0.03
21	H	0.165929	0.179118	0.158555	0.01	0.01	0.01
22	H	0.164246	0.175418	0.159063	0.01	0.01	0.01
23	H	0.172215	0.185641	0.16775	0.00	0.01	0.01
24	H	0.177111	0.191696	0.174224	0.00	0.01	0.01
25	H	0.176198	0.209385	0.161866	0.01	0.03	0.02
26	H	0.186926	0.210683	0.141121	0.05	0.02	0.03
27	H	0.16861	0.177907	0.158705	0.01	0.01	0.01
28	H	0.182696	0.18863	0.173612	0.01	0.01	0.01
29	H	0.184038	0.190992	0.170785	0.01	0.01	0.01
30	H	0.182949	0.193074	0.170209	0.01	0.01	0.01
31	H	0.161359	0.164603	0.157473	0.00	0.00	0.00
32	H	0.179915	0.183006	0.176153	0.00	0.00	0.00
33	H	0.181993	0.185057	0.178304	0.00	0.00	0.00
34	H	0.163366	0.166704	0.15896	0.00	0.00	0.00
35	H	0.164176	0.182725	0.124348	0.04	0.02	0.03
36	H	0.282508	0.284662	0.27924	0.00	0.00	0.00
37	H	0.181507	0.224042	0.158613	0.02	0.04	0.03
38	H	0.192408	0.257318	0.170303	0.02	0.06	0.04
39	H	0.155824	0.197705	0.142565	0.01	0.04	0.03
40	H	0.161768	0.188896	0.121665	0.04	0.03	0.03

B6

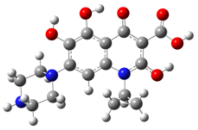

Standard orientation:

Center Number	Atomic Number	Atomic Type	Coordinates (Angstroms) X	Y	Z
1	8	0	-2.642345	-2.558940	0.384442
2	8	0	-5.081195	-1.753190	-0.517878
3	8	0	-5.431171	0.440305	-0.444071
4	7	0	-1.417764	1.304151	-0.115553
5	7	0	3.242512	-0.121192	0.117896
6	7	0	5.815079	0.787557	-0.760835
7	6	0	-1.000633	2.696514	-0.145053
8	6	0	-1.641183	3.663016	0.798344
9	6	0	-0.227882	3.226364	1.023878
10	6	0	-0.451566	0.293901	0.028826
11	6	0	-2.715472	0.993890	-0.292281
12	6	0	-0.879686	-1.025266	0.201108
13	6	0	0.903933	0.597808	-0.025391
14	6	0	1.866992	-0.399738	0.134552
15	6	0	3.973430	-0.796209	-0.973343
16	6	0	3.611766	1.295336	0.161017
17	6	0	-3.206718	-0.318391	-0.163938
18	6	0	-2.288194	-1.375357	0.160995
19	6	0	5.466656	-0.634374	-0.786460
20	6	0	5.109573	1.431110	0.349431
21	6	0	0.096455	-2.024550	0.381623
22	6	0	1.443957	-1.719718	0.365166
23	6	0	-4.615098	-0.643690	-0.386716
24	1	0	-0.732354	3.047494	-1.132827
25	1	0	-2.390407	3.266096	1.468968
26	1	0	-1.833151	4.655570	0.416030
27	1	0	0.581699	3.907600	0.801160
28	1	0	-0.049469	2.539225	1.839978
29	1	0	1.216494	1.610036	-0.214898
30	1	0	3.671822	-0.354893	-1.933752
31	1	0	3.718173	-1.851939	-0.985930
32	1	0	3.096510	1.772708	0.995434
33	1	0	3.317664	1.807783	-0.765981
34	1	0	5.763510	-1.143308	0.140983
35	1	0	5.980489	-1.118656	-1.617023
36	1	0	5.366416	2.490139	0.375027
37	1	0	5.386407	0.989851	1.316612
38	1	0	6.812423	0.866656	-0.588846
39	1	0	-6.328199	0.149772	-0.676114
40	8	0	-3.464738	2.031149	-0.612106
41	1	0	-4.401206	1.753969	-0.677460
42	8	0	2.363858	-2.702331	0.613129
43	1	0	1.886943	-3.527963	0.782747
44	8	0	-0.241933	-3.317946	0.600029
45	1	0	-1.240351	-3.329984	0.586429

B6		q(A)	q(A+1)	q(A-1)	fk+	fk-	fk0
1	O	-0.744421	-0.6978	-0.9028	0.16	0.05	0.10
2	O	-0.5895	-0.57356	-0.664692	0.08	0.02	0.05
3	O	-0.527883	-0.522565	-0.556968	0.03	0.01	0.02
4	N	0.155988	0.209235	0.093265	0.06	0.05	0.06
5	N	-0.065936	0.036057	-0.152521	0.09	0.10	0.09
6	N	-0.382664	-0.379793	-0.382223	0.00	0.00	0.00
7	C	-0.272255	-0.298628	-0.240066	0.03	0.03	0.03
8	C	-0.362287	-0.357254	-0.374295	0.01	0.01	0.01
9	C	-0.20445	-0.20374	-0.207884	0.00	0.00	0.00
10	C	0.124791	0.191292	0.080158	0.04	0.07	0.06
11	C	0.101625	0.100132	0.153441	0.05	0.00	0.03
12	C	0.351465	0.452215	0.207003	0.14	0.10	0.12
13	C	-0.497302	-0.482999	-0.505235	0.01	0.01	0.01
14	C	0.198601	0.237116	0.182168	0.02	0.04	0.03
15	C	-0.304352	-0.364103	-0.230927	0.07	0.06	0.07
16	C	-0.027225	-0.036562	-0.014654	0.01	0.01	0.01
17	C	-0.365203	-0.324697	-0.460792	0.10	0.04	0.07
18	C	0.154535	0.15332	0.111202	0.04	0.00	0.02
19	C	-0.272436	-0.266158	-0.30855	0.04	0.01	0.02
20	C	-0.566647	-0.545119	-0.573533	0.01	0.02	0.01
21	C	0.202486	0.241894	0.136204	0.07	0.04	0.05
22	C	-0.103367	-0.058373	-0.102127	0.00	0.04	0.02
23	C	1.044287	1.051514	0.999236	0.05	0.01	0.03
24	H	0.210505	0.221944	0.199289	0.01	0.01	0.01
25	H	0.187564	0.193654	0.18232	0.01	0.01	0.01
26	H	0.177349	0.182767	0.172281	0.01	0.01	0.01
27	H	0.187012	0.192601	0.181755	0.01	0.01	0.01
28	H	0.195166	0.201493	0.189193	0.01	0.01	0.01
29	H	0.206503	0.234536	0.184535	0.02	0.03	0.03
30	H	0.174394	0.197515	0.162124	0.01	0.02	0.02
31	H	0.197564	0.208746	0.190372	0.01	0.01	0.01
32	H	0.19128	0.206245	0.182294	0.01	0.01	0.01
33	H	0.192834	0.215297	0.181155	0.01	0.02	0.02
34	H	0.162567	0.169614	0.158279	0.00	0.01	0.01
35	H	0.180681	0.18723	0.176504	0.00	0.01	0.01
36	H	0.184471	0.191243	0.180145	0.00	0.01	0.01
37	H	0.167483	0.174847	0.162793	0.00	0.01	0.01
38	H	0.283538	0.287885	0.280815	0.00	0.00	0.00
39	H	0.414039	0.417525	0.40176	0.01	0.00	0.01
40	O	-0.4541	-0.429204	-0.479461	0.03	0.02	0.03
41	H	0.473442	0.479858	0.467923	0.01	0.01	0.01
42	O	-0.551246	-0.402758	-0.570475	0.02	0.15	0.08
43	H	0.383842	0.411433	0.378228	0.01	0.03	0.02
44	O	-0.693285	-0.605412	-0.73321	0.04	0.09	0.06
45	H	0.480547	0.501517	0.465971	0.01	0.02	0.02

B7

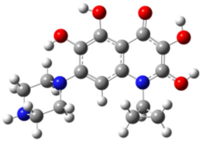

Standard orientation:

Center Number	Atomic Number	Atomic Type	X	Y	Z
1	8	0	3.121051	-2.738944	-0.093746
2	7	0	1.898579	1.160841	0.213882
3	7	0	-2.805819	-0.056129	-0.152282
4	7	0	-5.336925	1.020255	0.632698
5	6	0	1.554989	2.569452	0.238016
6	6	0	2.280362	3.504187	-0.678374
7	6	0	0.852178	3.153238	-0.950250
8	6	0	0.915192	0.186109	0.043214
9	6	0	3.191091	0.785631	0.388310
10	6	0	1.280513	-1.171363	-0.100524
11	6	0	-0.432303	0.569608	0.048682
12	6	0	-1.430716	-0.369473	-0.127295
13	6	0	-3.530345	-0.564746	1.032011
14	6	0	-3.131959	1.357911	-0.356400
15	6	0	3.585782	-0.515375	0.272605
16	6	0	2.678500	-1.565637	0.000723
17	6	0	-5.021451	-0.395389	0.834459
18	6	0	-4.627474	1.511228	-0.551224
19	6	0	0.243485	-2.107831	-0.318183
20	6	0	-1.074927	-1.711408	-0.340741
21	1	0	1.274332	2.935190	1.217384
22	1	0	3.025374	3.069493	-1.329718
23	1	0	2.517885	4.481646	-0.283023
24	1	0	0.078814	3.881364	-0.747712
25	1	0	0.657964	2.482685	-1.776537
26	1	0	-0.694595	1.599825	0.220384
27	1	0	-3.204984	-0.009013	1.922365
28	1	0	-3.293877	-1.616733	1.185333
29	1	0	-2.602692	1.718113	-1.239690
30	1	0	-2.819415	1.961682	0.506821
31	1	0	-5.333128	-1.009871	-0.021740
32	1	0	-5.541922	-0.758867	1.720578
33	1	0	-4.858767	2.566191	-0.698094
34	1	0	-4.924332	0.967515	-1.458284
35	1	0	-6.333914	1.101878	0.460170
36	8	0	4.057130	1.759257	0.692179
37	1	0	4.942271	1.371395	0.782317
38	8	0	4.916299	-0.809140	0.456443
39	1	0	4.986707	-1.774059	0.362335
40	8	0	0.535610	-3.419991	-0.539823
41	1	0	-0.296791	-3.884632	-0.714345
42	8	0	-2.010255	-2.681708	-0.599928
43	1	0	-2.825956	-2.251226	-0.899793

B7		q(A)	q(A+1)	q(A-1)	fk+	fk-	fk0
1	O	-0.787559	-0.617396	-0.948724	0.16	0.17	0.17
2	N	0.11775	0.213773	0.039033	0.08	0.10	0.09
3	N	-0.184589	-0.149111	-0.261442	0.08	0.04	0.06
4	N	-0.385213	-0.384789	-0.385679	0.00	0.00	0.00
5	C	-0.235952	-0.261095	-0.19165	0.04	0.03	0.03
6	C	-0.402655	-0.401549	-0.405197	0.00	0.00	0.00
7	C	-0.184737	-0.173878	-0.198214	0.01	0.01	0.01
8	C	0.159359	0.127207	0.141091	0.02	0.03	0.01
9	C	0.189185	0.228172	0.113818	0.08	0.04	0.06
10	C	0.321163	0.430636	0.192444	0.13	0.11	0.12
11	C	-0.445547	-0.380454	-0.501506	0.06	0.07	0.06
12	C	0.084178	0.067419	0.054771	0.03	0.02	0.01
13	C	-0.283647	-0.30457	-0.218625	0.07	0.02	0.04
14	C	-0.031819	-0.044453	-0.023728	0.01	0.01	0.01
15	C	-0.006256	0.10636	-0.008867	0.00	0.11	0.06
16	C	0.372738	0.335128	0.338147	0.03	0.04	0.00
17	C	-0.311141	-0.30663	-0.335369	0.02	0.00	0.01
18	C	-0.559523	-0.55099	-0.565738	0.01	0.01	0.01
19	C	0.178962	0.226195	0.096934	0.08	0.05	0.06
20	C	0.034676	0.063666	0.029132	0.01	0.03	0.02
21	H	0.205431	0.23028	0.182298	0.02	0.02	0.02
22	H	0.18519	0.19527	0.176159	0.01	0.01	0.01
23	H	0.177615	0.186034	0.16957	0.01	0.01	0.01
24	H	0.185694	0.195412	0.176723	0.01	0.01	0.01
25	H	0.19275	0.203433	0.183464	0.01	0.01	0.01
26	H	0.204118	0.227833	0.176253	0.03	0.02	0.03
27	H	0.183185	0.187768	0.171242	0.01	0.00	0.01
28	H	0.204483	0.207994	0.195921	0.01	0.00	0.01
29	H	0.192689	0.197139	0.184412	0.01	0.00	0.01
30	H	0.191701	0.196782	0.18114	0.01	0.01	0.01
31	H	0.164252	0.166242	0.159873	0.00	0.00	0.00
32	H	0.182074	0.18404	0.177723	0.00	0.00	0.00
33	H	0.184394	0.186407	0.180068	0.00	0.00	0.00
34	H	0.165524	0.167735	0.161017	0.00	0.00	0.00
35	H	0.283164	0.284583	0.280431	0.00	0.00	0.00
36	O	-0.390684	-0.337487	-0.445461	0.05	0.05	0.05
37	H	0.39629	0.406851	0.383486	0.01	0.01	0.01
38	O	-0.562048	-0.427085	-0.587193	0.03	0.13	0.08
39	H	0.36012	0.383495	0.350642	0.01	0.02	0.02
40	O	-0.528613	-0.49595	-0.564797	0.04	0.03	0.03
41	H	0.375465	0.384735	0.363979	0.01	0.01	0.01
42	O	-0.580453	-0.551624	-0.599071	0.02	0.03	0.02
43	H	0.388286	0.396472	0.381492	0.01	0.01	0.01

B5

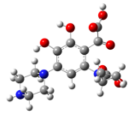

Standard orientation:

Center Number	Atomic Number	Atomic Type	Coordinates (Angstroms) X	Y	Z
1	8	0	3.408155	0.241257	-0.760625
2	7	0	1.474153	-1.729348	-0.190353
3	7	0	-3.037042	0.098776	0.223206
4	7	0	-5.727097	-0.811330	-0.120286
5	6	0	1.697387	-2.458605	1.038568
6	6	0	3.070668	-2.449871	1.637237
7	6	0	1.964266	-1.664605	2.278565
8	6	0	0.597227	-0.603942	-0.186052
9	6	0	1.987998	-2.131223	-1.374644
10	6	0	1.111770	0.699202	-0.282119
11	6	0	-0.753410	-0.818768	-0.009761
12	6	0	-1.652496	0.253506	0.042343
13	6	0	-3.832204	0.464362	-0.969536
14	6	0	-3.473477	-1.207605	0.725538
15	6	0	2.555157	0.969803	-0.302459
16	6	0	-5.303672	0.500235	-0.615389
17	6	0	-4.947795	-1.151831	1.072110
18	6	0	0.212136	1.770044	-0.231440
19	6	0	-1.148149	1.552256	-0.062603
20	1	0	1.109831	-3.362529	1.139049
21	1	0	3.823741	-1.890606	1.098787
22	1	0	3.407345	-3.360793	2.111998
23	1	0	1.530601	-2.034549	3.196650
24	1	0	1.998851	-0.586634	2.182321
25	1	0	-1.110173	-1.836234	0.072353
26	1	0	-3.655500	-0.276690	-1.760751
27	1	0	-3.515732	1.437237	-1.342003
28	1	0	-2.893641	-1.456280	1.614885
29	1	0	-3.310365	-1.990040	-0.027846
30	1	0	-5.465474	1.290335	0.131084
31	1	0	-5.877141	0.753255	-1.507117
32	1	0	-5.261382	-2.128521	1.440290
33	1	0	-5.095757	-0.419937	1.877595
34	1	0	-6.704286	-0.749134	0.147384
35	8	0	2.788669	-3.202633	-1.259175
36	1	0	3.098179	-3.429168	-2.149416
37	8	0	0.688928	3.032755	-0.354962
38	1	0	-0.021385	3.666006	-0.169190
39	8	0	-1.933880	2.667056	0.018819
40	1	0	-2.767193	2.436926	0.458664
41	6	0	3.049781	2.204542	0.463443
42	8	0	2.804470	2.359339	1.632015
43	8	0	3.837442	2.973854	-0.267573
44	1	0	4.180811	3.702156	0.279853
45	8	0	1.749648	-1.601189	-2.449169

B5		q(A)	q(A+1)	q(A-1)	fk+	fk-	fk0
1	O	-0.398436	-0.353685	-0.659111	0.26	0.04	0.15
2	N	-0.029366	0.00887	-0.026224	0.00	0.04	0.02
3	N	-0.120544	0.144492	-0.189659	0.07	0.27	0.17
4	N	-0.385682	-0.37906	-0.385642	0.00	0.01	0.00
5	C	-0.229471	-0.235926	-0.218337	0.01	0.01	0.01
6	C	-0.337427	-0.337328	-0.330908	0.01	0.00	0.00
7	C	-0.197755	-0.200926	-0.193043	0.00	0.00	0.00
8	C	0.366414	0.441666	0.202411	0.16	0.08	0.12
9	C	0.577772	0.574173	0.60021	0.02	0.00	0.01
10	C	0.57299	0.687507	0.303056	0.27	0.11	0.19
11	C	-0.497252	-0.509722	-0.513504	0.02	0.01	0.00
12	C	-0.048468	-0.090433	-0.037492	0.01	0.04	0.03
13	C	-0.282925	-0.347175	-0.237278	0.05	0.06	0.05
14	C	-0.053138	-0.084249	-0.04044	0.01	0.03	0.02
15	C	0.238576	0.244867	0.166867	0.07	0.01	0.04
16	C	-0.307313	-0.298687	-0.326882	0.02	0.01	0.01
17	C	-0.523258	-0.488021	-0.534471	0.01	0.04	0.02
18	C	0.132953	0.144543	0.210333	0.08	0.01	0.03
19	C	-0.261956	-0.207732	-0.214379	0.05	0.05	0.00
20	H	0.189324	0.194301	0.183328	0.01	0.00	0.01
21	H	0.171348	0.174804	0.167697	0.00	0.00	0.00
22	H	0.168714	0.172605	0.164691	0.00	0.00	0.00
23	H	0.167696	0.170517	0.164023	0.00	0.00	0.00
24	H	0.168426	0.171458	0.164271	0.00	0.00	0.00
25	H	0.203344	0.225649	0.185425	0.02	0.02	0.02
26	H	0.185641	0.243144	0.175876	0.01	0.06	0.03
27	H	0.208035	0.240241	0.201877	0.01	0.03	0.02
28	H	0.197907	0.230956	0.190436	0.01	0.03	0.02
29	H	0.190833	0.248794	0.181755	0.01	0.06	0.03
30	H	0.164545	0.181788	0.1612	0.00	0.02	0.01
31	H	0.182606	0.196888	0.179246	0.00	0.01	0.01
32	H	0.185146	0.200139	0.181709	0.00	0.01	0.01
33	H	0.166424	0.184123	0.162834	0.00	0.02	0.01
34	H	0.28395	0.293385	0.281761	0.00	0.01	0.01
35	O	-0.399557	-0.391575	-0.407871	0.01	0.01	0.01
36	H	0.397942	0.400508	0.394997	0.00	0.00	0.00
37	O	-0.481003	-0.438781	-0.528886	0.05	0.04	0.05
38	H	0.386101	0.393899	0.372048	0.01	0.01	0.01
39	O	-0.559163	-0.460827	-0.575803	0.02	0.10	0.06
40	H	0.386594	0.408917	0.379753	0.01	0.02	0.01
41	C	0.248141	0.235377	0.238039	0.01	0.01	0.00
42	O	-0.45101	-0.437086	-0.542425	0.09	0.01	0.05
43	O	-0.358001	-0.352206	-0.394068	0.04	0.01	0.02
44	H	0.375902	0.3785	0.35642	0.02	0.00	0.01
45	O	-0.5956	-0.578692	-0.613837	0.02	0.02	0.02
